# Systematic Disruption of Zebrafish Fibrillin Genes Identifies a Translational Zebrafish Model for Marfan Syndrome

**DOI:** 10.1016/j.jacbts.2026.101543

**Published:** 2026-04-22

**Authors:** Karo De Rycke, Marina Horvat, Lisa Caboor, Petra Vermassen, Griet De Smet, Sophie Lobbestael, Marta Santana Silva, Wouter Steyaert, Matthias Van Impe, Patrick Segers, Julie De Backer, Patrick Sips

**Affiliations:** a Center for Medical Genetics Ghent (CMGG), Department of Biomolecular Medicine, Ghent University, Ghent, Belgium; b Department of Internal Medicine and Pediatrics, Ghent University, Ghent, Belgium; c Biophysical Models for Medical Applications (BioMMedA), Institute of Biomedical Engineering and Technology (IBiTech), Ghent University, Ghent, Belgium; d Department of Cardiology, Ghent University Hospital, Ghent, Belgium

**Keywords:** animal disease models, aortic disease, bulbus arteriosus, CRISPR/Cas9, extracellular matrix, fibrillinopathies

## Abstract

Fibrillin defects lead to severe cardiovascular complications in Marfan syndrome (MFS), including aortic dilation, dissection, and rupture. To model MFS, zebrafish mutants lacking various fibrillin genes were generated. Among these mutant lines, only fibrillin-3–deficient zebrafish exhibited cardiovascular phenotypes mimicking human disease. Multimodal imaging revealed early cardiac defects, bulbus arteriosus dilation, and valve abnormalities. Transcriptomic analysis identified altered regulation of pathways related to extracellular matrix homeostasis and immune system activation. This zebrafish model, recapitulating key cardiovascular features of MFS, provides a valuable platform to investigate disease mechanisms and identify novel treatment strategies.

## Highlights

• Zebrafish fibrillins were disrupted to create a novel animal model of MFS.
• Detailed cardiovascular phenotyping was applied across both early developmental and adult stages of fibrillin-impaired zebrafish.
• *fbn3*^*–/–*^ zebrafish exhibit aortic dilation and valvular abnormalities resembling the cardiovascular manifestations observed in patients with MFS.
• This *fbn3*^*–/–*^ zebrafish model represents a relevant tool for advancing our understanding of MFS pathogenesis and for identifying urgently needed therapeutic strategies.

## Visual Abstract

**Figure:**
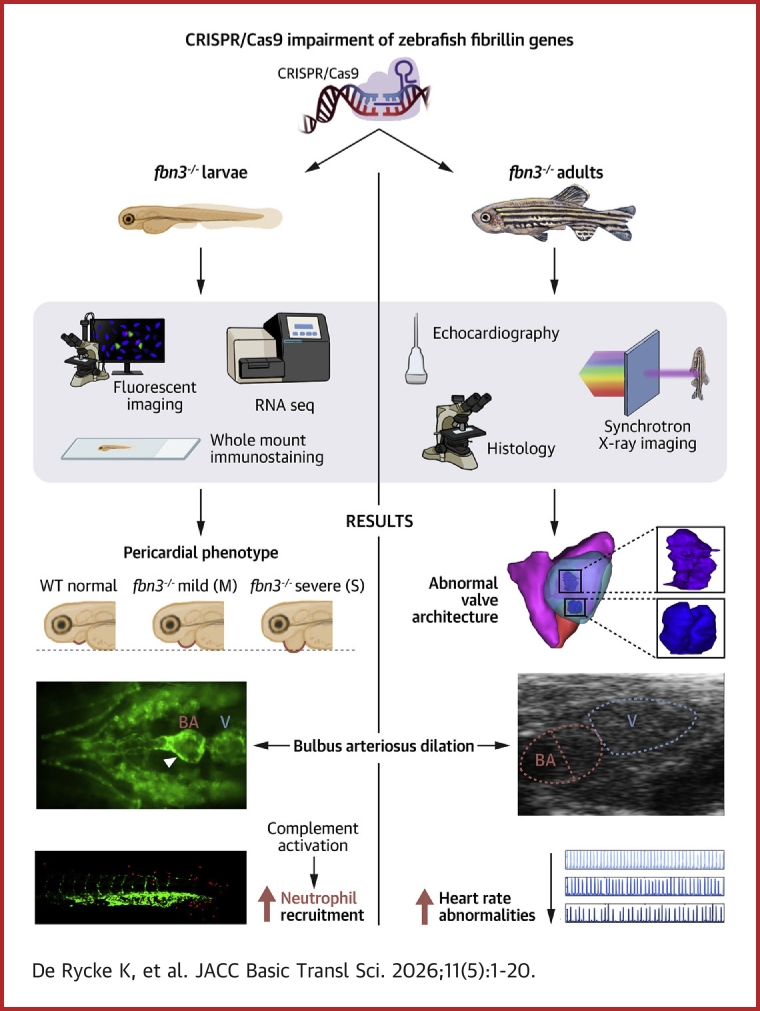

Fibrillin microfibrils are fundamental components of the extracellular matrix that contribute to the integrity of connective tissue in various organs, including blood vessels, lungs, skin, skeleton, and eyes.^1^ These microfibrils can either directly provide stress-bearing structural support to the tissue or play an essential role as a scaffold for tropoelastin deposition, leading to the formation of elastic fibers that provide tensile strength to the extracellular matrix.^2^^,^^3^ In addition to their structural role, fibrillin microfibrils are crucial for tissue mechanobiology and homeostasis through the regulation of the bioavailability of growth factors of the transforming growth factor-β (TGF-β) and bone morphogenetic protein family, as well as by interactions with cell surface receptors such as integrins.^4^

The human genome contains 3 fibrillin isoforms: fibrillin-1 (*FBN1*), fibrillin-2 (*FBN2*), and fibrillin-3 (*FBN3*). Pathogenic variants in *FBN1* and *FBN2* have been associated with connective tissue disorders, including Marfan syndrome (MFS; OMIM #154700) and Beals-Hecht syndrome, also known as congenital contractural arachnodactyly (OMIM #121050), respectively.^5^, ^6^, ^7^ MFS is an autosomal dominant inherited disorder with pleiotropic manifestations, including skeletal abnormalities (eg, skeletal overgrowth, joint laxity), ocular manifestations (eg, ectopia lentis), and skin abnormalities (eg, striae).^1^

In addition to the cardinal cardiovascular manifestations, namely thoracic aortic aneurysm and dissection (TAAD) and mitral valve disease, impaired myocardial function and arrhythmias occur more frequently in patients with MFS. These cardiovascular manifestations contribute to significant morbidity and an increased risk of early mortality in patients with MFS.^8^^,^^9^ Patients with congenital contractural arachnodactyly have an MFS-like skeletal phenotype, but the eyes and aorta are typically not affected. Nevertheless, some cases have been described in which pathogenic variants in human *FBN2* were linked to aortic dilation, indicating a level of functional overlap between different fibrillins.^6^^,^^7^ Noteworthy, syndromes with opposite phenotypes (eg, short instead of tall stature) have been observed as well in patients harboring pathogenic variants in specific domains of *FBN1* and *FBN2*.^10^ To date, the importance of *FBN3* remains less well characterized. However, some associations of *FBN3* variants were reported with polycystic ovary syndrome, Bardet-Biedl syndrome, and Weill-Marchesani syndrome.^11^^,^^12^

Previous research on MFS and other fibrillinopathies has significantly advanced our understanding of their genetic basis and the importance of fibrillin proteins for maintaining extracellular matrix integrity and biomechanical signaling. Nevertheless, the precise molecular mechanisms linking these fibrillin defects to the complex cardiovascular manifestations remain incompletely understood. More flexible in vivo models are therefore needed to address this knowledge gap.

During the last decades, zebrafish (*Danio rerio*) has emerged as a versatile animal model to complement established mammalian models. Although the cardiovascular system of teleosts has a less complex architecture than its mammalian counterpart, mainly due to the lack of pulmonary circulation, it has nonetheless proven to be a valuable model for studying cardiovascular diseases.^13^^,^^14^ Zebrafish have several unique characteristics that make them an attractive disease model: 1) optical transparency during early development, enabling easy intravital microscopic observation; 2) suitability for high-throughput applications due to low cost and high fecundity; and 3) high genetic similarity to humans, with >70% of human coding genes having at least one zebrafish ortholog.^15^ For cardiac pathologies specifically, 96% of the genes known to cause cardiomyopathies are conserved and highly expressed in the zebrafish heart.^16^ These distinctive traits make zebrafish an invaluable model for cardiovascular research, particularly for small-molecule, high-throughput drug screens to discover novel therapeutic targets.^14^ Due to their genetic tractability, zebrafish can also be used to assess the physiological effects of variants of uncertain significance, which would be a significant contribution to improved patient management.^17^^,^^18^

As in humans, the zebrafish genome contains 3 fibrillin genes, termed *fibrillin-1* (*fbn1*), *fibrillin-2* (*fbn2*), and *fibrillin-3* (*fbn3*), although conflicting annotations have been used in different publications and databases. A phylogenetic study conducted by Piha-Gossack et al^19^ found only small evolutionary changes in fibrillin protein structure among different species. The ancestral fibrillin gene already contained most key elements except for the unique characteristic domain located at the N-terminus and specific RGD (Arg-Gly-Asp) motifs.

In the current study, we systematically disrupted the 3 different fibrillin genes in zebrafish using CRISPR/Cas9 technology to examine their impact on the development and function of the cardiovascular system. The findings revealed multiple phenotypes that are pertinent to MFS.

## Methods

All materials and methods are described in detail in the Supplemental Methods. The raw ribonucleic acid (RNA) sequencing data in this publication have been deposited in the National Center for Biotechnology Information’s Gene Expression Omnibus^20^ and are accessible through Gene Expression Omnibus Series accession number GSE300393. All experiments were approved by the Animal Ethics Committee of the Ghent University Faculty of Medicine and Health Sciences (ECD 17-75K, ECD 17-78, and ECD 19-16K) and conform to the guidelines from Directive 2010/63/EU of the European Parliament on the protection of animals used for scientific purposes.

### Statistical Analysis

Continuous data are presented as the mean ± SEM or count (percentage). Group differences of continuous variables were compared by using Student’s *t*-test or the Mann-Whitney *U* test, dependent on normality. Comparisons across multiple groups used one- or two-way analysis of variance with Dunnett’s (comparisons vs a control) or Tukey’s (all pairwise) post hoc tests for multiple comparisons if normally distributed or the Kruskal-Wallis test otherwise. Categorical data (eg, phenotypic distribution) were analyzed by using the Fisher exact test. Kaplan-Meier curves were used to evaluate survival analysis of offspring. A *P* value <0.05 was considered statistically significant, and GraphPad Prism version 10.1.2 (GraphPad Software) was used for all statistical analyses.

Statistical outlier tests to remove individual data points were not applied. Some phenotypes in the study data set exhibit genuine biological heterogeneity and incomplete penetrance (eg, atrioventricular [AV] valve abnormalities ranging from absent to overtly abnormal). Excluding extreme values would therefore remove true biological variation rather than technical artifacts. To accurately represent the full phenotypic spectrum, all samples were retained in the analysis. Normality of continuous data was assessed by using the Shapiro-Wilk test. Data that violated the normality distribution were log-transformed when possible.

## Results

### Protein Homology Between Human and Zebrafish Fibrillin Isoforms

**Figure 1:**
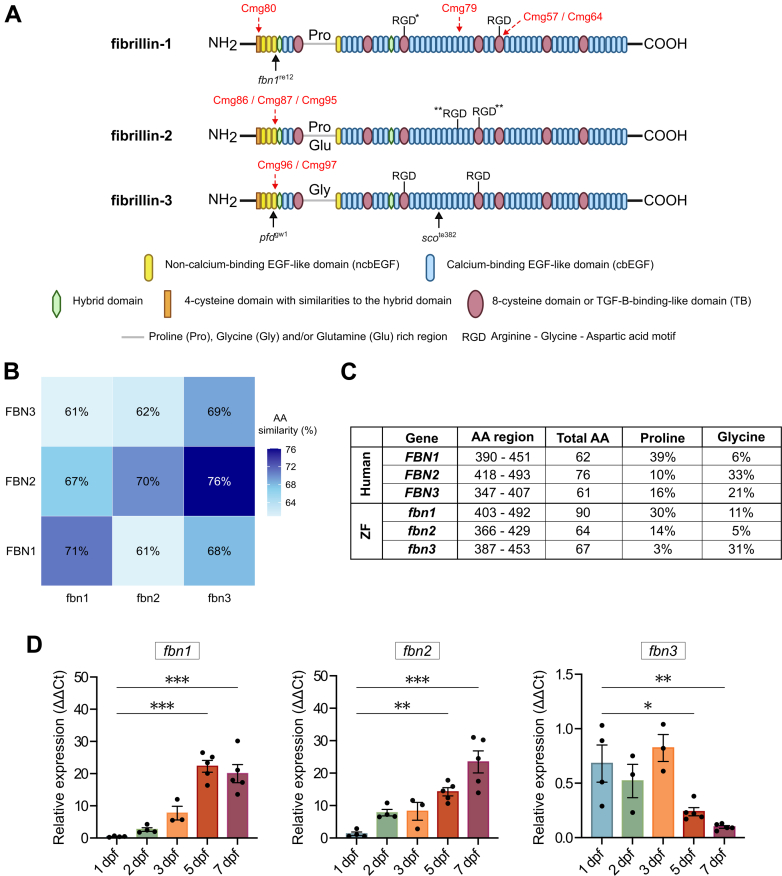
Overview of the Three Fibrillin Isoforms in ZF (A) Schematic representation of the protein domains identified in all 3 zebrafish (ZF) fibrillin isoforms (fibrillin-1, fibrillin-2, and fibrillin-3). Unique proline/glycine/glutamine (Pro/Gly/Glu)–rich regions and the various RGD-motifs are indicated, some of which are exclusively present in ZF (asterisk). CRISPR/Cas9–induced recombination sites are indicated with a red arrow; mutation sites in previously reported ZF models are also annotated with a black arrow. (B) Heatmap showing the percentage of amino acid (AA) sequence similarity between human fibrillin proteins (y-axis) and their ZF orthologs (x-axis). Sequence similarity was calculated by using the Clustal Omega program (version 1.2.4). (C) Comparison of the relative contribution of Pro and Gly in the Pro/Gly–rich region between human and ZF fibrillins. (D) Messenger ribonucleic acid expression pattern of the fibrillin isoforms in wild-type ZF embryos at 1, 2, 3, 5, and 7 days postfertilization (dpf) (n = 3-5 for each developmental stage). Values are mean ± SEM. Statistical test analysis: one-way analysis of variance followed by Dunnett’s multiple comparisons test on log-transformed data. ∗*P* < 0.05, ∗∗*P* < 0.01, ∗∗∗*P* < 0.001. ΔΔCt = comparative threshold cycle.

The protein structure of the human fibrillin family has been well documented and shows that the various fibrillin genes share an almost identical organization of functional domains. To analyze the zebrafish fibrillin gene family, this study used the genomic *fbn2* and *fbn3* sequences, which were mapped and annotated in the GRCz11 assembly of the zebrafish genome sequence. A genome assembly gap, however, prevented correct annotation of the first 32 exons of the zebrafish *fbn1* gene. Recently, a full-length complementary DNA sequence prediction was assigned to *fbn1* (XM_073930076.1), and this sequence, which aligned with our own complementary DNA sequencing data, was used for in-depth analysis (Supplemental Figure 1). Analysis using InterProScan software (version 96.0) revealed a highly conserved protein domain organization between species, with the characteristic four-cysteine domain, two hybrid domains, and seven 8-cystein or TGF-β binding-like domains present in all 3 zebrafish fibrillin proteins, as schematically represented in Figure 1A. Consequently, the zebrafish fibrillins present a high level of homology to human fibrillin proteins with an average overlap in amino acid sequence of 70% (Figure 1B).

A characteristic domain within all fibrillins is the proline and/or glycine-rich region immediately following the first TGF-β binding-like domain. Comparing these domains between species revealed similarities in amino acid composition between *FBN1* and *fbn1*, *FBN2*, and *fbn3*, and, to some extent, *FBN3* and *fbn2* (Figure 1C). Furthermore, the RGD-integrin binding sites present in all human fibrillin isoforms are highly conserved in the zebrafish fibrillins, except for zebrafish fibrillin-2*,* which lacks all RGD motifs. Interestingly, fibrillin-1 in zebrafish contains an extra RGD motif in the third TGF-β binding-like domain.

### Expression Pattern of the Different Fibrillin Isoforms in Wild-Type Zebrafish

Real-time quantitative PCR (qPCR) was used to measure expression levels of *fbn1*, *fbn2*, and *fbn3* in wild-type (WT) whole-embryo tissue at different developmental stages (1, 2, 3, 5, and 7 days postfertilization [dpf]). The results showed that both *fbn1* and *fbn2* were expressed more abundantly later in development, whereas *fbn3* expression levels were highest at the early stages and gradually decreased with age (Figure 1D).

### Normal Cardiovascular Development in Zebrafish Lacking *fbn1* and/or *fbn2*

**Figure 2:**
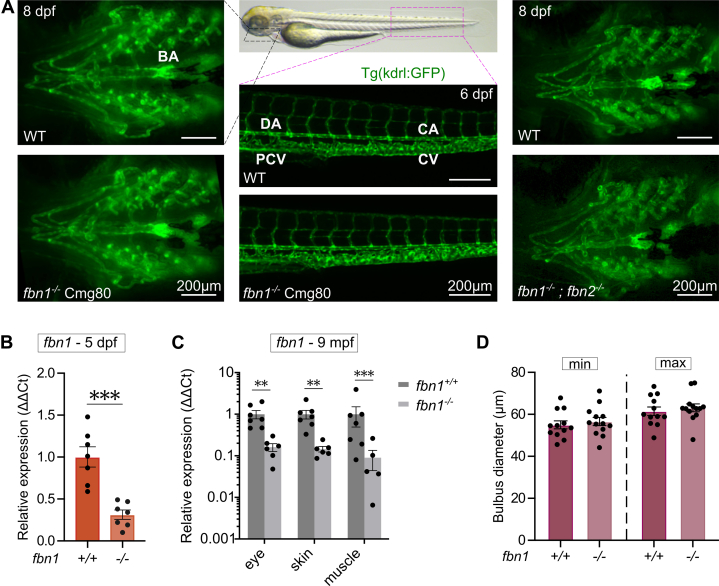
Cardiovascular Architecture in *fbn1* and/or *fbn2* Mutants Fluorescent images of the vasculature of *fbn1*^*–/–*^ (Cmg80) ZF with or without the additional loss of *fbn2* at 8 dpf showed no phenotypic differences from wild-type (WT) ZF. (A, left and right) Ventral view of the ventral aorta and bulbus arteriosus (BA) of 6 to 8 dpf WT, *fbn1*^*–/–*^, and *fbn1*^*–/–*^*; fbn2*^*–/–*^ larvae, respectively. (A, middle) Lateral view of the distal part of the dorsal aorta (DA) merging into the caudal aorta (CA) as well as the posterior cardinal vein (PCV) merging into the caudal vein (CV) in 6 dpf WT and *fbn1*^*–/–*^ larvae. (B) Real-time quantitative PCR analysis of *fbn1* expression in 5 dpf WT and *fbn1*^*–/–*^ (Cmg80) larvae (n = 7). Each data point represents the mean of 2 technical repeats. (C) Real-time quantitative PCR analysis of *fbn1* expression in eye, skin, and muscle tissue of 9 months postfertilization (mpf) WT and *fbn1*^*–/–*^ (Cmg80) ZF (n = 5-7). (D) Quantification of BA diameters in 7 dpf *fbn1*^*–/–*^ (Cmg80) and matched WT controls during minimal (min) and maximal (max) distension (n = 12-13). Values are mean ± SEM. Statistical analysis: unpaired *t* test (B) and two-way analysis of variance followed by Tukey’s multiple comparisons test (C and D). ∗∗*P* < 0.01, ∗∗∗*P* < 0.001. Other abbreviations as in Figure 1.

Stable mutant lines of 4 independent *fbn1* knock-out (KO) zebrafish models were generated, carrying either a frameshift deletion or insertion in exon 2, 34, or 38, as summarized in Supplemental Table 1. Surprisingly, all *fbn1* homozygous mutant (*fbn1*^*–/–*^) zebrafish survived normally to adulthood, without any cardiovascular phenotype during development (Figure 2A). Loss of *fbn2*, with or without *fbn1* deficiency, also did not influence cardiovascular development or survival. Real-time qPCR expression analysis showed a significant reduction in *fbn1* messenger RNA expression in *fbn1*^*–/–*^ zebrafish compared with WT siblings, observed in both 5 dpf whole larvae (Figure 2B) and in adult (9 mpf) ocular, skin, and muscle tissues (Figure 2C). There was no difference in the bulbus arteriosus (BA) diameter of *fbn1*^*–/–*^ mutants compared with WT controls (Figure 2D).

*fbn2* and *fbn3* messenger RNA expression was unchanged as well in *fbn1*^*–/–*^ larvae (Supplemental Figures 2A to 2C). In addition, we confirmed that all 3 fibrillin genes are expressed in the embryonic heart, using cardiac tissue isolated from 2 dpf and 5 dpf larvae (Supplemental Figure 3). Consistent with the whole-larvae data, no significant differences in *fbn2* or *fbn3* expression were observed between WT and *fbn1*^*–/–*^ hearts, indicating that cardiac-specific genetic compensation does not occur in the mutant background.

Transthoracic echocardiography performed on adult *fbn1* and/or *fbn2* mutant zebrafish of various ages (6-19 months postfertilization [mpf]) also showed no significant abnormalities in any measured cardiovascular parameters (Supplemental Tables 4 to 6).

### Variable Endocardial Phenotype in *fbn3*^*–/–*^ Zebrafish

**Figure 3:**
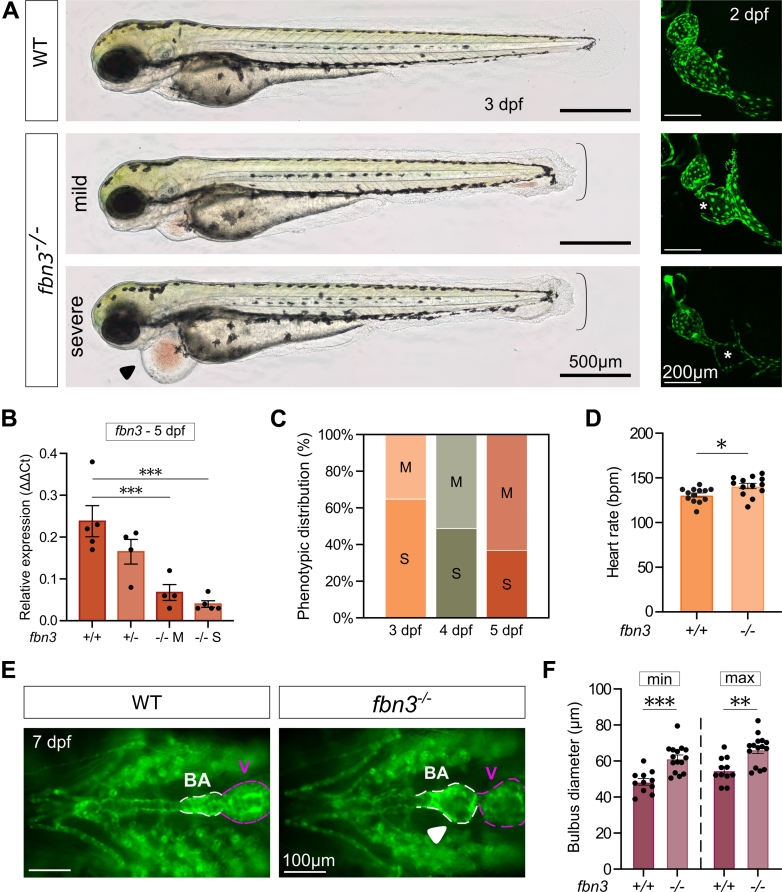
Cardiovascular Development in *fbn3* Mutants Representative images of the diverse phenotypes observed in 2 to 7 dpf *fbn3*^*–/–*^ (Cmg96) mutants and WT controls. (A, left) Lateral whole-embryo view of 3 dpf larvae using brightfield microscopy. Accolade indicates fin-fold atrophy; black arrowhead indicates severe pericardial edema. (A, right) Reconstructed three-dimensional in vivo two-photon fluorescent images of the non-beating heart of 2 dpf *Tg(kdrl:GFP)* WT ZF and *fbn3*^*–/–*^ ZF. Endocardial detachment (asterisk) is observed in the atrium of the *fbn3*^*–/–*^ ZF with pericardial edema. (B) Messenger ribonucleic acid expression levels of *fbn3* in WT, *fbn3*^*+/–*^, and *fbn3*^*–/–*^ (mild or severe) ZF at 5 dpf (n = 4-5). (C) Phenotypic distribution (%) of *fbn3*-deficient ZF presenting a mild (M) or severe (S) pericardial phenotype and their dynamics over time (3, 4, and 5 dpf) (n = average of 19 clutches; 50-100 embryos per clutch). (D) Quantification of the average heart rate in beats per minute (bpm) of 3 dpf *fbn3*^*–/–*^ with preserved endocardial integrity and matching controls (n = 13). (E) Ventral view of 7 dpf *Tg(kdrl:GFP)* WT and *fbn3*^*–/–*^ ZF with preserved endocardial integrity. White arrowhead indicates dilated BA. (F) Quantification of BA diameters at 7 dpf during minimal (min) and maximal (max) distension (n = 11-15). Each data point represents the mean of 2 technical replicates. Statistical analysis: one-way analysis of variance followed by Tukey’s multiple comparisons test on log-transformed data (B), Fisher exact test C, unpaired Student’s *t*-test D, and two-way analysis of variance followed by Tukey’s multiple comparisons test F. Values are mean ± SEM. ∗*P* < 0.05, ∗∗*P* < 0.01, ∗∗∗*P* < 0.001. V = ventricle; other abbreviations as in Figures 1 and 2.

We next generated a stable zebrafish mutant line of a 4 bp frameshift deletion located in exon 4 of the *fbn3* gene, resulting in a premature termination codon (p.Cys153∗) (Figure 3A). Real-time qPCR expression analysis of *fbn3*^*–/–*^ larvae (1, 2, 3, 5, and 7 dpf) showed a strong and significant reduction of *fbn3* messenger RNA expression compared with WT siblings, likely due to nonsense-mediated decay (Figure 3B); there were no changes in *fbn1* or *fbn2* expression (Supplemental Figures 4A to 4C).

Starting at 24 hours postfertilization (hpf), *fbn3* homozygous mutant (*fbn3*^*–/–*^) zebrafish embryos could be distinguished from their heterozygous and WT siblings by the presence of fin-fold atrophy (Figure 3A), a phenotype consistent with earlier reports in the *puff daddy* (*pfd*^*gw1*^)^21^ ENU *fbn3* mutant and in *fbn3*-morpholinos.^22^ In approximately 40% of *fbn3*^*–/–*^ offspring, marked pericardial edema was observed, associated with gaps in the endocardium that progressed to complete endocardial detachment in the atrium, similar to what was previously reported for the *scotch tape* ENU *fbn3* mutant (Figure 3A).^23^ *fbn3*^*–/–*^ zebrafish with this severe phenotype do not survive past 6 to 8 dpf due to vascular embolism. The remaining *fbn3*^*–/–*^ larvae exhibited a milder phenotype in which the endocardium remained attached to the myocardium, circulation was maintained, and normal survival to adulthood was observed (Figure 3A). Interestingly, we also observed that a subset of *fbn3*^*–/–*^ embryos with severe pericardial edema (approximately 30%) seemed to recover between 3 and 5 dpf, reverting to the milder phenotype (Figure 3C).

### Bulbus Arteriosus and Cardiac Function in *fbn3*^*–/–*^ Zebrafish Larvae

Several cardiac function parameters were tested by using brightfield microscopy; a mild but significant increase in heart rate was found in mild *fbn3^–/–^* zebrafish (without pericardial edema) compared with WT controls at 3 dpf (Figure 3D, Supplemental Figure 5). Interestingly, the surviving *fbn3*^*–/–*^ zebrafish in which the endocardium remained attached developed dilatation of the bulbus arteriosus (BA), a structure that is considered to be evolutionarily related to the aortic root and ascending aorta in humans,^24^^,^^25^ starting at 5 dpf (Figures 3E and 3F).

### Reduced Venous and Cardiac Endothelial Integrity in *fbn3*^*–/–*^ Zebrafish Larvae

**Figure 4:**
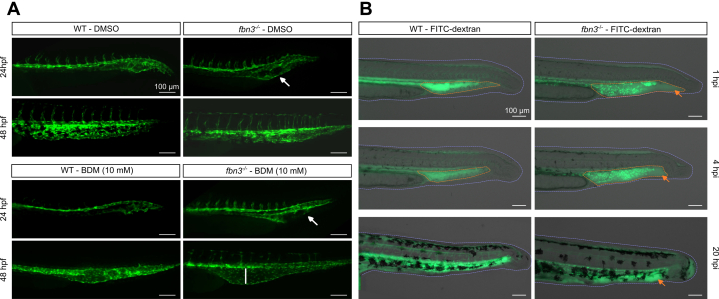
Abnormal Caudal Vein Development and Increased Vascular Permeability in *fbn3*^*–/–*^ Embryos (A) Caudal vein formation in *Tg(kdrl:GFP)* WT and mild *fbn3*^*–/–*^ ZF at 24 and 48 hours postfertilization (hpf) after exposure to 1% dimethyl sulfoxide (DMSO) vehicle (top) or 10 mM 2,3-butanedione monoxime (BDM) to inhibit cardiac contraction (bottom). White arrow indicates abnormal development of the caudal vein. Exposure to BDM leads to more severe caudal vein dilatation (white line) in *fbn3*^*–/–*^ embryos than in WT controls at 48 hpf (qualitative analysis). (B) The caudal vasculature of 500 kDa fluorescein isothiocyanate (FITC)-dextran–injected 30 hpf WT and *Tg(globin:GFP) fbn3*^*–/–*^ embryos, imaged at 1, 4, and 20 hours postinjection (hpi) to assess vascular integrity. Orange dotted lines indicate intravenously injected dextran localization, and the red arrow indicates extravascular dye leakage. The purple dotted line outlines the embryo tail. Scale bar: 100 μm. Other abbreviations as in Figures 1 and 2.

In addition to the BA phenotype, we observed that the caudal vein of *fbn3*^*–/–*^ zebrafish initially developed as a dilated, cavernous venous structure lacking vessel integrity, similar to the early vascular defect previously described in the *pfd*^*gw1*^ mutant.^21^ Although this phenotype persisted in the *fbn3* mutants with the severe endocardial phenotype, in the mild *fbn3* mutants, the caudal vein was shown to remodel appropriately, with a normalized diameter and restored endothelial barrier function (Figure 4A). This led us to investigate the role of blood flow dynamics further by pharmacologically inhibiting cardiac contraction using a myosin inhibitor. This prevented the resolution of the pronounced caudal vein dilatation in the mild *fbn3*^*–/–*^ zebrafish (Figure 4A, Video 1, Video 2, Video 3, Video 4).

Reduced caudal vein endothelial barrier integrity was further assessed by intravenous injection of fluorescein isothiocyanate–labeled dextran. In 1 dpf *fbn3*^*–/–*^ embryos, extravascular dye leakage was evident in the area below the caudal vein as early as 1 hour postinjection (Figure 4B, Video 5, Video 6, Video 7, Video 8). In parallel, using a *Tg(globin:GFP)* transgenic zebrafish line, erythrocyte leakage was detected in the heart of severely affected fbn3^-/-^ embryos exhibiting endocardial detachment, with accumulation in the pericardial sac. (Supplemental Figure 6A, Videos 9 and 10). Leakage of erythrocytes from the caudal vein and accumulation in the tail region were observed as well, where their degradation produced a diffuse fluorescent signal (Supplemental Figure 6B, Videos 11 and 12).

### qPCR Confirms the Absence of Compensation Mechanisms by *fbn1* and/or *fbn2*

To test whether other fibrillin isoforms compensate for a lack of *fbn3* expression in the *fbn3*^*–/–*^zebrafish, the expression levels of *fbn1*, *fbn2*, and *fbn3* were measured in WT, *fbn3*^*+/–*^, *fbn3*^*–/–*^ mild, and *fbn3*^*–/–*^ severe whole embryos during early development (1, 2, 3, 5, and 7 dpf). No significant differences in *fbn3* expression levels were observed between the mild and severe *fbn3*^*–/–*^ phenotypes (Supplemental Figure 4C). Also, no differences were observed in *fbn1* and *fbn2* expression levels between *fbn3*^*–/–*^ and matching controls (Supplemental Figures 4A and 4B). These findings suggest that increased expression of *fbn1* or *fbn2* does not compensate for the lack of *fbn3* expression.

### Triple Fibrillin KOs Do Not Survive to Adulthood

**Figure 5:**
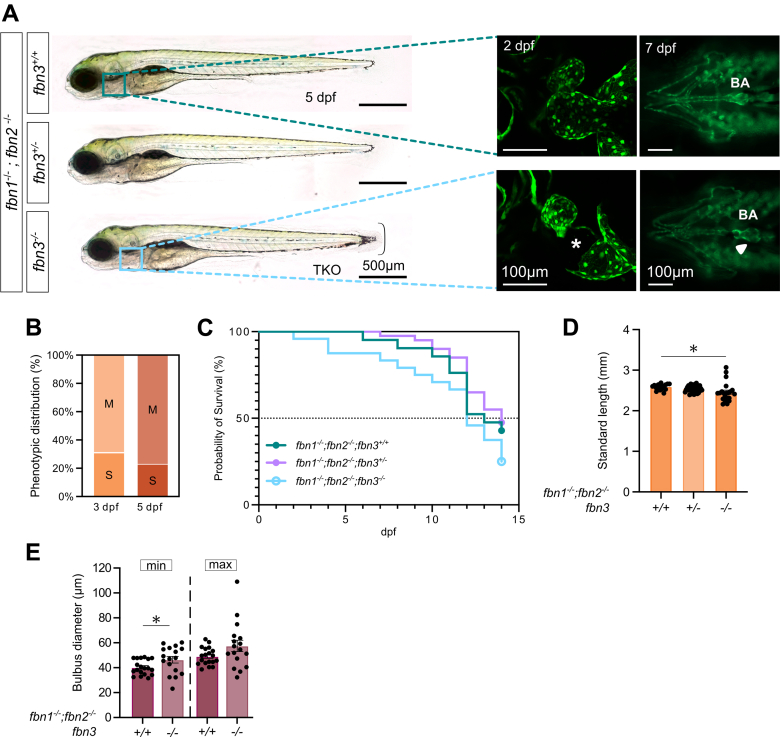
Phenotypic Features of TKO Larvae Representative images of the phenotypes observed in 3 to 7 dpf *fbn1*^*–/–*^*; fbn2*^*–/–*^*; fbn3*^*+/+*^, *fbn1*^*–/–*^*; fbn2*^*–/–*^*; fbn3*^*+/–*^*,* and *fbn1*^*–/–*^*; fbn2*^*–/–*^*; fbn3*^*–/–*^ larvae. (A, left) Lateral whole-embryo view of a 5 dpf triple fibrillin knockout (TKO) without complete endocardial detachment and sibling controls, using brightfield microscopy. Accolade: fin-fold atrophy. (A, middle) Reconstructed maximum intensity projection in vivo confocal fluorescent images of the heart of 2 dpf *Tg(kdrl:GFP)* WT and TKO ZF. Endocardial detachment (asterisk) is observed in the atrium of a subset of TKO ZF. (A, right) Fluorescent ventral images of 7 dpf *Tg(kdrl:GFP)* TKO and sibling controls with preserved endocardial integrity. White arrowhead indicates dilated BA. (B) Phenotypic distribution (%) of TKO ZF presenting a mild (M) or severe (S) pericardial phenotype and their dynamics over time (3 and 5 dpf) (n = 21). (C) Fourteen-day Kaplan-Meier survival curve of the offspring of an incross of *fbn1*^*–/–*^*; fbn2*^*–/–*^*; fbn3*^*+/–*^ ZF (n = 21-40). (D) Quantification of standard length at 3 dpf (n = 17-36). (E) Quantification of BA diameters at 7 dpf during minimal (min) and maximal (max) distension (n = 17-19). Statistical analysis: Fisher exact test B, Kaplan-Meier survival analysis C, one-way analysis of variance followed by Dunnett’s multiple comparison test D, and two-way analysis of variance followed by Tukey’s multiple comparisons test E. Values are mean ± SEM. ∗*P* < 0.05. Abbreviations as in Figures 1 and 2.

Because loss of *fbn1* and/or *fbn2* does not lead to a phenotype, we investigated whether additional loss of *fbn3* could unmask a functional role for these fibrillins. Consistent with the single *fbn3* KO phenotype, triple KO (TKO) mutants exhibited 100% penetrance of fin-fold atrophy; a subset of TKO larvae exhibited severe pericardial edema due to complete endocardial detachment, whereas the remaining TKO had a milder phenotype (Figures 5A and 5B). Kaplan-Meier survival analysis of offspring from a *fbn1*^*–/–*^*; fbn2*^*–/–*^*; fbn3*^*+/–*^ incross up to 14 dpf exhibited slightly lower survival rates in TKO compared with siblings, although statistical significance was not reached (Figure 5C). To date, we have, however, never detected an adult TKO zebrafish, despite genotyping approximately 300 to 400 adult fish raised from *fbn1*^*–/–*^*; fbn2*^*–/–*^*; fbn3*^*+/–*^ incrosses, indicating the premature mortality of zebrafish with this genotype.

We next assessed the standard length at 3 dpf and found that TKO mutants were significantly shorter than their *fbn1*^*–/–*^*; fbn2*^*–/–*^*; fbn3*^*+/+*^ siblings (Figure 5D). Finally, measurements of the BA diameter at 7 dpf revealed a significant increase in the minimally distended diameter in TKO larvae compared with *fbn1*^*–/–*^*; fbn2*^*–/–*^*; fbn3*^*+/+*^ controls. In the maximally distended state, a larger variability was observed in the TKO group (Figure 5E).

### Transcriptomic Analysis in *fbn3*^*–/–*^ Embryos Suggests Involvement of Extracellular Matrix Remodeling and Immune System Activation

**Figure 6:**
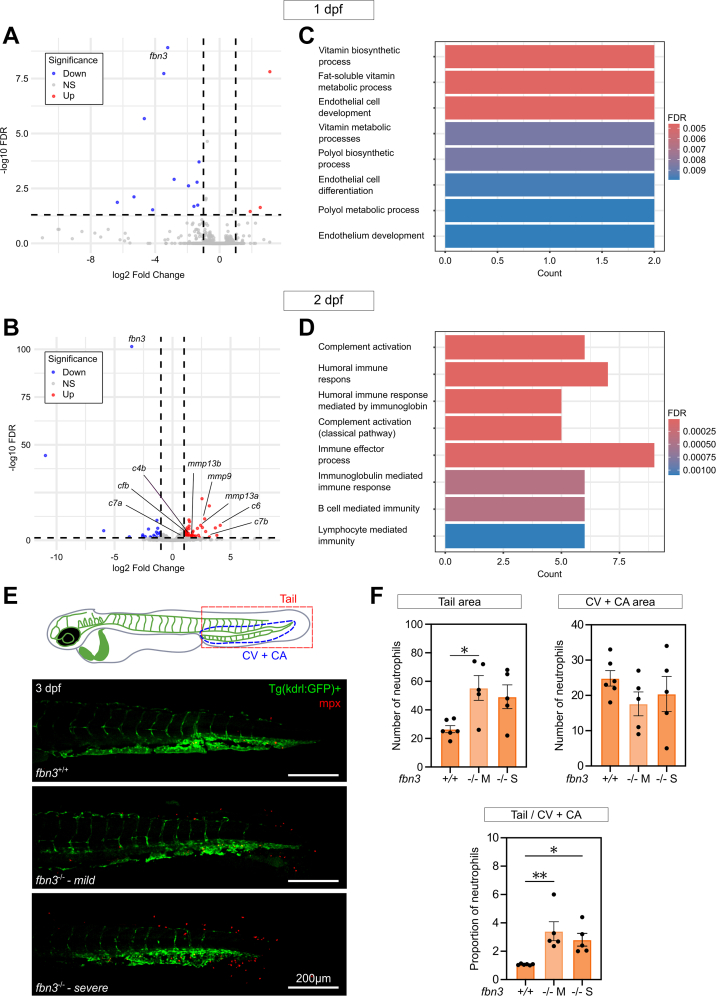
Transcriptomic Analysis of *fbn3*^*+/+*^ and *fbn3*^*–/–*^ Siblings at 1 and 2 dpf (A and B) Volcano plots illustrating differentially expressed genes in *fbn3*^*–/–*^ ZF compared with WT siblings at 1 dpf and 2 dpf, respectively. Up-regulated genes are red, and down-regulated genes are blue, with genes of importance annotated. Thresholds: false discovery rate (FDR) ≥0.05 and |log2 fold change| ≥1. (C and D) Gene ontology enrichment analysis with top 8 hits presented as a bar plot. The x-axis and y-axis represent the gene count and pathway, respectively. Thresholds: FDR ≥ 0.05 and |log2 fold change| ≥1. (E) Confocal maximum intensity projection images of 3 dpf WT and *fbn3*^*–/–*^ embryos displaying either a mild (M) or severe (S) phenotype, whole-mount stained to visualize the immune response. Endothelial cells are labeled by the *Tg(kdrl:GFP)* reporter (green), and neutrophils are labeled by anti-mpx antibody staining (red). Scale bar: 200 μm. (F) Quantification of 3 dpf WT and *fbn3*^*–/–*^ embryos (n = 5-6) showing the total number of neutrophils in the tail region (top left), the number of neutrophils in the circulation in the CV and CA (top right), and the relative proportion between regions (bottom). Statistical analysis: one-way analysis of variance followed by Dunnett’s multiple comparisons test F. For the comparison of the total number of neutrophils in the tail region between WT and *fbn3*^*–/–*^(S), the *P* value was 0.054. Values are mean ± SEM. ∗*P* < 0.05, ∗∗*P* < 0.01. NS = nonsignificant; other abbreviations as in Figures 1 and 2.

Bulk RNA sequencing was conducted on whole *fbn3*^*–/–*^ and WT embryos at 1 and 2 dpf to identify early transcriptional changes during the development of the early cardiovascular phenotypes. Differential expression analysis (adjusted *P* value [false discovery rate] <0.05 and log_2_ fold change [log_2_FC] >1) identified 42 and 361 differentially expressed genes, at 1 and 2 dpf, respectively (Figures 6A and 6B, Supplemental Table 8). At 1 dpf, gene ontology enrichment analysis of the differentially expressed genes revealed significant enrichment in pathways related to metabolism, biosynthesis, and endothelial and blood vessel development (false discovery rate < 0.05) (Figures 6C and 6D, Supplemental Table 8). The latter transcriptional changes were notably driven by strong down-regulation of the *fbn3* transcript (log_2_FC –3.22). By 2 dpf, the transcriptional profile shifted, with gene ontology analysis highlighting enrichment of immune-related processes and defence mechanisms. In particular, several components of the complement system were significantly up-regulated in *fbn3*^*–/–*^ mutants, including *c4b* (log_2_FC 1.37), *c6* (log_2_FC 3.69), *cfb* (log_2_FC 1.11), *c7a* (log_2_FC 0.94), and *c7b* (log_2_FC 3.18). In addition, matrix metalloproteinases (MMPs) were markedly increased, with elevated expression of *mmp9* (log_2_FC 2.77), *mmp13a* (log_2_FC 2.57), and *mmp13b* (log_2_FC 2.85) (Figure 6C).

To functionally validate these immune-related transcriptomic changes and determine whether immune cell recruitment is altered in *fbn3*^*–/–*^ mutants, whole-mount staining was performed for *mpx*, a well-established neutrophil marker (Figure 6E). Three dpf *fbn3*^*–/–*^embryos displayed a clear increase in neutrophil numbers in the tail. Interestingly, the number of neutrophils located within the caudal vein plexus and caudal aorta, which comprise the caudal hematopoietic tissue, was comparable between WT and *fbn3*^*–/–*^ embryos. Conversely, the *fbn3*^*–/–*^ mutants exhibited a more dispersed distribution of neutrophils toward the dorsal periphery of the tail (Figure 6F). To corroborate these findings, we additionally performed live-imaging of *Tg(mpx:GFP)* embryos at 2 and 3 dpf injected with Cas9 protein together with *fbn3* guide RNA. The data confirmed the higher neutrophil abundance in these F0 crispants. Interestingly, the neutrophils in F0 *fbn3* crispants seemed to exhibit a more active interstitial patrolling-like behavior compared with that of noninjected controls (Video 14, Video 15, Video 16, Supplementary Material). At 7 dpf, a similar analysis revealed a trend toward higher neutrophil numbers in the region surrounding the BA of *fbn3*^*–/–*^ embryos (Supplemental Figure 7).

### Echocardiographic and Synchrotron Imaging in Adult *fbn3*^*–/–*^ Zebrafish Hearts Reveal Cardiac Rhythm and Morphologic Abnormalities

**Figure 7:**
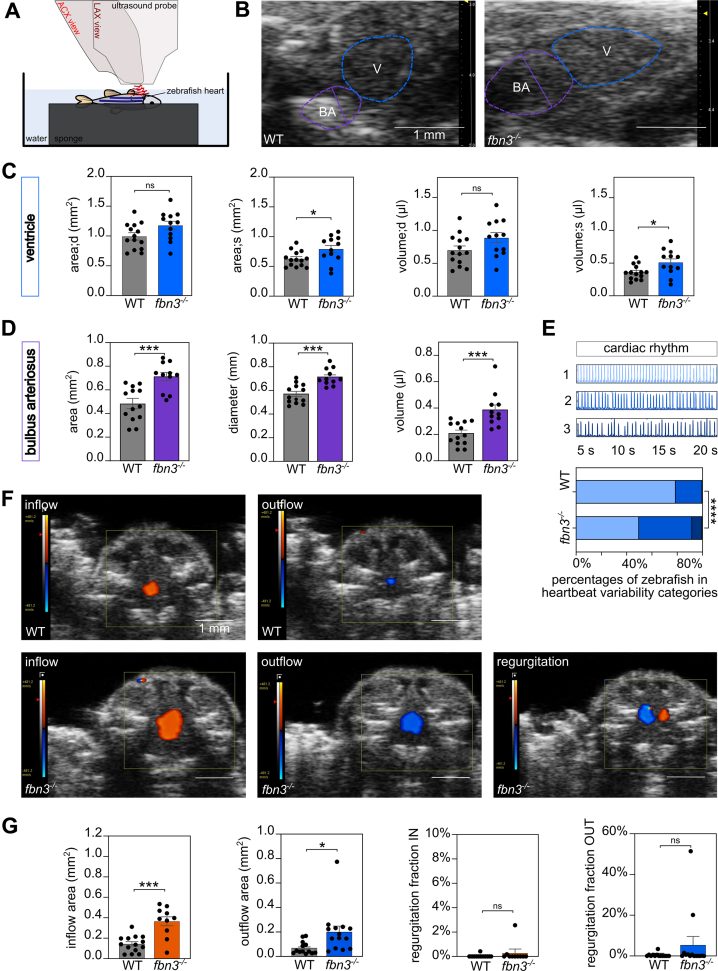
Cardiac Abnormalities of Adult *fbn3*^*–/–*^ ZF (A) Schematic representation of the two-dimensional transthoracic echocardiography of adult ZF. The ZF is anesthetized, positioned with its ventral side upward, and submerged in water. The echocardiography measurements are made with an ultrasound probe, in abdominocranial axis (ACX) view for color flow Doppler (CFD) analysis (red) and in longitudinal view (LCX) for ventricle and BA measurements (dark red). (B) Tracings of the posterior walls of the ventricle (blue) and BA (purple) of WT ZF (left) and *fbn3*^*–/–*^ ZF (right) (6 and 8 mpf). (C) Dimensions of the ventricle during diastole (area;d, volume;d) and systole (area;s, volume;s) (n = 12-14). (D) Measurements of BA volume, area, and diameter while in maximal relaxation (at the time of bulboventricular valve contraction) (n = 11-13). (E) Representative 20-second cardiac rhythm tracings with the associated qualitative score (top). Distribution of cardiac rhythm scores for different genotypes (n = 12) (bottom). (F) CFD recordings of inflow (orange) and outflow (blue) of blood into/from the ventricle in WT ZF (top) and *fbn3*^*–/–*^ ZF (bottom), with an example of regurgitant blood flow seen in some mutants (n = 2 of 14). (G) Quantification of inflow (orange) and outflow (blue) areas and regurgitation fractions from the CFD data (n = 10-14). Values are mean ± SEM. Statistical test analysis: unpaired Student’s *t*-test (C, D, and G, inflow area), Mann-Whitney *U t*est (G, outflow area, regurgitation fraction IN, regurgitation fraction OUT), and the Fisher exact test E. ∗*P* < 0.05, ∗∗*P* < 0.01, ∗∗∗*P* < 0.001. ns = nonsignificant; other abbreviations as in Figures 1 to 3.

Using an optimized in-house cardiac ultrasound setup (Figure 7A), we assessed the cardiovascular phenotype of adult *fbn3*^*–/–*^ zebrafish in vivo. The ventricles of 6- and 8-month-old *fbn3* mutants exhibited mild dilation compared with WT zebrafish (Figures 7B and 7C). In addition, BA of the *fbn3* mutants were significantly dilated (Figures 7B and 7D).

*fbn3*^*–/–*^ and WT zebrafish cardiac rhythm was scored based on echocardiography recordings 20 seconds long. *fbn3*^*–/–*^ zebrafish displayed cardiac rhythm disorders, observed as more irregular or skipped heartbeats (Figure 7E).

Analysis of color flow Doppler recordings revealed increased blood inflow and outflow areas in *fbn3*^*–/–*^ zebrafish. A small number exhibited valvular regurgitation (Figures 7F and 7G).

Using automated processing of pulsed wave Doppler measurements, no significant variations in cardiovascular parameters of systolic, diastolic, and valve function were observed among *fbn3* mutants compared with WT controls (Supplemental Table 6). When comparing the volumes of the atrium, ventricle, or BA between *fbn3*^*–/–*^ and WT zebrafish based on synchrotron imaging, no statistically significant differences were noted. This ex vivo method could not recapitulate the BA dilation, seen on echocardiography performed in vivo, likely due to a lack of intraluminal pressure postmortem. However, while comparing the three-dimensional (3D) heart models of mutants and WT, increased variability was noted in atrial volumes in *fbn3*^*–/–*^ hearts compared with WT hearts (F test, *P* = 0.011); hearts are shown in Supplemental Figure 8, and BA are presented in Supplemental Figure 9.

### Abnormal Cardiac Valve Architecture in Adult *fbn3*^*–/–*^ Zebrafish

**Figure 8:**
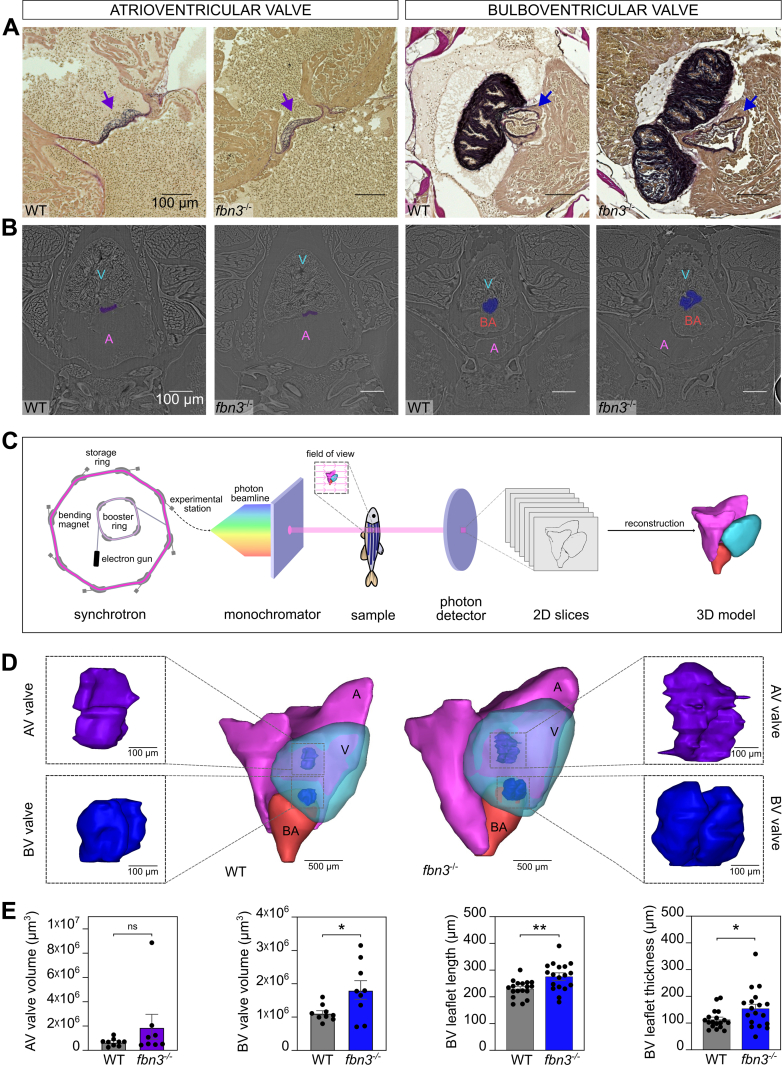
Abnormal Cardiac Valve Architecture of Adult *fbn3*^*–/–*^ ZF (A) Histologic staining for elastin (purple) of the atrioventricular (AV) valve (left, purple arrow) and bulboventricular (BV) valve (right, blue arrow) of WT ZF and *fbn3*^*–/–*^ ZF (6 and 8 mpf). The architecture of the AV valve is normal in the majority of the samples (representative histology picture), whereas the BV valve leaflets are abnormal in shape in all mutants. (B) Representative images of ZF hearts obtained by synchrotron radiographic scanning. Cardiac valves are shown in color (AV valve, purple; BV valve, blue). (C) Schematic representation of synchrotron radiographic imaging method of ZF samples. (D) Representative three-dimensional (3D) models of WT ZF (left) and *fbn3*^*–/–*^ ZF (right) (16 and 18 mpf). Enlarged models of cardiac valves are shown to display the differences between WT and mutants. (E) Volume, length, and thickness measurements of AV (purple) and BV (blue) valves obtained from the 3D models (n = 9). Values are mean ± SEM. ∗*P* < 0.05, ∗∗*P* < 0.01. Statistical test analysis: Mann-Whitney *U* test (AV valve volume), unpaired Student’s *t*-test (BV valve volume, BV leaflet length, and BV leaflet thickness). 2D = 2-dimensional; A = atrium; other abbreviations as in Figures 1 to 3 and 7.

Using histologic staining for elastin of heart sections from 6- and 8-month-old *fbn3* mutant zebrafish, a cardiac valve phenotype was found that is common in all *fbn3* mutants. Specifically, all *fbn3*^*–/–*^ zebrafish displayed abnormalities in the bulboventricular (BV) valve, with one or both leaflets either more thickened or abnormally shaped, compared with the valves of WT zebrafish (Figure 8A). The majority of the *fbn3*^*–/–*^ zebrafish had no altered morphology of the AV valves.

We next performed synchrotron radiographic imaging of *fbn3*^*–/–*^ and WT zebrafish (representative slices through the heart with highlighted valves are presented in Figure 8B) and generated 3D reconstructions of these data. Schematic representations are presented in Figures 8C and 8D; AV valves are shown in Supplemental Figure 10 and BV valves in Supplemental Figure 11. We found that *fbn3*^*–/–*^ zebrafish have larger BV valve volumes with longer and thicker leaflets than WT zebrafish, confirming abnormalities seen on histology. Conversely, volumes of AV valves of mutants did not exhibit a significant difference compared with WT zebrafish. Nevertheless, a small subset (2 of 12) of *fbn3*^*–/–*^ zebrafish had extremely aberrant AV valve morphology, with abnormal folding and fusing of various leaflet segments (Figure 8E).

Elastin staining did not reveal any overt breaks or dissections in the elastic layers of the BA or aorta in *fbn3*^*–/–*^ zebrafish (Supplemental Figure 12A). Likewise, the collagen layer surrounding the BA and aorta appeared comparable in thickness to that observed in WT controls (Supplemental Figures 12B and 12C).

Although most *fbn3*^*–/–*^ zebrafish exhibited preserved cardiac function despite BV valve and BA abnormalities, we tracked a mutant that represents a severely affected phenotype (as seen in approximately 2 of 12 mutants) across multiple imaging modalities. Pulse wave Doppler and color flow Doppler imaging revealed pronounced BV valve regurgitation (Supplemental Figure 13A). Synchrotron-based 3D reconstruction of the same fish showed a markedly enlarged atrium, relatively small ventricle and BA, and highly dysmorphic AV and BV valves (Supplemental Figure 13B). Corresponding histology and synchrotron slices confirmed consistent leaflet malformations (Supplemental Figure 13C), including hypertrophic valve interstitial cells of the AV valve (Supplemental Figure 13D).

## Discussion

Due to sustained advances in diagnostics, longitudinal care, and both medical and surgical interventions, the life expectancy of individuals with MFS has improved dramatically over the past 3 decades.^26^ Although mechanistic insights from mouse models have informed new therapies,^27^ current medical treatments mainly attenuate aortic enlargement, with no shown effect on preventing aortic dissection. In addition, fundamental questions concerning early developmental processes in MFS, tissue-specific dynamics, impact of genetic variants, and targetable disease mechanisms remain unresolved. There is a growing need for complementary vertebrate models that allow higher-throughput in vivo functional studies to capture aspects not accessible in mice. We believe that these issues can be addressed by the new *fbn3* mutant zebrafish model, which presents a consistent, early-onset MFS-like phenotype, including aortic dilation, valvular defects, and arrhythmia.

### Zebrafish *fbn1* Deficiency: A Critical Look at Cardiovascular Phenotype Reproducibility

Despite the high level of conservation between zebrafish and human fibrillins, findings in the current study suggest that the zebrafish *fbn1* isoform may not be the direct functional ortholog of human *FBN1*, as loss of *fbn1* did not result in a cardiovascular phenotype. These results contrast with earlier studies that described mild cardiovascular abnormalities in *fbn1*-deficient zebrafish.^28^, ^29^, ^30^ Specifically, morpholino knockdown of *fbn1* resulted in abnormal vessel dilatation in the head and a lack of venous plexus remodeling.^28^ However, these effects were only seen at relatively high morpholino concentrations. This raises concerns about potential off-target effects and dose-dependent toxicity.^31^ Another study used CRISPR/Cas9 to generate frameshift indels in *fbn1* exon 40 (labeled as exon 16 in the publication due to the incomplete genomic annotation of *fbn1*) and reported limited data indicating body elongation, reduced pigmentation, and altered cardiac blood flow in *fbn1*^*+/–*^.^29^ More recently, an independent group used CRISPR/Cas9 to model a likely pathogenic nonsense variant in exon 60 of *fbn1* (labeled as exon 19 in the publication).^30^ They reported a range of phenotypes in their *fbn1*^*+/–*^ mutants, including pericardial edema, tail curvature, aortic arch bleeding, decreased cardiac function, angiogenesis abnormalities, and increased dorsal aortic diameter; no quantitative data were presented, however.

We were unable to replicate these results in any of the 4 independently generated stable *fbn1* mutant lines. Genetic background is known to influence cardiovascular severity in MFS mouse models,^32^ and thus strain differences between studies (TU vs AB in ours) could be a possible confounding factor. Nevertheless, this alone is unlikely to explain all discrepancies, and further independent studies will be needed to clarify the divergent outcomes of zebrafish *fbn1* deficiency.

### Lack of Compensatory Mechanisms Underscores Fibrillin Gene Complexity in Zebrafish

A possible explanation for the absence of a reproducible phenotype in our *fbn1* mutant models could be that transcriptional adaptation leads to compensatory changes in expression of related genes.^33^ Our qPCR analysis indicated nonsense-mediated decay of mutant *fbn1* transcripts in whole embryos and adult tissue, with a similar trend in isolated embryonic hearts. Importantly, neither whole-embryo nor isolated-heart data sets exhibited compensatory up-regulation of *fbn2* or *fbn3*, arguing against transcriptional adaptation. Instead, the lack of any detectable cardiovascular phenotype in our *fbn1*^*–/–*^ zebrafish likely reflects the complexity of the interspecies functional orthology within the fibrillin gene family.

### Lower Homology of Zebrafish *fbn2* to Human Fibrillins

We also investigated the cardiovascular impact of *fbn2* deficiency, alone and in combination with the loss of *fbn1*, but again detected no abnormalities. This finding aligns with the lower amino acid homology between *fbn2* and human fibrillins. Interestingly, zebrafish fibrillin-2 lacks all RGD motifs, which are known to mediate binding affinity toward various integrins,^34^ while it does contain a novel proline/glutamine–rich domain not found in any other fibrillins. These unique structural differences likely reflect its greater evolutionary distance and suggest a unique zebrafish-specific function, which may be less relevant to humans.

### *fbn3* Loss Leads to Developmental Defects

Unlike loss of *fbn1* and/or *fbn2*, disruption of *fbn3* consistently resulted in an early reproducible cardiovascular phenotype reminiscent of MFS, persisting into adulthood. This action suggests that *fbn3* plays a prominent role during early zebrafish development, functionally resembling human *FBN2*, although the effects during early development have a lasting impact throughout the zebrafish’s lifespan. This notion aligns well with our qPCR data, which show that expression of *fbn3* precedes *fbn1* during zebrafish embryogenesis, similar to *FBN2* and *Fbn2* in humans and mice, respectively, suggesting a conserved role for *fbn3* in early developmental processes.^35^

### Comparing CRISPR-Generated *fbn3* Mutants With Existing ENU Models

The *fbn3* mutant zebrafish generated in this study show a morphology similar to the ENU mutants *pfd*^*gw1*^^23^ and *sco*^*te382*^,^36^ which have, respectively, a nonsense or missense mutation in *fbn3*. Similar to these lines, the current study *fbn3* homozygous mutants displayed a characteristic fin-fold atrophy. Unlike *pfd*^*gw1*^ but similar to *sco*^*te382*^, the current mutants did not develop a notochord phenotype. It was later discovered that *pku300* mutations also contribute to the cardiovascular defects in the *sco*^*te382*^ mutant.^36^ The absence of notochord abnormalities in the current model, despite a frameshift near the *pfd*^*gw1*^ locus, therefore suggests that other genetic factors also contribute to the *pfd*^*gw1*^ phenotype and highlights the need for careful validation of forward genetics data.

As in the *sco*^*te382*^ mutant (but unlike *pfd*^*gw1*^), the severe pericardial edema phenotype was not completely penetrant in our *fbn3*^*–/–*^ zebrafish. We hypothesize that the progression to irreversible, lethal endocardial detachment occurs stochastically. As described for *sco*^*te382*^,^23^ all *fbn3*^*–/–*^ zebrafish likely experience compromised endocardial integrity and increased intercellular gaps, but only a subset reaches a critical threshold in the size of these gaps at which the endocardium fully separates from the cardiac jelly.^36^ Interestingly, approximately 30% of the current study mutants showed transient pericardial edema beginning around 2 dpf that resolved by 5 dpf. We hypothesize that in these embryos, the dynamically forming endocardial gaps are sufficiently closed during subsequent cardiac development, preventing complete endocardial detachment.^23^

### BA Dilatation in *fbn3* Mutants Reflects the Aortic Pathology Seen in MFS

Interestingly, the *fbn3*^*–/–*^ zebrafish without a pericardial phenotype at 5 dpf developed a dilated BA phenotype during the larval stage that persisted into adulthood. This phenotype is particularly relevant as the BA is anatomically and functionally related to the aortic root and ascending aorta in humans,^24^^,^^25^ which is typically dilated in patients with MFS.^37^ The elastic outflow properties of the BA support its evolutionarily conserved “Windkessel” function.^38^ Reports of aortic dilation in patients with *FBN2* defects^6^^,^^7^^,^^39^ support functional overlap among fibrillins and thus underscore the potential clinical relevance of this zebrafish model.

BA dilation is also characteristic of zebrafish mutants deficient in various mediators of TGF-β signaling, such as *alk5a*^*–/–*^; *alk5b*^*–/–*^^40^ and *ltbp1*^*–/–*^; *ltbp3*^*–/–*^^41^ double mutants; these mutants develop severe BA dilation, endocardial defects, and early lethality, phenotypes resembling the severely affected *fbn3*^*–/–*^ larvae. These similarities highlight the relevance of fibrillin and TGF-β interactions in vascular development and MFS pathogenesis.^41^

Unlike MFS mouse models, which exhibit elastin fragmentation, microdissections, and fatal aortic rupture,^42^ we found no evidence that BA dilation in *fbn3*^*–/–*^ zebrafish progresses to elastin breaks, dissection, or rupture. This discrepancy likely reflects species-specific structural features. The zebrafish outflow tract is considerably smaller and contains far fewer elastic lamellae than mammalian vessels, and it experiences very low blood pressure,^43^ which greatly reduces aortic wall stress. As a consequence, small structural defects may not present as stable, intermediate “pre-dissection” lesions in zebrafish and may instead progress directly to a full dissection but only under severe pathologic conditions. The only zebrafish line so far known to develop progressive ventral aortic dissection and rupture is a model with defects in the TGF-β effector proteins Smad3 and Smad6.^44^ Interestingly, even in that model of severe aortic disease, elastin breaks were not observed in the non-dissected regions. Thus, although BA dilation is clearly present, the vascular pathology in our *fbn3*^*–/–*^ model appears mild and did not reach the threshold required for dissection.

### *fbn3* Deficiency Induces Elevated Heart Rate and Arrhythmias

A slightly elevated heart rate was also found in the *fbn3*^*–/–*^ embryos of the current study. Although heart rate differences were not reported in common MFS mouse models,^45^ human-induced pluripotent stem cell–derived cardiomyocytes carrying a pathogenic *FBN1* variant also display an increased intrinsic beating rate.^46^ Of note, human cardiac electrophysiology compares more closely to zebrafish than to mice.^47^^,^^48^ In patients with MFS, cardiac arrhythmias are well-recognized clinical features,^49^ which have been replicated in mouse models.^50^ The adult *fbn3*^*–/–*^ zebrafish in the current study exhibited a higher frequency of irregular or skipped heartbeats, which might have important translational value given the clinical relevance of cardiac arrhythmias in MFS.^50^, ^51^, ^52^, ^53^

### Role of *fbn3* in Endothelial Function

Similar to the *pfd*^*gw1*^ mutant,^23^ the posterior caudal vein in *fbn3*^*–/–*^ zebrafish in the current study developed as a dilated structure with compromised vessel integrity and reduced endothelial barrier function during early development, shown by the leakage of both erythrocytes and intravenously injected fluorescent dye. This phenotype resolved in mutants that do not develop persistent endocardial detachment, but caudal vein dilation persisted when cardiac contraction and blood flow were pharmacologically inhibited. This suggests that fibrillin is required for blood flow–dependent biomechanical signaling crucial for vascular development, aligning with its established role in mechanosensing.^53^^,^^54^

In MFS mouse models (*Fbn1*^*C1041G/+*^ and *Fbn1*^*G234D/G234D*^), endothelial cells exhibit morphologic defects, including loss of alignment to blood flow, which are most pronounced at aneurysm-prone regions exposed to elevated mechanical stress.^55^^,^^56^ Because vascular smooth muscle cells are not recruited to the zebrafish arterial wall before 3 dpf, the early vascular defects observed here likely arise from endothelial dysfunction.^57^ The endocardial and venous phenotypes highlight an important role of fibrillins in endothelial structure and function, supported by previous evidence of endothelial dysfunction in patients with MFS.^58^^,^^59^

### Total Fibrillin Deficiency in Zebrafish Highlights Redundancy Between Different Fibrillins

In the current study, TKO zebrafish, lacking any functional fibrillin gene, displayed cardiovascular phenotypes during early development that are similar to the single *fbn3* KO, along with decreased general fitness and slightly decreased survival during larval stages. Interestingly, TKO zebrafish do not survive to adulthood, suggesting that the additional loss of *fbn3* unmasked essential functions of *fbn1* and *fbn2*. This also implies that loss of only 1 or 2 zebrafish fibrillins can generally be compensated by the other fibrillin(s) to guarantee (at least partial) survival, whereas the essential role of *fbn3* in cardiovascular homeostasis cannot be compensated. In mice, *Fbn1* was previously shown to functionally compensate for *Fbn2* loss during embryogenesis in a dosage-sensitive manner, confirming some level of redundancy in mammalian models as well.^60^

### *fbn3* Deficiency Triggers the Complement Pathway

Bulk RNA sequencing of our *fbn3* mutants revealed early up-regulation of several complement pathway components. The complement system, a key component of innate immunity, can be activated via the classical, lectin, or alternative pathways, and increasing evidence implicates the alternative pathway in the pathogenesis of cardiovascular diseases, including aortic aneurysms. For example, alternative pathway activation contributes to elastase-induced abdominal aortic aneurysm formation, and patients with TAAD exhibit elevated plasma levels of C3a and C5a in patients with TAAD. Genetic ablation of *Cfb* in *Fbn1*^*C1041/G+*^ MFS mice reduced TAAD incidence, and the C3a-C3aR axis contributes to TAAD via MMP2 up-regulation.^61^^,^^62^ C1R variants have also been associated with TAA in patients with bicuspid aortic valve, suggesting a broader involvement of the complement system in aortic disease.^63^

Collectively, these findings and the current study data imply that complement activation may play a role in the cardiovascular manifestations of MFS. As highlighted earlier, the current study’s *fbn3*^*–/–*^ zebrafish as well as MFS mouse models show clear evidence of endothelial dysfunction. We propose that this may serve as a trigger for complement activation, leading to the recruitment of inflammatory cells, including neutrophils, which we confirmed by whole-mount immunostaining and live-imaging in *fbn3* crispants. Neutrophils secrete MMP9, and to a lesser extent MMP13, which degrade key extracellular matrix collagens, promoting TAAD progression. Interestingly, *mmp9*, *mmp13a*, and *mmp13b* were increased in the *fbn3*^*–/–*^ RNA sequencing data set and could represent downstream targets of immune cell activation, contributing to aortic wall damage. Targeting the complement cascade, particularly components of the alternative pathway, may therefore represent a promising therapeutic strategy for preventing or mitigating TAAD in MFS.

### Cardiac Valve Abnormalities in Adult *fbn3* Mutants: Parallels With Patients With MFS

Elastin staining and synchrotron imaging revealed BV valve abnormalities in all adult *fbn3*^*–/–*^ zebrafish and AV valve defects in a smaller subset. Together with the increased inflow and outflow areas observed by color flow Doppler, these findings suggest an enlarged valvular annulus that could increase cross-sectional flow area despite preserved global cardiac performance. Nevertheless, alternative explanations, such as altered cardiac chamber compliance or subtle flow disturbances, cannot be excluded. More sensitive imaging modalities (eg, high–frame-rate echocardiography, 3D in vivo imaging) would be needed to resolve this, but such tools are not yet widely applied in zebrafish.

Similar valve defects are found in zebrafish lacking elastin a (*elna*^*sa12235*^),^64^ suggesting shared etiology and supporting the role of fibrillin as a scaffold for elastin. It is also plausible that the impaired endocardial cell behavior that we and others observed during early development in *fbn3*^*–/–*^zebrafish^23^ contributes to the valve defects seen in adults, as endocardial migration is crucial for early AV valve formation.^65^ Valve pathology has also been reported in *Fbn1*^*C1041G/+*^ and *Fbn1*^*C1041G/C1041G*^ MFS mouse models, both of which display dramatic alterations in mitral valve structure, including leaflet tip folding,^66^^,^^67^ features reminiscent of abnormalities in *fbn3*^*–/–*^ zebrafish with an AV valve phenotype.

The valve defects identified in the current *fbn3*^*–/–*^zebrafish model further support its validity as a model of the cardiovascular manifestations of MFS. In patients, mitral and tricuspid valve prolapse occurs in 65% and 35% of cases,^68^ although only 40% display associated regurgitation. In the current study’s zebrafish, regurgitation was detected only occasionally by pulsed wave Doppler, likely because mild or intermittent dysfunction may evade echocardiographic detection in such small hearts.^69^

Although some patients with MFS develop intrinsic cardiomyopathy, ventricular dysfunction is generally mild.^70^, ^71^, ^72^, ^73^ Consistent with this, the *fbn3*^*–/–*^ zebrafish displayed only mild ventricular hypertrophy with no functional abnormalities. Synchrotron-based 3D reconstructions also revealed no significant differences in cardiac chamber or BA dimensions. However, these measurements may be affected by postmortem tissue collapse, which could be addressed in future studies using vascular corrosion casting.^74^^,^^75^ Interestingly, *fbn3*^*–/–*^ zebrafish displayed higher variability in atrial volumes. Mutants with the largest atria also exhibited pronounced AV valve thickening, suggesting a pathophysiological consequence of prolonged volume overload from intermittent regurgitation.^76^ However, these cases were few, and echocardiography data were not available for direct confirmation.

### Study Limitations

An inevitable limitation of the current study concerns the interspecies relationship between the fibrillin genes in human and zebrafish. There is no definitive explanation why loss of zebrafish *fbn3*, rather than *fbn1*, gives rise to the observed MFS-like phenotypes, whereas *FBN1* is clearly the primary disease-causing gene in humans. Our data indicate that *fbn3* is more important during early development, resulting in the observed phenotypes in larvae, which persist until adulthood. The lack of any (progressive) phenotype in zebrafish lacking *fbn1* suggests a compensation by the other fibrillin(s), which is supported by the lack of viability of zebrafish lacking all fibrillin genes. Further studies will be necessary, however, to completely unravel the functional distinctions between the different zebrafish fibrillins. Nevertheless, our data are concordant in showing that loss of *fbn3* leads to phenotypes that mimic multiple cardiovascular manifestations of MFS.

The limitation of our *fbn3*^*–/–*^ zebrafish model is the observed phenotypic heterogeneity during larval stages. A conclusive explanation of the variable outcome of the endocardial phenotype in *fbn3*^*–/–*^ larvae, with only a subset developing a lethal phenotype, remains lacking. Our hypothesis that a stochastic process is the basis of the fate of the potential progression to complete endocardial detachment can only be proven by the exclusion of other possible explanations. Finally, the study focused predominantly on cardiovascular manifestations in this zebrafish model, as they are the chief cause of morbidity and mortality in patients with MFS. Additional studies will be necessary to investigate skeletal and ocular manifestations, which are common features in patients with MFS.

## Conclusions

This comprehensive study of zebrafish fibrillins found that disruption of zebrafish *fbn3* leads to a spectrum of cardiovascular abnormalities that resemble those observed in human MFS, including BA dilation, cardiac valve defects, and heart rhythm irregularities. These findings highlight the complex interspecies orthology of fibrillins. These data further suggest that the early phenotype observed in the current study model is potentially related to the activation of the innate immune system. The availability of this novel zebrafish model represents a significant new tool for research into the mechanisms of MFS-related cardiovascular manifestations and the identification of new therapeutic targets.

### Declaration of Generative AI and AI-Assisted Technologies in the Writing Process

The authors acknowledge the use of artificial intelligence tools (ChatGPT, Copilot, and Gemini) for text refinement.

Perspectives **COMPETENCY IN MEDICAL KNOWLEDGE:** MFS is a life-threatening connective tissue disorder with no effective cure. Understanding of its pathogenesis and available treatment options remains limited, highlighting the urgent need for more versatile animal models. We show that zebrafish with *fbn3* deficiency exhibit cardiovascular abnormalities that closely resemble those observed in patients with MFS. The data suggest that fibrillin defects disturb essential cardiovascular development processes, which is not addressed by currently recommended medical treatments for patients with MFS.

**TRANSLATIONAL OUTLOOK:** The availability of a zebrafish model that recapitulates multiple cardiovascular manifestations of MFS enables novel strategies to pursue better diagnosis and treatment options for patients. The cardiovascular phenotype of *fbn3*^*–/–*^ zebrafish larvae can serve as a valuable readout for unbiased in vivo high-throughput drug screening to uncover new therapeutic targets related to basic cardiovascular disease mechanisms involved in MFS. The model may also be of value to test the pathogenicity of rare *FBN1* variants identified with diagnostic testing.

## Funding Support and Author Disclosures

This work was supported by a grant from the Research Foundation Flanders (G0A8322N, Drs De Backer and Sips), the 2019 Grant for Medical Research from the Baillet Latour Fund (Dr De Backer), a Concerted Research Action grant from the Ghent University Special Research Fund (BOF GOA019-21, Drs Segers, De Backer, and Sips), and an Interdisciplinary Research grant of the Ghent University Special Research Fund (IOP-038-18, Drs Segers, De Backer, and Sips).

The authors have reported that they have no relationships relevant to the contents of this paper to disclose.

## Appendix

Video 1 BDM-Treated 2 dpf *Tg(kdrl:GFP)* Wild-Type Zebrafish Fluorescent imaging of the caudal vein of *Tg(kdrl:GFP)* wild-type zebrafish at 24 hours’ postfertilization after exposure to 10 mM BDM to inhibit cardiac contraction.

Video 2 Control 2 dpf *Tg(kdrl:GFP)* Wild-Type Zebrafish Fluorescent imaging of the caudal vein of *Tg(kdrl:GFP)* wild-type zebrafish at 24 hours’ postfertilization after exposure to 1% dimethyl sulfoxide as a solvent control.

Video 3 BDM-Treated 2 dpf *Tg(kdrl:GFP) fbn3*^*–/–*^ Zebrafish Fluorescent imaging of the caudal vein of *Tg(kdrl:GFP)* mild *fbn3*^*–/–*^ zebrafish at 24 hours’ postfertilization after exposure to 10 mM BDM to inhibit cardiac contraction.

Video 4 Control 2 dpf *Tg(kdrl:GFP) fbn3*^*–/–*^ Zebrafish Fluorescent imaging of the caudal vein of *Tg(kdrl:GFP) fbn3*^*–/–*^ zebrafish at 24 hours’ postfertilization after exposure to 1% dimethyl sulfoxide as a solvent control.

Video 5 FITC-Dextran–Injected Wild-Type Zebrafish at 1 hpi The caudal vasculature of 500 kDa FITC-dextran–injected 30 hours’ postfertilization *Tg(globin:GFP)* wild-type zebrafish, imaged at 1 hpi to assess vascular integrity.

Video 6 FITC-Dextran–Injected *fbn3*^*–/–*^ Zebrafish at 1 hpi The caudal vasculature of 500 kDa FITC-dextran–injected 30 hours’ postfertilization *Tg(globin:GFP) fbn3*^*–/–*^ zebrafish, imaged at 1 hpi to assess vascular integrity.

Video 7 FITC-Dextran–Injected Wild-Type Zebrafish at 20 hpi The caudal vasculature of 500 kDa FITC-dextran–injected 30 hours’ postfertilization *Tg(globin:GFP)* wild-type zebrafish, imaged at 20 hpi to assess vascular integrity.

Video 8 FITC-Dextran–Injected *fbn3*^*–/–*^ Zebrafish at 20 hpi The caudal vasculature of 500 kDa FITC-dextran–injected 30 hours’ postfertilization *Tg(globin:GFP) fbn3*^*–/–*^ zebrafish, imaged at 20 hpi to assess vascular integrity.

Video 9 *Tg(globin:GFP)* Wild-Type Zebrafish Heart at 3 dpf Fluorescent imaging of *Tg(globin:GFP)* wild-type zebrafish heart at 3 dpf. Video 10 *Tg(globin:GFP) fbn3*^*–/–*^ Zebrafish Heart at 3 dpf Fluorescent imaging of *Tg(globin:GFP)* wild-type zebrafish heart at 3 dpf, showing leakage of erythrocytes into the pericardial cavity.

Video 11 *Tg(globin:GFP)* Wild-Type Zebrafish Tail at 3 dpf Fluorescent imaging of *Tg(globin:GFP)* wild-type zebrafish tail at 3 dpf.

Video 12 *Tg(globin:GFP) fbn3*^*–/–*^ Zebrafish Tail at 3 dpf Fluorescent imaging of *Tg(globin:GFP)* wild-type zebrafish tail region at 3 dpf, showing leakage of erythrocytes.

Video 13 Live Imaging of Neutrophils in Control Zebrafish at 2 dpf Time-lapse imaging (z-stack acquired every 30 seconds for 2 hours) of neutrophil behavior in the tail region of a wild-type *Tg(mpx:GFP)* zebrafish embryo, in which neutrophils are fluorescently labeled in green. The tip of the tail is positioned on the right side of the frame. At 2 dpf, neutrophils in wild-type embryos are observed almost exclusively within the caudal hematopoietic tissue, displaying limited migration outside this region.

Video 14 Live Imaging of Neutrophils in F0 *fbn3*^*–/–*^ Zebrafish at 2 dpf Time-lapse imaging (z-stack acquired every 30 seconds for 2 hours) of neutrophil migration in the tail region of F0 *fbn3*^*–/–*^ crispant *Tg(mpx:GFP)* embryos. The tip of the tail is positioned on the right side of the frame. *fbn3*^*–/–*^ crispants display an increased number of neutrophils, especially located outside the caudal hematopoietic tissue toward the dorsal periphery. These neutrophils show increased migratory activity and patroling-like behavior throughout the tail region.

Video 15 Live Imaging of Neutrophils in Control Zebrafish at 3 dpf Time-lapse imaging (z-stack acquired every 30 seconds for 2 hours) of neutrophil behavior in the tail region of a wild-type *Tg(mpx:GFP)* zebrafish embryo, in which neutrophils are fluorescently labeled in green. The tip of the tail is positioned on the right side of the frame. At 3 dpf; neutrophils in wild-type embryos are observed almost exclusively within the caudal hematopoietic tissue, displaying limited migration outside this region.

Video 16 Live Imaging of Neutrophils in F0 *fbn3*^*–/–*^ Zebrafish at 3 dpf Time-lapse imaging (z-stack acquired every 30 seconds for 2 hours) of neutrophil migration in the tail region of F0 *fbn3*^*–/–*^ crispant *Tg(mpx:GFP)* embryos. The tip of the tail is positioned on the right side of the frame. *fbn3*^*–/–*^ crispants display an increased number of neutrophils, especially located outside the caudal hematopoietic tissue toward the dorsal periphery. These neutrophils show increased migratory activity and patroling-like behavior throughout the tail region.
