# Supplementary material for: Systematic Disruption of Zebrafish Fibrillin Genes Identifies a Translational Zebrafish Model for Marfan Syndrome
Source: JACC Basic Transl Sci. 2026 Apr 22;11(5):101543. doi: 10.1016/j.jacbts.2026.101543 (PMC13125888; doi:10.1016/j.jacbts.2026.101543)
Supplement: Supplementary Material [file mmc16.docx]

**Supplemental Appendix**

Supplemental Methods

Supplemental Table 1.

Supplemental Table 2.

Supplemental Table 3.

Supplemental Table 4.

Supplemental Table 5.

Supplemental Table 6.

Supplemental Table 7.

Supplemental Table 8.

Supplemental Figure 1.

Supplemental Figure 2.

Supplemental Figure 3.

Supplemental Figure 4.

Supplemental Figure 5.

Supplemental Figure 6.

Supplemental Figure 7.

Supplemental Figure 8.

Supplemental Figure 9.

Supplemental Figure 10.

Supplemental Figure 11.

Supplemental Figure 12.

Supplemental Figure 13.

Supplemental References

# **Supplemental Methods**

**Zebrafish husbandry**

Zebrafish were maintained in a semi-closed recirculating housing system (ZebTec, Tecniplast, Buguggiatte, Italy) at a constant temperature (27–28 °C), pH (~7.5), conductivity (~550 mS), and a light/dark cycle (14 h/10 h). Fish were fed twice daily with dry food (Gemma Micro, Skretting, Stavanger, Norway) and once with Micro Artemia (Ocean Nutrition, Essen, Belgium). Breeding and the collecting of the embryos were performed according to previously described protocols.^1^ All experiments were performed on offspring from stable mutant lines derived from heterozygotes outcrossed for at least four generations to WT zebrafish to avoid confounding off-target effects. All experiments were approved by the Animal Ethics Committee of the Ghent University Faculty of Medicine and Health Sciences (ECD 17-75K, ECD 17-78 and ECD 19-16K) and conform to the guidelines from Directive 2010/63/EU of the European Parliament on the protection of animals used for scientific purposes. All efforts were made to minimize pain, distress, and discomfort.

**Generation of fibrillin mutant zebrafish lines**

Single Guide RNA (sgRNA) sequences used for the generation of the various mutant fibrillin lines are summarized in Supplemental Table 1. gBlocks containing the entire sgRNA sequence preceded by a T7 promotor were obtained from Integrated DNA Technologies (Leuven, Belgium), and used as template for in vitro transcription using the MEGAshortscript™ Kit (Thermo Fisher Scientific, Waltham MA, USA) according to the manufacturer’s instructions. Purified sgRNA transcripts were then injected together with Cas9 protein (Toolgen, Seoul, Republic of Korea) as a ribonucleoprotein complex into fertilised eggs of zebrafish carrying the *Tg(kdrl:EGFP) or Tg(mpx:GFP)*  reporter at the single-cell stage using a Femtojet 4i microinjector (Eppendorf, USA). Embryos were maintained in an incubator (28 °C) in E3 medium until 5 dpf prior to transfer into the zebrafish facility. Genetically stable mutant zebrafish lines were established by backcrossing with wild type (WT, AB background) zebrafish.

The genotype of zebrafish was determined by finclipping the tail at 3 months post fertilization (mpf). Fish were shortly anesthetized using 1x tricaine methanesulfonate solution. Briefly, DNA was extracted by heating the caudal fin biopsy in 100 ml 50 mM sodium hydroxide (NaOH) at 95 °C for 20 minutes, followed by the addition of 10 ml 1 M Tris-HCl (pH 8) to neutralize the solution before this was added to the PCR reaction mix. The primer pairs used for PCR amplification and Sanger sequencing are summarized in Supplemental Table 2. Thermocycling conditions were 95 °C for 3 minutes, followed by 35 cycles of 95 °C for 15 seconds, 58 °C for 10 seconds, and 72 °C for 15 seconds, and a final extension of 72 °C for 10 minutes. Finally, Sanger sequencing was performed on amplified fragments, using the same primer set as for the PCR amplification.

**RNA extraction of whole embryos for RNAseq and RT-qPCR**

Total RNA of 10 pooled WT, heterozygous and/or homozygous mutant *fbn3* zebrafish, collected at specific timepoints as mentioned in the corresponding figure legends, was extracted using Trizol^ã^ (Life Technologies Europe). Subsequently, RNA was purified using the RNeasy Mini Kit (Qiagen, Hilden, Germany) in combination with on-column DNAse I treatment (Qiagen, Hilden, Germany) according to the manufacturer’s guidelines. RNA concentration and purity were quantified using the Little Lunatic (Unchained Labs, Pleasanton, CA, USA) and RNA quality was determined using Tapestation (Agilent, USA) according to the manufacturer’s guidelines. Only samples with RNA integrity number (RIN) values higher than 9 were considered. Next-generation sequencing libraries were prepared using the TruSeq Stranded Total RNA kit (Illumina, USA) following the manufacturer’s recommended procedures. Sequencing was then performed on an a NovaSeq6000 instrument (Illumina, USA). Following quality control,^2^ raw reads were aligned to the reference genome GRCz11 using STAR.^3^ Data normalization and filtering were conducted using standard workflows in R.^4–6^ Differential expression analysis was performed with edgeR, applying corrections for multiple testing.^7,8^ Multiplicity correction was performed by applying the Benjamini-Hochberg method to the p-values to control the false discovery rate (FDR). Differentially expressed genes (DEGs) were defined using a FDR-adjusted p-value < 0.05 and absolute log_2_ fold change > 1. GO enrichment analysis was subsequently performed to identify affected biological pathways.^9^

cDNA was synthesized starting from 750 ng RNA using the iScript cDNA Synthesis Kit (Bio-Rad Laboratories, Hercules, CA, USA), and primers (IDT; Integrated DNA Technologies) were designed using IDT’s PrimerQuest tool. For each cDNA sample, RT-qPCR reactions were prepared in triplicate with the addition of SsoAdvanced^™^ Universal SYBR^®^ Green Supermix (Bio-Rad Laboratories, Hercules, CA, USA) prior to analysis on a Roche LightCycler 480 System (Roche Diagnostics, Mannheim, Germany). The reference genes *elfa* and *bactin2* were used for normalization, which primer pairs are shown in Supplemental Table 3.^10^ Cq values were analysed using Excel using the ΔΔCq method.

**RNA extraction of isolated embryonic hearts for RT-qPCR**

RNA extraction was performed using a protocol partially based on the method described by Lombardo *et al*.^11^ Zebrafish hearts from 2 dpf (~50-100 hearts pooled per sample) and 5 dpf (~20-30 hearts pooled per sample) WT and *fbn1^-/-^* embryos were manually dissected in Tyrode’s solution (composition (in mM): NaCl 136, KCl 5.4, MgCl_2_ 1 mM, NaH_2_PO_4_ 0.3, CaCl_2_ 1.8, glucose 5, HEPES 10, BSA 1.5%, with pH adjusted to 7.4) to preserve their viability during harvesting as described previously.^12^ The pooled samples were stored in 750 µl RNA later before being centrifuged at 15,700 x g for 20 min at 4°C to pellet the hearts. The supernatant was carefully removed and checked under a stereomicroscope, without disturbing the pellet. Any remaining hearts in the supernatants were recovered and washed with PBS, and added to the previously obtained pellet in the smallest volume possible. Sedimented hearts were resuspended in 500 µl Trizol on ice and then vortexed to disrupt the tissue and incubated for 5 min at room temperature. Chloroform (100 µl) was then added and the homogenate was transferred to a pre-spun PLG tube followed by a 3 min incubation period at RT. Phase separation was achieved by centrifugation at 15,700 x g for 15 min at 4°C, after which the aqueous phase was transferred into a new tube. Subsequently, RNA was purified using the RNeasy Micro Kit (Qiagen, Hilden, Germany) in combination with on-column DNAse I treatment according to the manufacturer’s guidelines. RNA concentration and purity were quantified using the Little Lunatic, as previously described.

cDNA was synthesized starting from 50 ng RNA using the SuperScript^™^ VILO^™^ cDNA Synthesis Kit, according to the manufacturer’s guidelines. For each cDNA sample, RT-qPCR reactions were prepared in duplicate with the addition of SsoAdvanced^™^ Universal SYBR^®^ Green Supermix (Bio-Rad Laboratories, Hercules, CA, USA) prior to analysis on a Roche LightCycler 480 System (Roche Diagnostics, Mannheim, Germany). The reference genes *elfa* and *tdr7* were used for normalization, which primer pairs are shown in Supplemental Table 3.^10^ Cq values were analysed using Excel using the ΔΔCq method. Amplification curves were detected for all replicate measurements for *fbn3* and for both reference genes. Samples that did not yield a detectable amplification curve were classified as non-detects and assigned a Cq value of 40 (the assay’s maximum cycle number) for downstream analysis of this gene. This single-value imputation approach is commonly used in RT-qPCR studies to enable comparison across samples with low-abundance transcripts. Sample non-detects were present for one replicate of a 2 dpf *fbn1^-/-^* sample for *fbn2* detection, for one replicate of a 5 dpf WT sample for *fbn1* detection, for one replicate each of three 5 dpf *fbn1^-/-^* samples for *fbn1* detection, and for both replicates of an additional 5 dpf *fbn1^-/-^* sample for *fbn1* detection.

***In vivo* imaging of transgenic zebrafish larvae**

Live WT and mutant zebrafish larvae of various ages were anesthetized and embedded in a glass-bottom WillCo-dish (Willco Wells, Amsterdam, Netherlands) using 1% SeaPlaque low melting temperature agarose (Lonza, Basel, Switzerland) dissolved in E3 embryo medium with 0.5x tricaine. The embryos were either positioned with their heart directed towards the bottom of the dish to ensure optimal cardiac and ventral aorta visualisation or sideways positioned for optimal visualisation of the caudal vasculature. Images were captured using an inverted widefield microscope (Zeiss Axio Observer Z1), equipped with a Zeiss Axiocam 503 colour top mounted camera and operated by Zeiss ZenPro software.

To obtain a single in-focus brightfield image of the whole zebrafish larvae, z-stacks of 4 adjacent tiles were first acquired and merged along the optical axis using the Extended Depth of Focus (EDF) plugin in ImageJ software v.1.54g (National Institutes of Health, USA).^13^ The built-in stitching plugin of ImageJ was then used to merge the 4 individual focused tiles along the x-axis. Fluorescent images were acquired with the same inverted widefield microscope.

Brightfield imaging was used to evaluate the cardiovascular function of 3 dpf WT and *fbn3^-/-^* larvae, as previously described.^14^ Briefly, short recordings (~ 4-5 cardiac cycles) with 60 frames/sec were recorded of larvae in a lateral position. The ventricular area of end-diastole and end-systole from three consecutive cardiac cycles was then used to quantify different parameters: heart rate (bpm), stroke volume (nl), cardiac output (nl/min), ejection fraction (%), systolic volume (nl) and diastolic volume (nl). Measurements were made with ImageJ analysis software.

For deep tissue imaging, WT and mutant larvae at different developmental stages were immobilized in low melting temperature agarose, as previously described. To minimize image distortion caused by the beating of the heart, zebrafish were given an overdose of tricaine just prior to acquisition. Imaging was performed on a Nikon A1R HD confocal/multiphoton system using a 20x air objective (Plan Apo VC 20x, NA 0.8, WD 1000 µm, MRD70200, Nikon). A tunable Mai Tai DeepSee Ti:Sapphire femtosecond laser (690 – 1040 nm, Spectra-Physics) set at 900 nm was used for two-photon excitation of GFP-labeled endothelial cells. Fluorescence emission was collected in epi-detection through a 525/50 nm band-pass filter (MHE57610) using a GaAsP non-descanned detector (A1-GNEI-2, Nikon). Images were acquired with a galvano scanner in unidirectional mode without line accumulation or averaging, at a scan speed of 1 frame per second. The pixel size was 1.23 µm, and z-stacks were acquired at 1.07 µm steps. A maximum-intensity projection was generated from approximately 170 optical sections using ImageJ (NIH). Image acquisition was performed using NIS-Elements software.

**Pharmacological manipulation with myosin inhibitor**

The heartbeat of WT and mutant *fbn3* larvae was stopped by adding 10 mM myosin inhibitor 2,3-butanedione 2-monoxime to E3 embryo medium. In parallel, control WT and mutant *fbn3* larvae were exposed to vehicle (1% dimethyl sulfoxide (DMSO). The compounds were added to the petri dish prior to the start of systemic blood circulation (± 22 hours post fertilization (hpf)), followed by manual dechorionation of the embryo. Embryos were embedded sideways in 1% low melting temperature agarose, as mentioned above. Z-stack imaging of the caudal vein of 24 and 48 hpf larvae was acquired using the same inverted widefield microscope (Zeiss Axio Observer Z1), equipped with a Zeiss Axiocam 503 colour top mounted camera and operated by Zeiss ZenPro software. ImageJ was used to perform caudal vein measurements.

**Whole-mount staining**

Immunofluorescence staining was adapted from a protocol previously described by Westerfield *et al.^1^* Euthanized 3 and 7 dpf embryos were fixed in 4% paraformaldehyde (PFA) in PBS for 2 hours at room temperature, rinsed in PBS and washed four times in PBS containing 0.8% Triton^TM^ X-100 (PBST). Embryos were then incubated in 100% methanol at -20°C for 30 min and subsequently rehydrated through a graded methanol/PBST series (75%, 50%, 25%). Permeabilization was performed using proteinase K with conditions adjusted to the developmental stage: 3 dpf embryos were treated with 20 µg/ml for 30 min, whereas 7 dpf embryos were treated with 30 µg/ml for 30 min. Enzyme activity was stopped by post-fixation in 4% PFA for 25 min at RT, after which samples were blocked in 5% bovine serum albumin in PBS for 1 hour at RT. Embryos were incubated overnight at 4°C with the primary antibody [Anti-Mpx (1:100, Genetex, GTX128379)] diluted in blocking solution, washed several times in PBST, and incubated overnight at 4°C with the fluorescent secondary antibody [donkey anti-rabbit Alexa Fluor^TM^ 594 (1:500, Invitrogen, A-21207)]. Finally, WT and *fbn3^-/-^* embryos at 3 and 7 dpf were embedded in 1% SeaPlaque low-melting agarose, as previously described.

Imaging was performed with a Nikon A1R HD Confocal microscope applying a 10x air (Plan Apo Lambda D 10X air objective, NA 0.45, WD 4.0, MRD70170, Nikon). A 488 nm and 561 nm diode laser (LU-N4 LASER UNIT 405/488/561/640, Nikon) was used for excitation of GFP-labelled endothelial cells and red-labelled neutrophils, respectively. A 405/488/561/640 dichroic mirror was used to separate the laser excitation light from the fluorescence signal. Fluorescence emission was detected through a 525/50nm (MHE57030) and 585/65 (MHE57070) band pass filter on GaAsP PMT’s (A1-DUG-2 GaAsP Multi Detector Unit, Nikon). In parallel, a brightfield image was acquired using the transmission detector. A galvano scanner was used for unidirectional scanning without accumulation or line averaging and a scan speed of 0.5 frames per second. The pinhole was set to 1.2 AU (19.16 µm) and the pixel size was 1.22 µm/pixel. A z-stack was acquired with a step size of 2.83 µm. NIS Elements was used as acquisition software. Confocal imaging was performed at the Ghent Light Microscopy (GLiM) CORE at Ghent University (Belgium).

Quantification of the whole-mount staining was performed in Fiji (ImageJ) based on thresholding. For each sample, a maximum-intensity projection was generated and the fluorescent signal was then isolated by applying a fixed intensity threshold. The region of interest was manually outlined and the number of neutrophils in the threshold limited area were measured.

**Assessment of vascular integrity by FITC-dextran injections**

WT and *fbn3^-/-^* zebrafish embryos aged ~ 30 hpf were embedded laterally in 0.5% SeaPlaque low melting temperature agarose, as previously described. Fluorescein isothiocyanate (FITC) - dextran (500 kDa) was injected into the duct of Cuvier, allowing the tracer to enter the heart and systemic circulation. The caudal vasculature was imaged at 1, 4, and 20 hours post-injection to assess vascular integrity, as determined by the presence of extravascular dye leakage.

**Transthoracic echocardiography of adult zebrafish**

Two-dimensional transthoracic echocardiography was performed using a Vevo 2100 ultrasound machine (Fujifilm VisualSonics, Toronto, Canada) and MS 700 linear array probe (50 MHz) (Fujifilm, VisualSonics, Toronto, Canada) according to the detailed protocol by Van Impe *et al*.^15^ Briefly, zebrafish were anesthetized in a 1x tricaine solution at 28 °C until a lack of response to external stimuli was observed. Next, zebrafish were stabilised in a customized 3D printed holder between two spongy clamps, submerged with 0.5x tricaine solution at 28 °C to maintain anaesthesia during the experiment, while minimizing the impact of anaesthesia on the cardiovascular parameters. All measurements were obtained within 3–4 minutes after the start of anaesthesia induction.

Pulsed-wave Doppler (PWD) and Colour Flow Doppler (CFD) ultrasound recordings were used for defining ventricular and bulbus arteriosus (BA) measurements *in vivo*, as well as quantifying dynamics of blood flow through the atrioventricular (AV) valve (ventricular inflow) and bulboventricular (BV) valve (ventricular outflow). PWD recordings were processed in a completely automated and unbiased manner in MATLAB v23.2.0 (R2023b) (MathWorks, Natick, MA), using previously published and validated algorithms.^15^ Overview of PWD ventricular inflow and outflow parameters is indicated in Supplemental Table 4.

The Matlab script also provided a visual overview of the heartbeat peaks during the 20-second-long recordings. To investigate beat-to-beat variability, therefore checking for heartbeat irregularity, we manually scored the heartbeat recordings. Score 1 to 3 was given to each sample, ranging from score 1 for recordings with no heartbeat variability, to score 3 for samples with highly irregular heartbeats.

Hemodynamic measurements of the ventricle and BA, as well as the analysis of the CFD ultrasound recordings, were manually performed using the Vevo LAB^TM^ analysis software package v5.8.2 (Fujifilm VisualSonics, Toronto, Canada). Tracings of the walls of the ventricle and BA were done in a 2D longitudinal axis view. Ventricular diastolic and systolic parameters are also indicated in Supplemental Table 4. To obtain the maximal dimensions of BA, its tracings were done immediately following BV valve contraction, revealing its maximal area, volume, and diameter.

CFD recordings in an abdominocranial axis view were used for directly visualising blood flow through the valves and possible regurgitation. Ventricular inflow and outflow areas were measured while in maximal intensity and averaged among three cardiac cycles. Regurgitation fractions of each were calculated as the ratios of inflow or outflow areas and their simultaneous retrograde flow.

WT and mutant zebrafish used for the echocardiography experiments ranged from 6 to 16 mpf but were age-matched for each experiment. To account for age- and sex-related differences in body sizes, ventricular and BA volumes were normalized to body surface area (recommended by Wang *et al*), which was calculated by the formula body surface area=8.46×weight^0.66^.^16^ The same was performed for PWD parameters atrial wave, atrial regurgitation, aortic wave, and aortic regurgitation, as recommended by Van Impe at al. As it was proven to have significant male-female differences, only zebrafish males were used for calculating the early peak and velocity time interval of the early peak. All of the analyses were done by operators blinded to the genotype.

**Histological staining of adult zebrafish**

Adult zebrafish were euthanized by lethal dose of tricaine (1 g/L) and fixed in 4% paraformaldehyde in phosphate-buffered saline solution for at least 24 h, then decalcified with citric acid (45% formic acid, 5% sodium citrate) for 4-6 h. The samples were stored in 70% ethanol until paraffin embedding and microtome cutting into 5 μm thick sections using a Leica TP1020 tissue processor. For staining for elastin, a combination of Weigert’s Hematoxylin and Resorcin-Fuchsin staining was used for distinguishing nuclei and elastic fibers, respectively, with Van Gieson’s counterstain to examine collagen and miscellaneous tissue elements.^17^ For staining for collagen, Picrosirius Red solution was used.^18^

**Synchrotron imaging and 3D reconstruction of zebrafish hearts**

Propagation-based phase-contrast synchrotron X-ray imaging was performed at the TOMCAT (X02DA) beamline of the Swiss Light Source (Paul Scherrer Institute, Villigen, Switzerland) and at the I13-2 beamline of Diamond Light Source (Harwell Science and Innovation Campus, Oxfordshire, UK). *fbn3^-/-^* zebrafish aged 16 and 18 mpf, along with age-matched WT controls, were fixed, decalcified, and stored in 70% ethanol. Directly before scanning, samples were immobilized vertically in 1.5 ml Eppendorf tubes using 1% low- melting temperature agarose. Custom-made sample holders were used to secure the Eppendorf tubes to the rotation stage between the X-ray source and CCD camera.

Image stacks with a resolution of 2560 × 2560 pixels and 2160 slices were captured by a photon detector mounted on a visual light microscope. Total magnification was 4×, with a field of view of 4.16 mm × 4.16 mm (in-plane) × 3.51 mm (axial), and an isotropic voxel size of 1.625 µm^3^. Tomographic reconstruction, including phase retrieval, was performed on-site using Paganin’s algorithm.^19^ Detailed scan parameters of the individual beamlines optimized for zebrafish samples^20^ are provided in the Supplemental Table 5.

Image processing was performed using the medical software package Mimics v25.0 (Materialise, Leuven, Belgium). The zebrafish hearts, including the atrium, ventricle, cardiac valves, and BA with the aorta until the first branching site, were semi-automatically segmented. 3D models of the zebrafish hearts were generated from these masks, enabling analysis of volumes or 2D dimensions, such as valve length and thickness or BA diameter, and their comparisons between the genotypes (*fbn3^-/-^* vs WT). Measurements were normalised to body surface area.

**Supplemental Table 1.** Sequence of sgRNA used for each genotype.

| **Gene** | **Name** | **Exon** | **Size INDEL** | **sgRNA sequence** | **Predicted**  **nucleotide change** | **Predicted protein change** |
| --- | --- | --- | --- | --- | --- | --- |
| *fbn1* | Cmg79 | 34 | 4bp deletion | GGCTGGACGAGTGCTCGAAC | c.4223_4226del | p.S1408fs |
|  | Cmg57 | 38 | 4bp deletion | GGCCTGGACGTCCAGTACCG | c.4737_4740del | p.Q1579fs |
|  | Cmg64 | 38 | 4bp insertion | GGAACAGCAGCAGGACGCTT | c.4801_4802insTGAT | p.A1601fs |
|  | Cmg80 | 2 | 16bp deletion | GGGTGGAAAACCCTGACCGG | c.259_274del | p.L87fs |
| *fbn2* | Cmg86 | 5 | 2bp deletion | TGCCACAGTGGGTCAAGCGT | c.581_582del | p.A194V |
|  | Cmg87 | 5 | 22bp deletion | TGCCACAGTGGGTCAAGCGT | c.558_599del | p.L186= |
|  | Cmg95 | 5 | 12bp deletion | TGCCACAGTGGGTCAAGCGT | c.579_590del | p.Q194H |
| *fbn3* | Cmg96 | 4 | 4bp deletion | TGCGAGAGCGGATGTCAGAA | c.458_461del | p.C153* |
|  | Cmg97 | 4 | 15bp deletion | TGCGAGAGCGGATGTCAGAA | c.462_476del | p.Q154R |

**Supplemental Table 2.** Primer pairs used for genotype verification.

| **Gene** | **Name** | **Exon** | **Size INDEL** | **Forward primer** | **Reverse primer** |
| --- | --- | --- | --- | --- | --- |
| *fbn1* | Cmg79 | 34 | 4bp deletion | ATTAACGAGTGTGAGATCGGG | GGCTTCTACTGCACTGGTAC |
|  | Cmg57 | 38 | 4bp deletion | AGCAATCATAAGCACGAGATCC | CCTGCTGCTGTTCTAGGG |
|  | Cmg64 | 38 | 4bp insertion | AGAATTGTATATGCAGGTCCAG | CCTGGAGGAGAAGGATTCAGA |
|  | Cmg80 | 2 | 16bp deletion | TTTATGAGTGTTTGACCCCTCC | GCTGGAGGAAACAGGATAAGT |
| *fbn2* | Cmg86 | 5 | 2 bp deletion | ATGTAAATCGCAGACTACCGGA | CCAAGGTCAGCACTGATTTTAT |
|  | Cmg87 | 5 | 22bp deletion | ATGTAAATCGCAGACTACCGGA | CCAAGGTCAGCACTGATTTTAT |
|  | Cmg95 | 5 | 12bp deletion | ATGTAAATCGCAGACTACCGGA | CCAAGGTCAGCACTGATTTTAT |
| *fbn3* | Cmg96 | 4 | 4bp deletion | TTCTGGGTCACACTGACACG | ACATTGCTTGCTTGGGATATTTGT |
|  | Cmg97 | 4 | 15bp deletion | TTCTGGGTCACACTGACACG | ACATTGCTTGCTTGGGATATTTGT |

**Supplemental Table 3.** Summary of primers used for RT-qPCR.

| **Reference target** | **Forward primer** | **Reverse primer** |
| --- | --- | --- |
| *fbn1* | GTTCAGGCTCCTAAACCCTGT | GACAGTTGTGCTGCTTGGTG |
| *fbn2* | GAGCAGTGCCCACCTATCAG | CACAGATCGGGGACAACCTT |
| *fbn3* | TGGTGGGAACATACCAGTGC | AGCTTCCCTCCGAGTTTGTG |
| *elfa* | *GGAGACTGGTGTCCTCAA* | *GGTGCATCTCAACAGACTT* |
| *bactin2* | *TGAGCTGAAACTTTACAGACACAT* | *AGACTTTGGTGTCTCCAGAATG* |
| *tdr7* | GCAGCATAATTTGAGTACACCC | TTGCCTATATTCACTGAGAAATGGA |

**Supplemental Table 4.** Overview of the cardiovascular parameters obtained by two-dimensional transthoracic echocardiography of

adult fibrillin-impaired zebrafish.

| **Abbreviation** | **Unit** | **Description** | **Method of parameter measurement** |
| --- | --- | --- | --- |
| **A peak** | **mm/s** | peak velocity of the blood flow through the atrioventricular valve during diastole (inflow) | automated PWD |
| **A VTI** | **mm** | velocity time integral of the blood flow through the atrioventricular valve during diastole (inflow) | automated PWD |
| **A regurg** | **mm/s** | peak value of the atrioventricular valve regurgitation | automated PWD |
| **E peak** | **mm/s** | peak velocity of the passive blood flow through the atrioventricular valve during early diastole (inflow) | automated PWD |
| **E VTI** | **mm** | velocity time integral of the passive blood flow through the atrioventricular valve during early diastole (inflow) | automated PWD |
| **AET** | **ms** | aortic ejection time, i.e. the duration of bulboventricular valve being opened | automated PWD |
| **NFT** | **ms** | no flow time, i.e. the duration of atrioventricular valve being closed | automated PWD |
| **HR** | **beat/min** | heart rate | automated PWD |
| **HR st dev** | **-** | standard deviation of heart rate values | automated PWD |
| **Ao peak** | **mm/s** | peak velocity of the blood flow through the bulboventricular valve during systole (outflow) | automated PWD |
| **Ao VTI** | **mm** | velocity time integral of the blood flow through the bulboventricular valve during systole (outflow) | automated PWD |
| **Ao regurg** | **mm/s** | peak value of the aortic valve regurgitation | automated PWD |
| **area;d** | **mm^2^** | ventricular area during diastole | ventricular tracing |
| **area;s** | **mm^2^** | ventricular area during systole | ventricular tracing |
| **CO** | **ml/min** | cardiac output | ventricular tracing |
| **EF** | **%** | ventricular blood ejection fraction | ventricular tracing |
| **FS** | **%** | ventricular fractional shortening | ventricular tracing |
| **SV** | **µl** | ventricular stroke volume | ventricular tracing |

| **volume;d** | **µl** | ventricular volume during diastole | ventricular tracing |
| --- | --- | --- | --- |
| **volume,s** | **µl** | ventricular volume during systole | ventricular tracing |
| **inflow area** | **mm^2^** | amount of blood (in 2D) flowing to the ventricle through the atrioventricular valve during diastole (inflow) | CFD |
| **outflow area** | **mm^2^** | amount of blood (in 2D) flowing to the bulbus arteriosus through the bulboventricular valve during systole (outflow) | CFD |
| **inflow regurg fraction** | **%** | relative amount of blood (in 2D) that returns to the atrium through the atrioventricular valve during diastole; inflow area/retrograde flow area | CFD |
| **outflow regurg fraction** | **%** | relative amount of blood (in 2D) that returns to the ventricle through the bulboventricular valve during systole; outflow area/retrograde flow area | CFD |

**Supplemental Table 5.** P-values of cardiovascular parameters obtained by two-dimensional transthoracic echocardiography of adult *fbn1* and *fbn1*, *fbn2a* double mutant zebrafish, compared to age-matched WT or fbn1 mutants, respectively.

|  | ***fbn1*** | | | | ***fbn1, fbn2*** | | | |
| --- | --- | --- | --- | --- | --- | --- | --- | --- |
|  | ***fbn1 cmg57*** | ***fbn1 cmg64*** | ***fbn1 cmg79*** | ***fbn1 cmg80*** | ***fbn1 cmg80, fbn2 cmg86*** | ***fbn1 cmg80, fbn2 cmg87*** | ***fbn1 cmg80, fbn2 cmg95*** | ***fbn1 cmg79, fbn2 cmg95*** |
| **A peak** | 0.31 | 0.88 | 0.58 | 0.020 | 0.63 | 0.026 | 0.22 | 0.57 |
| **A VTI** | 0.021 | 0.43 | 0.85 | 0.57 | 0.25 | 0.045 | 0.40 | 0.82 |
| **A regurg** | 0.23 | 0.46 | 0.56 | 0.082 | 0.96 | 0.72 | 0.86 | 0.057 |
| **E peak** | 0.85 | 0.20 | 0.67 | 0.071 | 0.39 | 0.083 | 0.98 | 0.30 |
| **E VTI** | 0.31 | 0.13 | 0.91 | 0.79 | 0.25 | 0.11 | 0.76 | 0.41 |
| **AET** | 0.96 | 0.93 | 0.89 | 0.30 | 0.69 | 0.40 | 0.42 | 0.087 |
| **NFT** | 0.13 | 0.85 | 0.21 | 0.33 | 0.70 | 0.31 | 0.27 | 0.47 |
| **HR** | 0.34 | 0.75 | 0.22 | 0.61 | 0.93 | 0.15 | 0.74 | 0.70 |
| **HR st dev** | 0.88 | 0.23 | 0.59 | 0.75 | 0.57 | 0.67 | 0.53 | 0.61 |
| **Ao peak** | 0.67 | 0.24 | 0.29 | 0.51 | 0.36 | 0.69 | 0.24 | 0.73 |
| **Ao VTI** | 0.52 | 0.055 | 0.46 | 0.96 | 0.74 | 0.46 | 0.95 | 0.51 |
| **Ao regurg** | 0.25 | 0.022 | 0.77 | 0.96 | 0.44 | 0.17 | 0.92 | 0.18 |
| **area;d** | 0.005 | 0.38 | 0.78 | 0.48 | 0.11 | 0.98 | 0.53 | 0.31 |
| **area;s** | 0.012 | 0.24 | 0.47 | 0.37 | 0.49 | 0.97 | 0.61 | 0.87 |
| **CO** | 0.033 | 0.41 | 0.95 | 0.24 | 0.035 | 0.94 | 0.50 | 0.030 |
| **EF** | 0.69 | 0.95 | 0.77 | 0.044 | 0.49 | 0.36 | 0.27 | 0.51 |
| **FS** | 0.41 | 0.16 | 0.63 | 0.63 | 0.72 | 0.92 | 0.081 | 0.35 |
| **SV** | 0.032 | 0.37 | 0.73 | 0.50 | 0.057 | 0.66 | 0.53 | 0.030 |
| **volume;d** | 0.001 (↑) ^a^ | 0.79 | 0.65 | 0.42 | 0.034 | 0.63 | 0.70 | 0.093 |
| **volume,s** | 0.007 | 0.61 | 0.49 | 0.20 | 0.20 | 0.75 | 0.94 | 0.62 |
| **inflow area** | 0.043 | 0.24 | 0.64 | 0.86 | 0.53 | 0.36 | 0.56 | 0.007 |
| **outflow area** | 0.17 | 0.024 | 0.86 | 0.066 | 0.29 | 0.53 | 0.69 | 0.075 |
| **inflow regurg fraction** | 0.50 | 0.40 | 0.78 | 0.62 | 0.94 | 0.46 | 0.000 | 0.85 |
| **outflow regurg fraction** | 0.50 | 0.81 | 0.65 | 0.94 | 0.76 | 0.48 | 0.14 | 0.28 |

No significant differences were observed in any cardiovascular parameters. Statistical analysis: Shapiro-Wilk normality testing, followed by an unpaired t-test was used for normally distributed data, while non-normally distributed data was analyzed using the Mann-Whitney *U* tests. Bonferroni-adjusted values are reported (significance threshold: P ≤ 0.002), and the direction of change is indicated by an arrow (↑higher or ↓lower). The abbreviations list and explanations of the cardiovascular parameters are included in Supplemental Table 1.

^a^ Higher ventricle volume of the *fbn1 cmg57* mutants has been attributed to be clutch-specific and should not be interpreted as a significant finding, especially when comparing it to the other *fbn1* mutant lines.

**Supplemental Table 6.** P-values of cardiovascular parameters obtained by two-dimensional transthoracic echocardiography of adult *fbn3* impaired zebrafish, compared to respective age-matched WT.

|  | ***fbn3*** | |
| --- | --- | --- |
|  | ***fbn3 cmg96*** | ***fbn3 cmg97*** |
| **A peak** | 0.16 | 0.093 |
| **A VTI** | 0.053 | 0.22 |
| **A regurg** | 0.43 | 0.17 |
| **E peak** | 0.007 | 0.48 |
| **E VTI** | >0.99 | 0.15 |
| **AET** | 0.45 | 0.30 |
| **NFT** | 0.93 | 0.036 |
| **HR** | 0.71 | 0.15 |
| **HR st dev** | 0.082 | 0.87 |
| **Ao peak** | 0.89 | 0.34 |
| **Ao VTI** | 0.51 | 0.88 |
| **Ao regurg** | 0.27 | 0.40 |
| **area;d** | 0.054 | 0.68 |
| **area;s** | 0.028 | 0.82 |
| **CO** | 0.98 | 0.44 |
| **EF** | 0.42 | 0.55 |
| **FS** | 0.83 | 0.41 |
| **SV** | 0.70 | 0.44 |
| **volume;d** | 0.076 | 0.54 |
| **volume,s** | 0.024 | 0.70 |
| **inflow area** | <0.001 (↑) | 0.53 |
| **outflow area** | 0.001 (↑) | 0.001 (↑) |
| **inflow regurg fraction** | 0.45 | 0.063 |
| **outflow regurg fraction** | 0.23 | 0.25 |

The *fbn3 cmg96* mutant line exhibits increased inflow and outflow areas, with higher outflow also observed for the *cmg97* line. These observations are in line with the increase in length and thickness of the valve leaflets revealed by synchrotron imaging. No significant differences were observed in any other cardiovascular parameters compared to WT. Statistical analysis: Shapiro-Wilk normality testing, followed by an unpaired t-test was used for normally distributed data, while non-normally distributed data was analyzed using the Mann-Whitney *U* tests. Bonferroni-adjusted values are reported (significance threshold: P ≤ 0.002), and the direction of change is indicated by an arrow (↑higher or ↓lower). The abbreviations list and explanations of the cardiovascular parameters are included in Supplemental Table 1.

**Supplemental Table 7.** Overview of scan parameters across two synchrotron facilities.

| **Synchrotron facility** | Swiss Light Source, Paul Scherrer Institute, Switzerland | Diamond Light Source, Harwell Science and Innovation Campus, UK |
| --- | --- | --- |
| **Beamline** | TOMCAT (X02DA) | I13-2 |
| **Beam energy** | 21.8 keV monochromatic beam | 27 keV pink beam |
| **Object-detector distance** | 250 mm | 495 mm (-415 mm) and 295 mm (-615 mm) |
| **Number of projections** | 1501 | 1501 |
| **Scintillator** | LUAg:CE 20 µm | LuAg 500 µm |
| **Objective** | UPLAPO 4× objective | PlanApoN 2× objective |
| **Camera** | PCO.Edge 5.5 | PCO.Edge 5.5 |

**Supplemental Table 8.** Transcriptomic analysis of *fbn3^-/-^* mutants.

(Tab 1 and 2) List of all differentially expressed genes (DEG) in *fbn3^-/-^* mutants to WT control siblings at 1 and 2 dpf, based on logFC and adjusted p-value (FDR). (Tab 3 and 4) Overview of all gene ontology-enriched biological pathways in *fbn3^-/-^* mutants to WT control siblings at 1 and 2 dpf. Supplemental Table 8 is attached as a separate file.

**
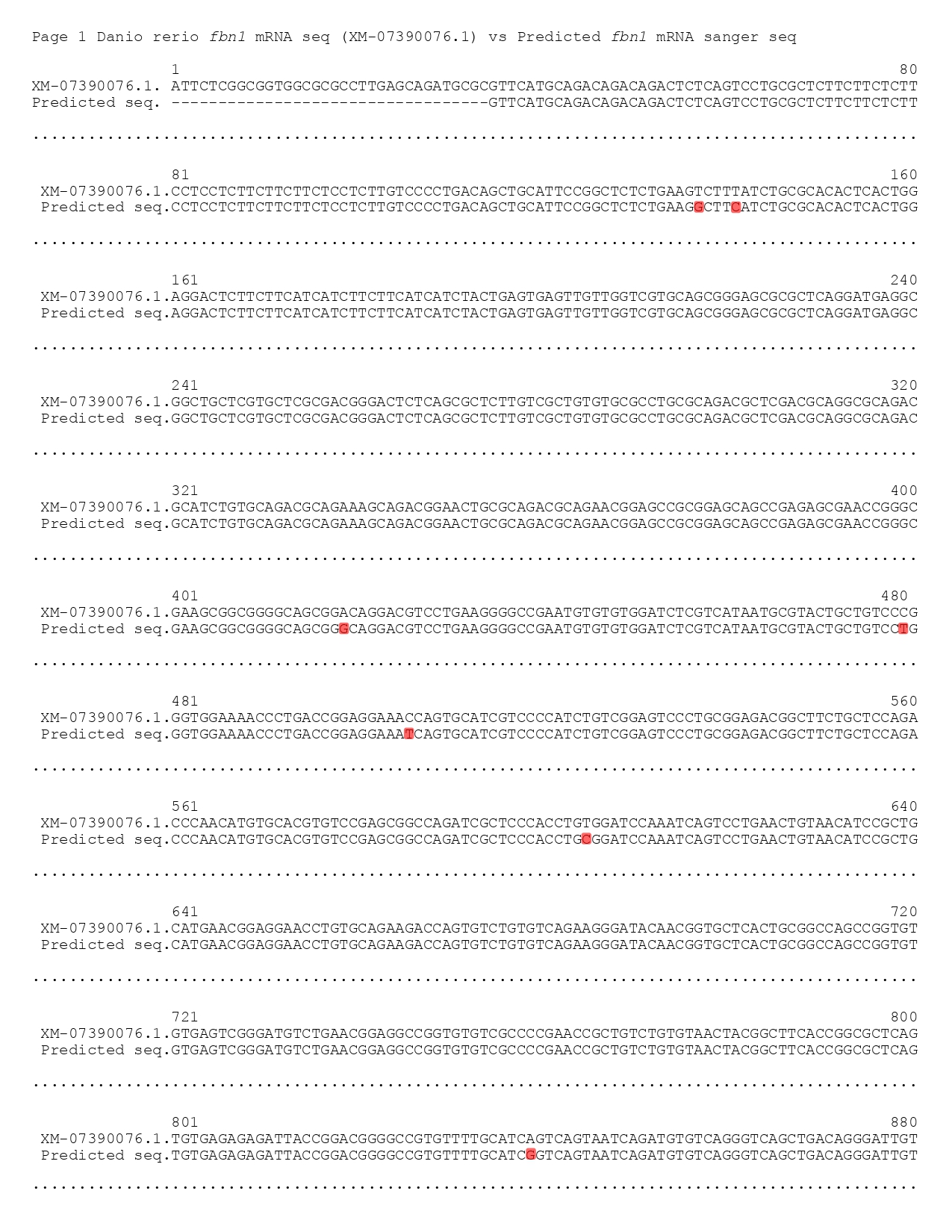
**

**
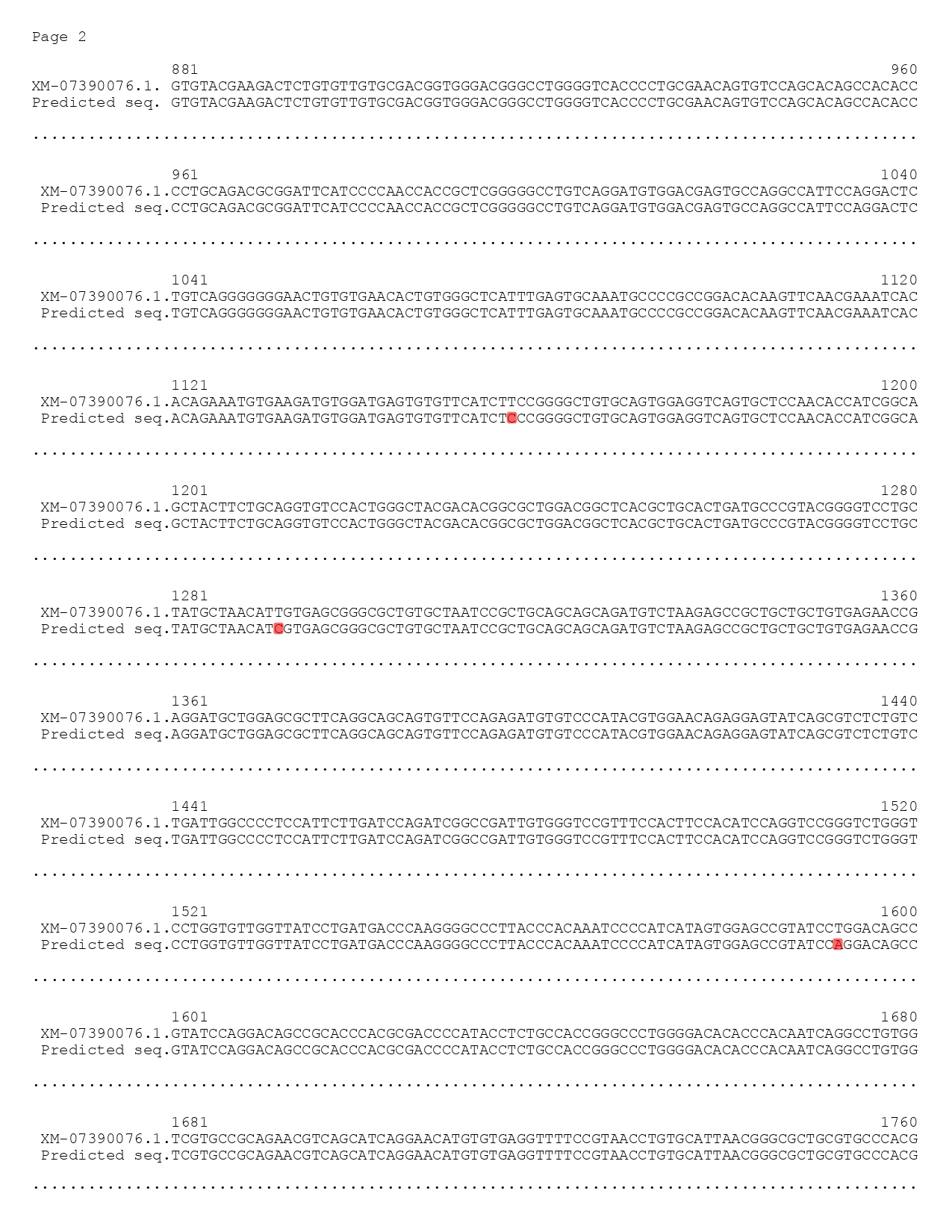
**

**
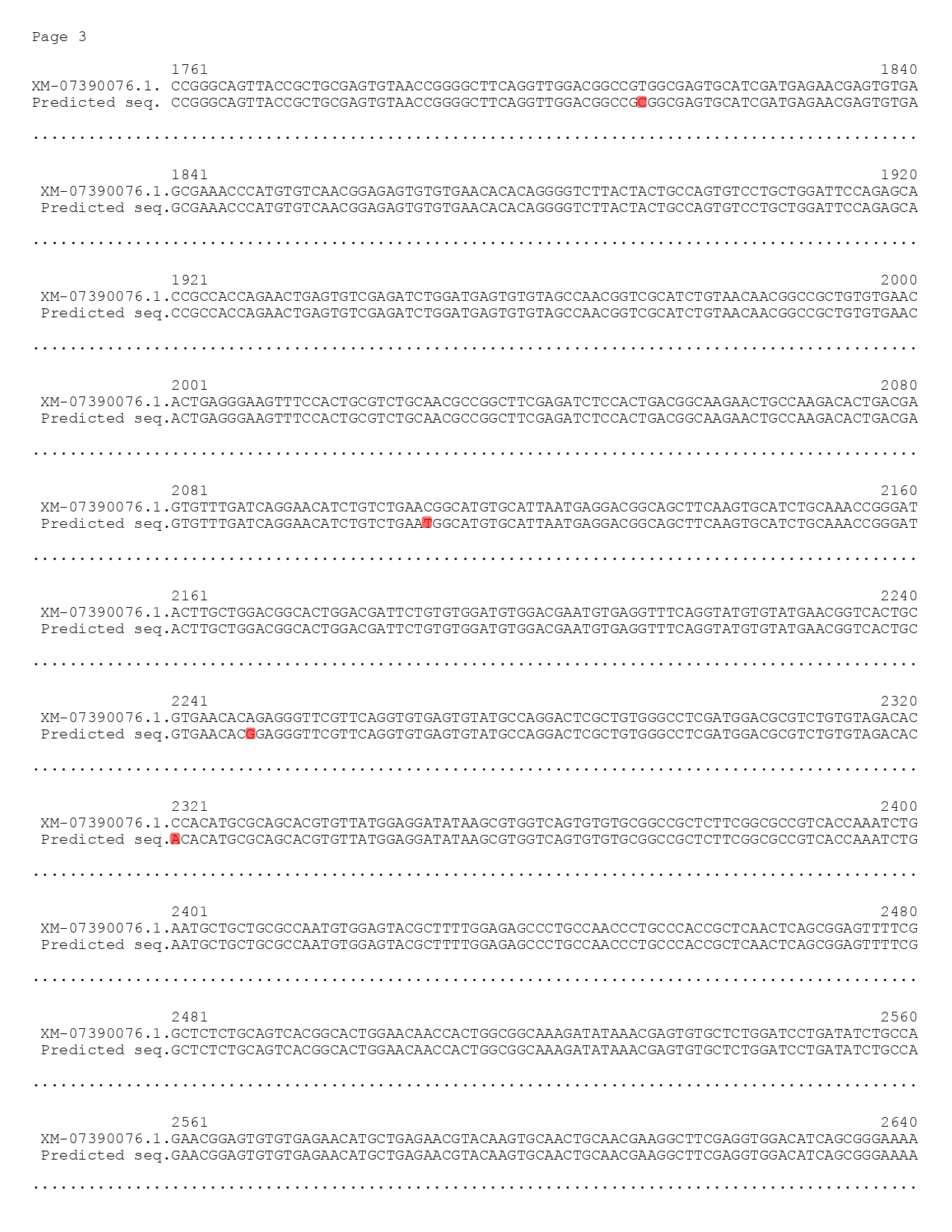
**

**
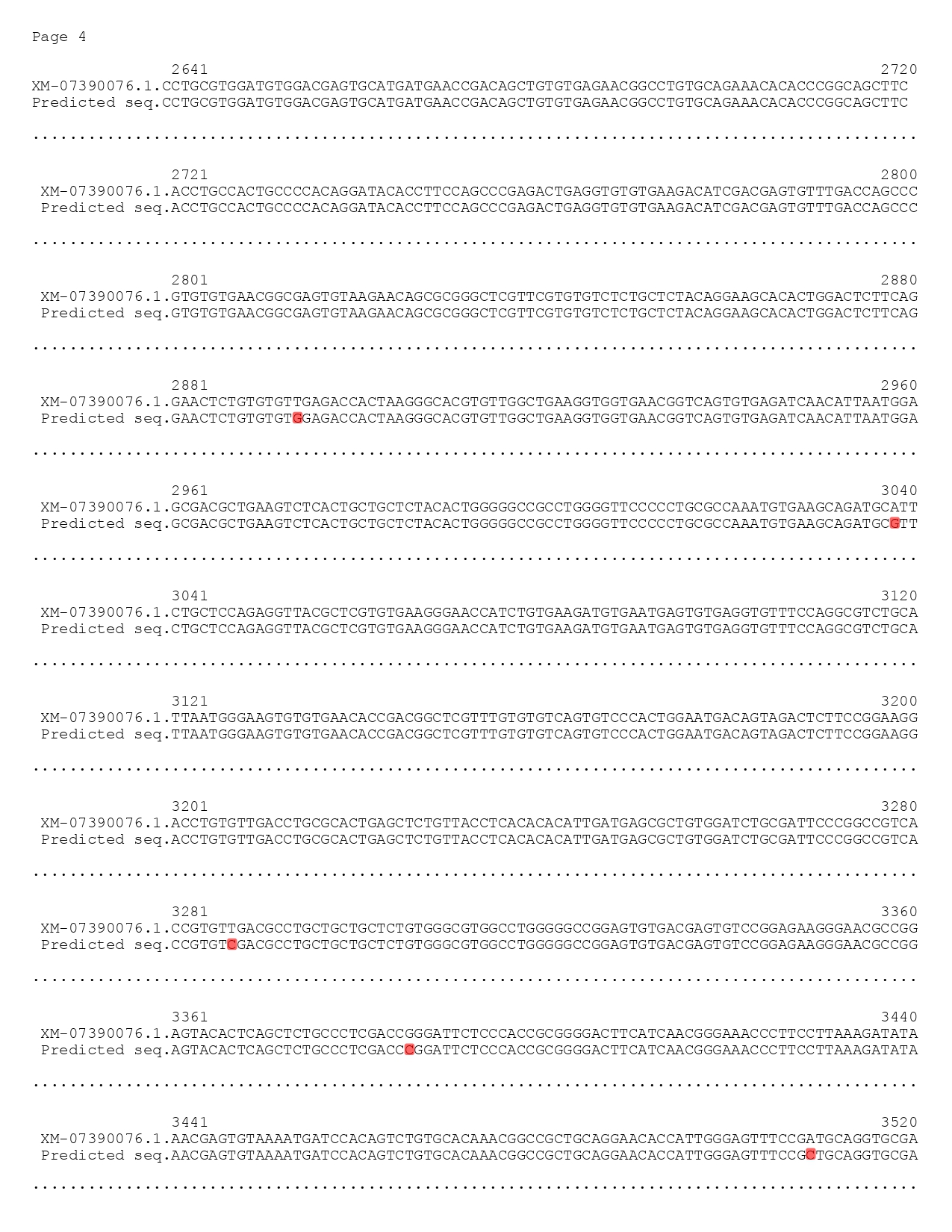
**

**
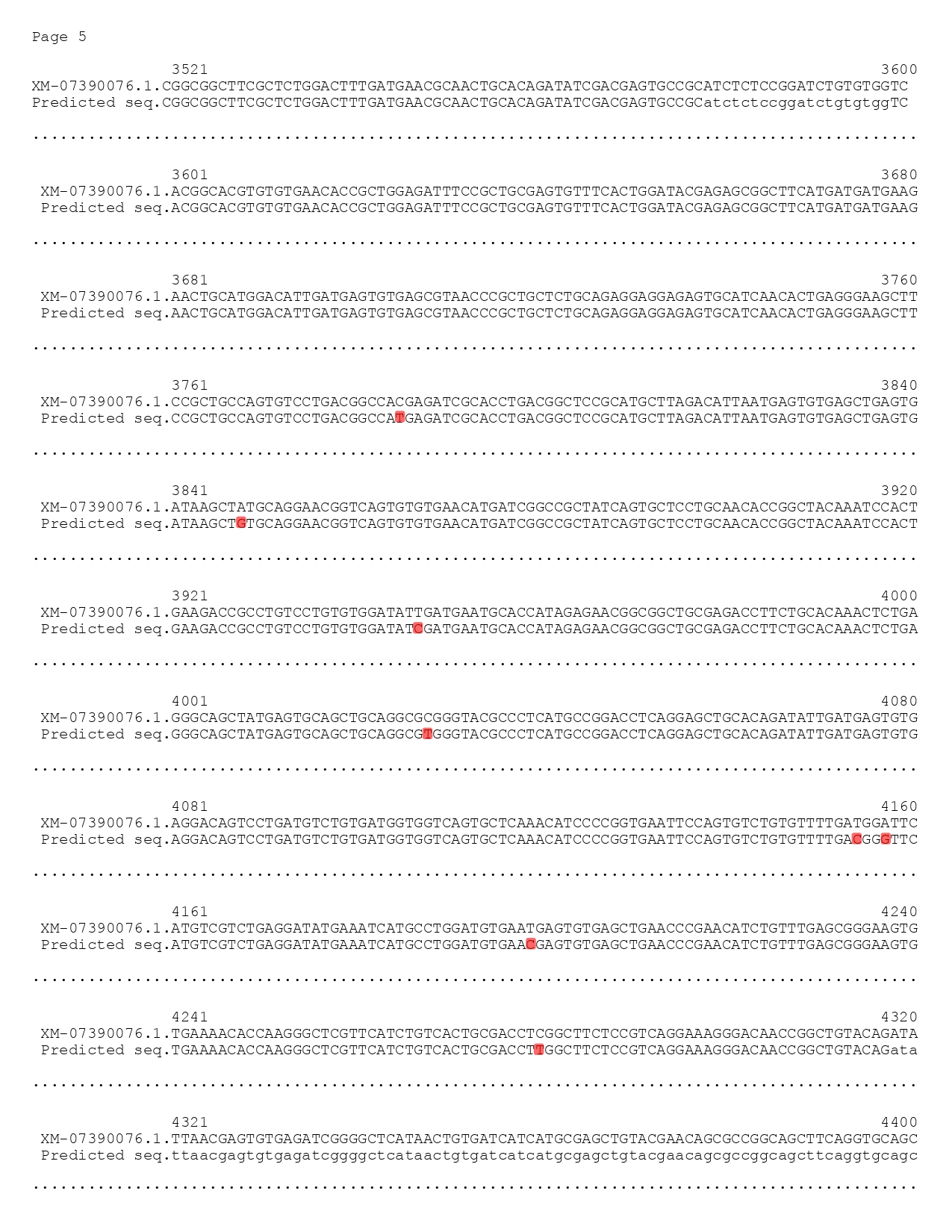
**

**
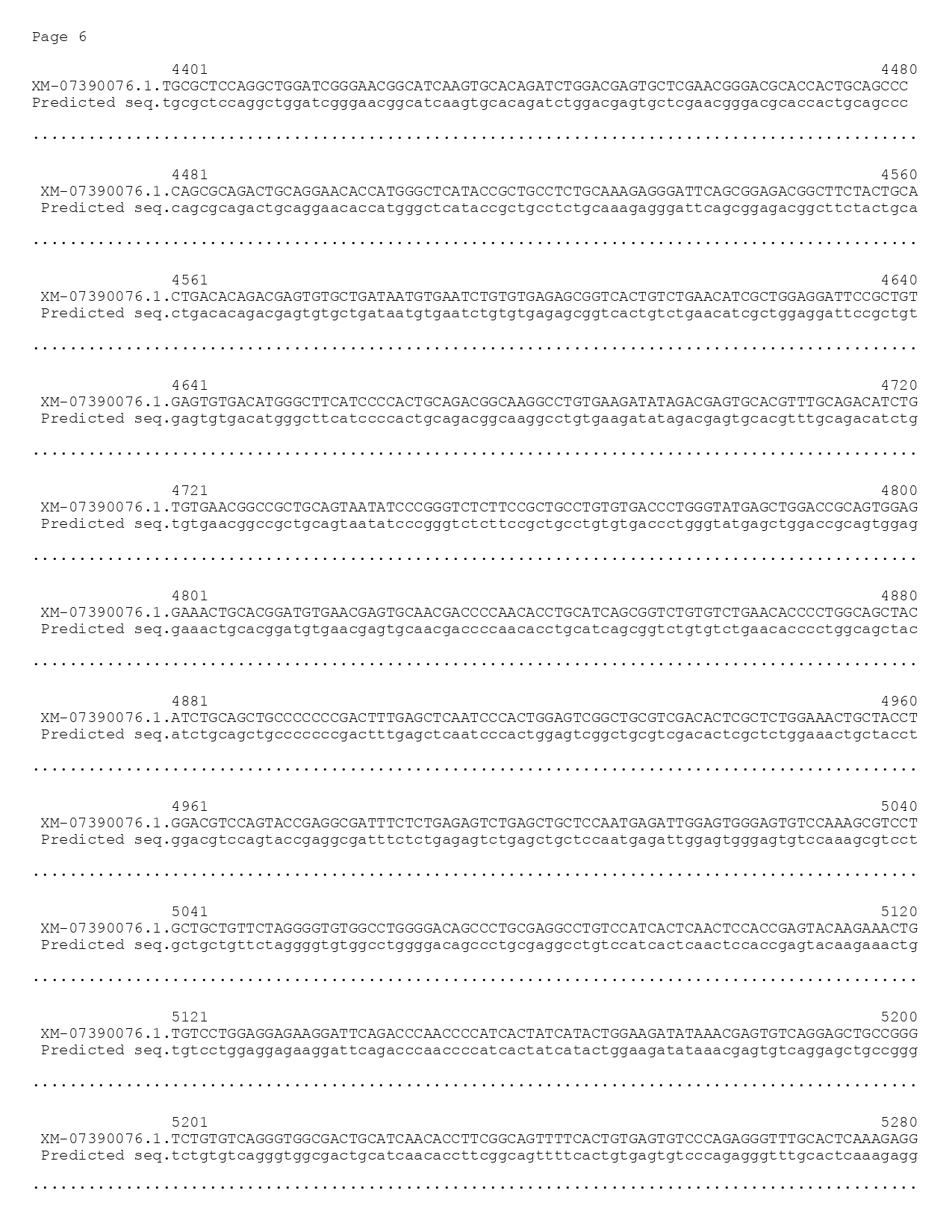
**

**
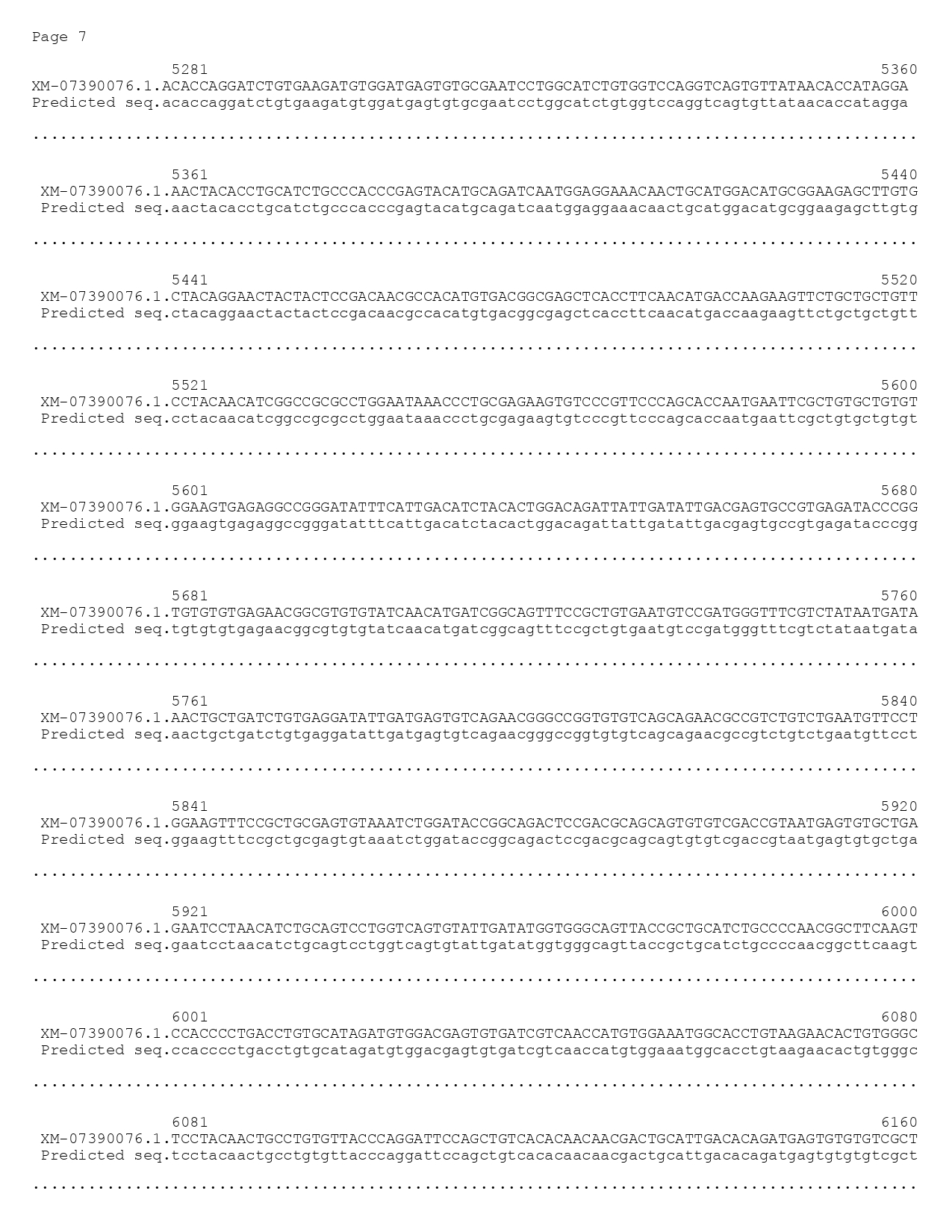
**

**
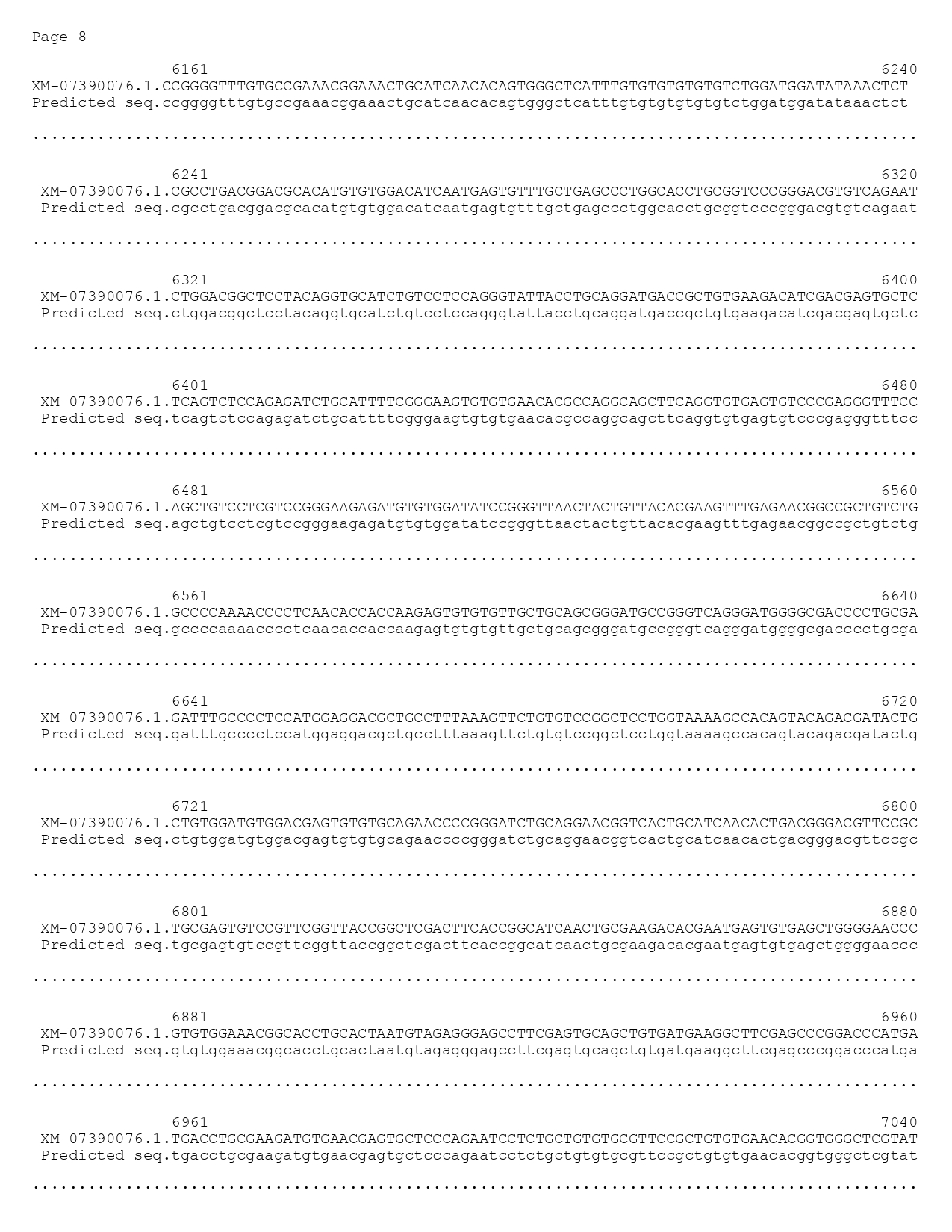
**

**
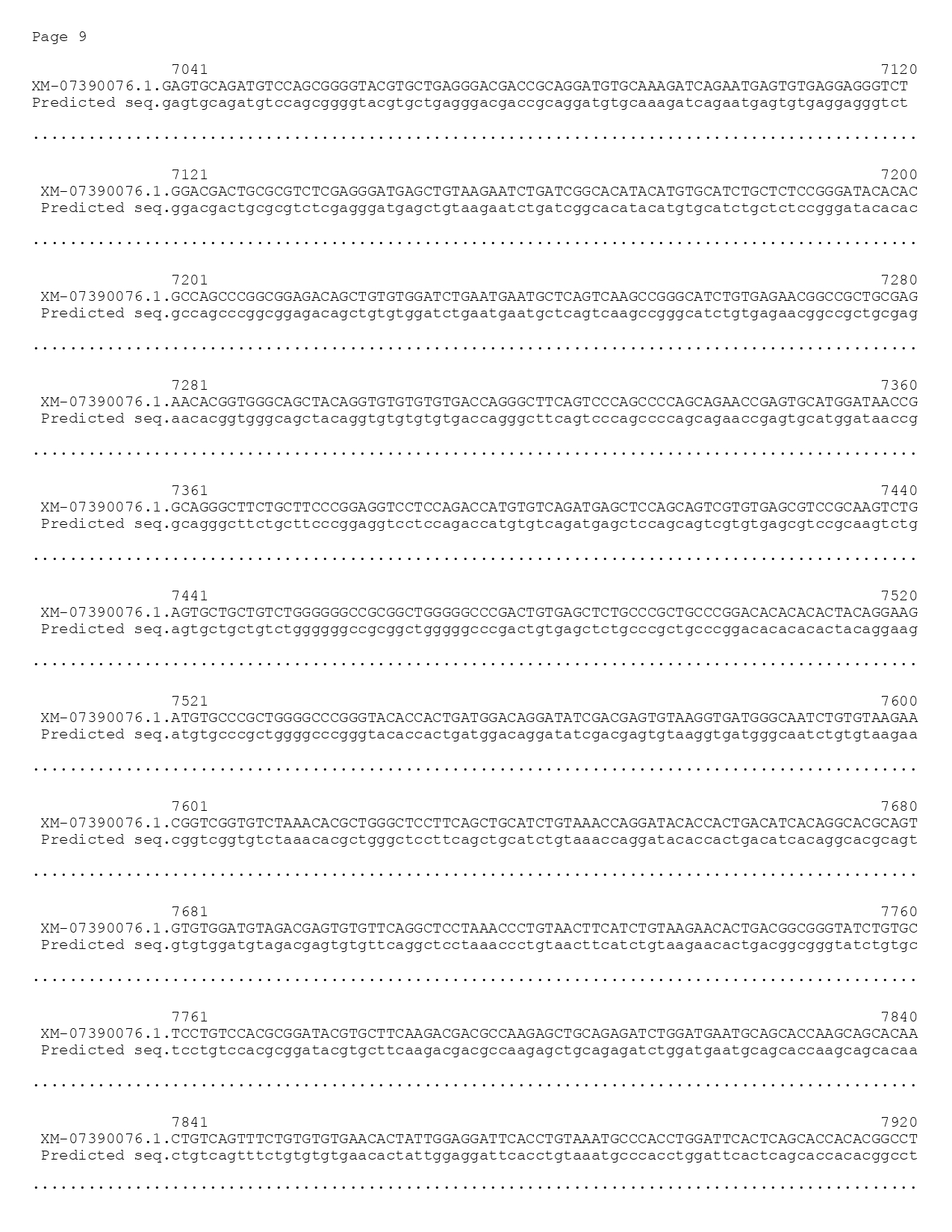
**

**
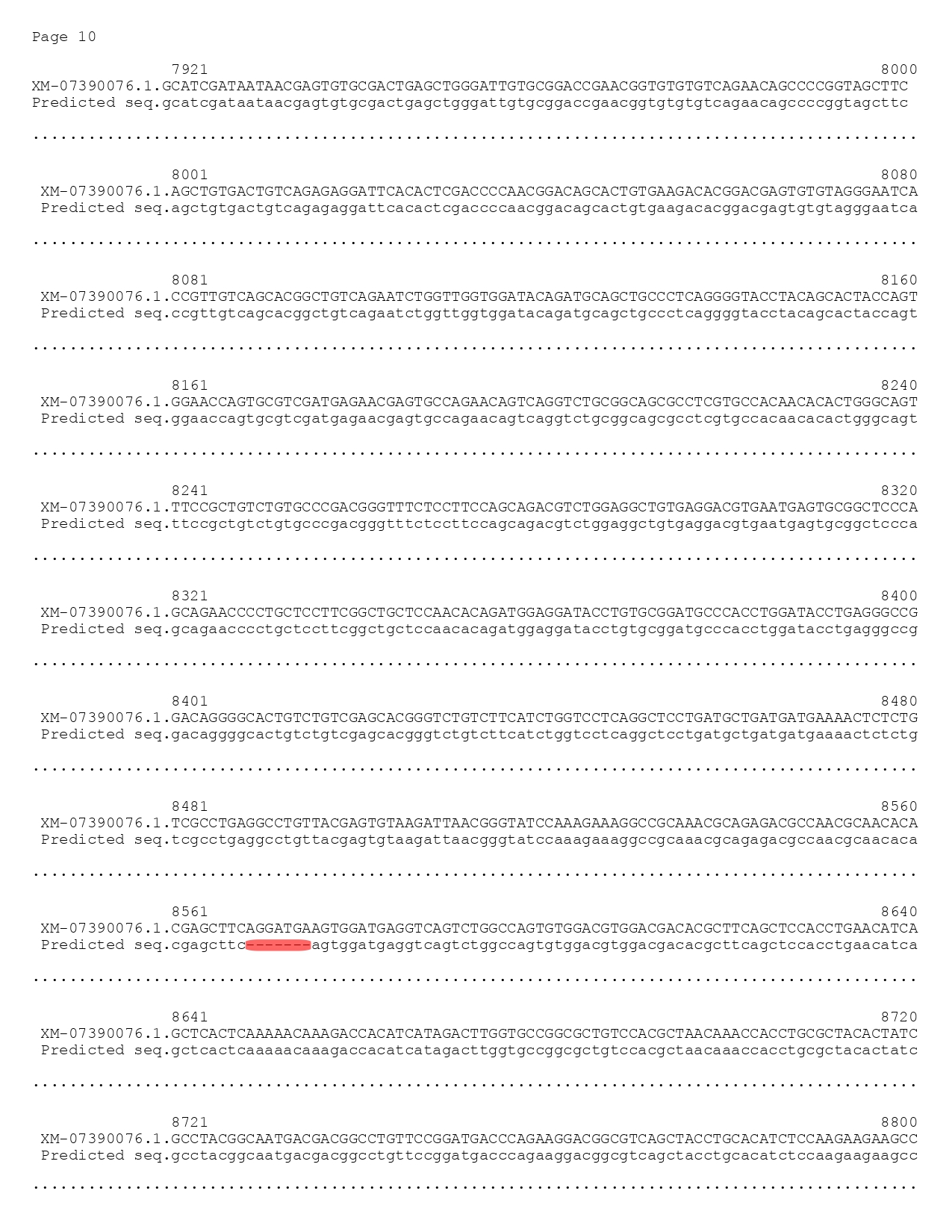
**

**
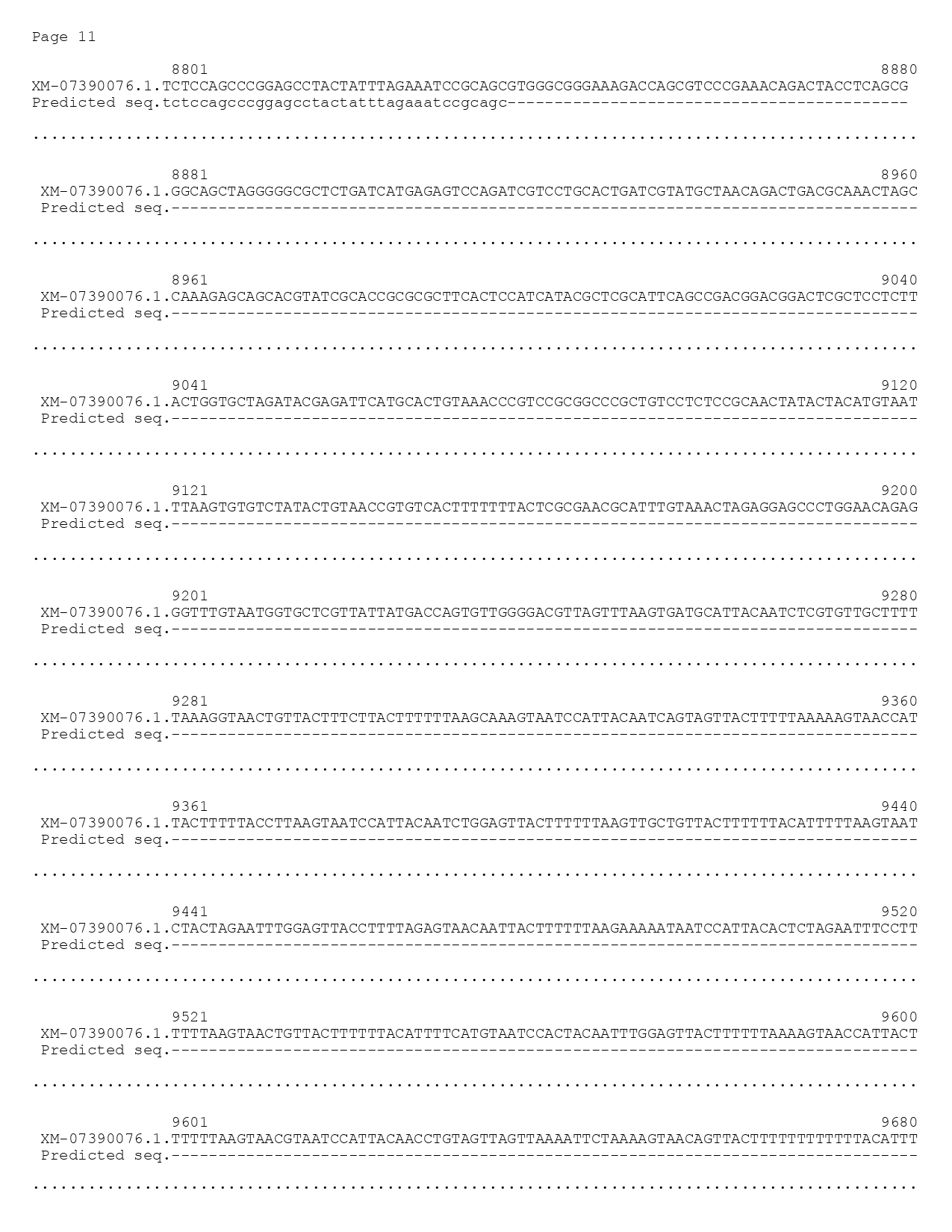
**

**
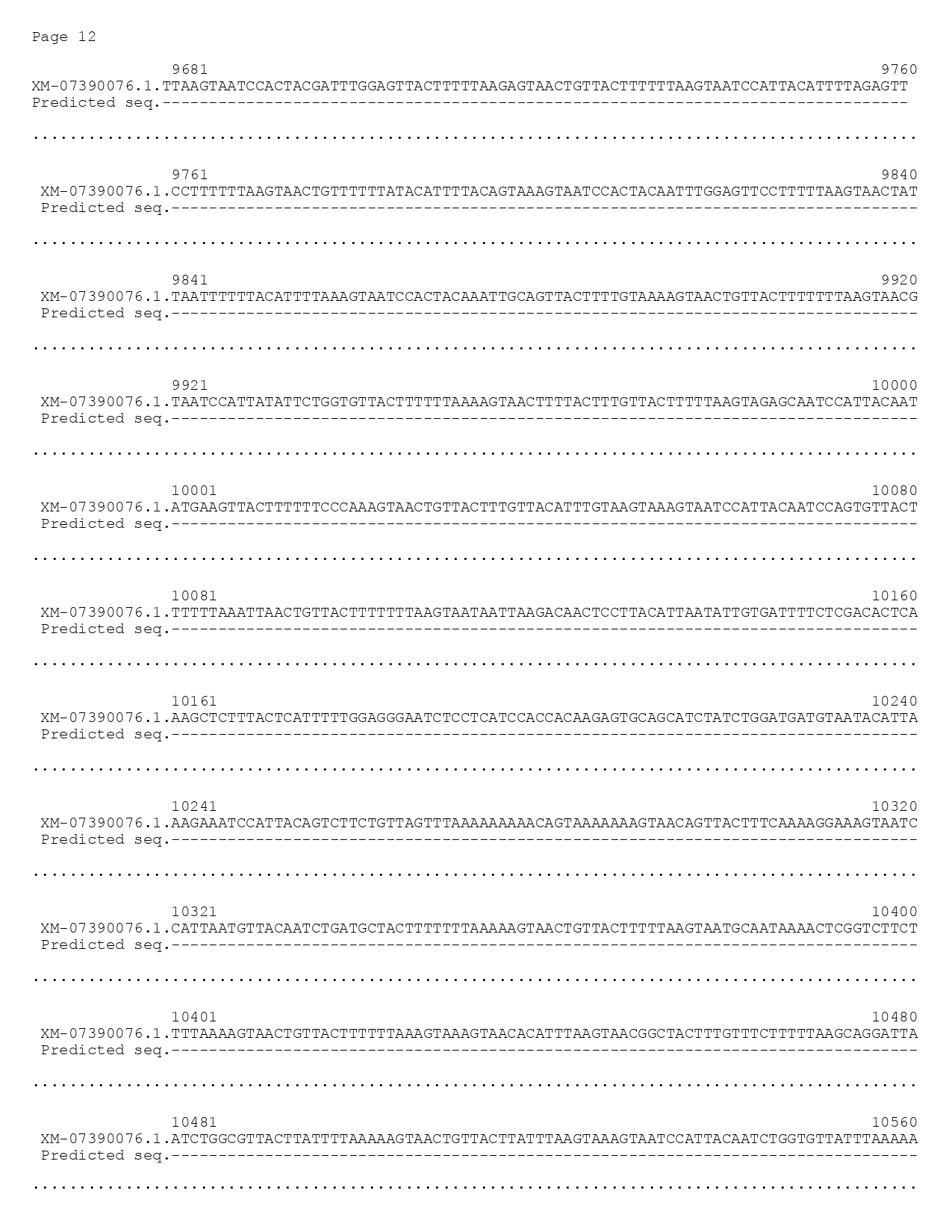
**

**
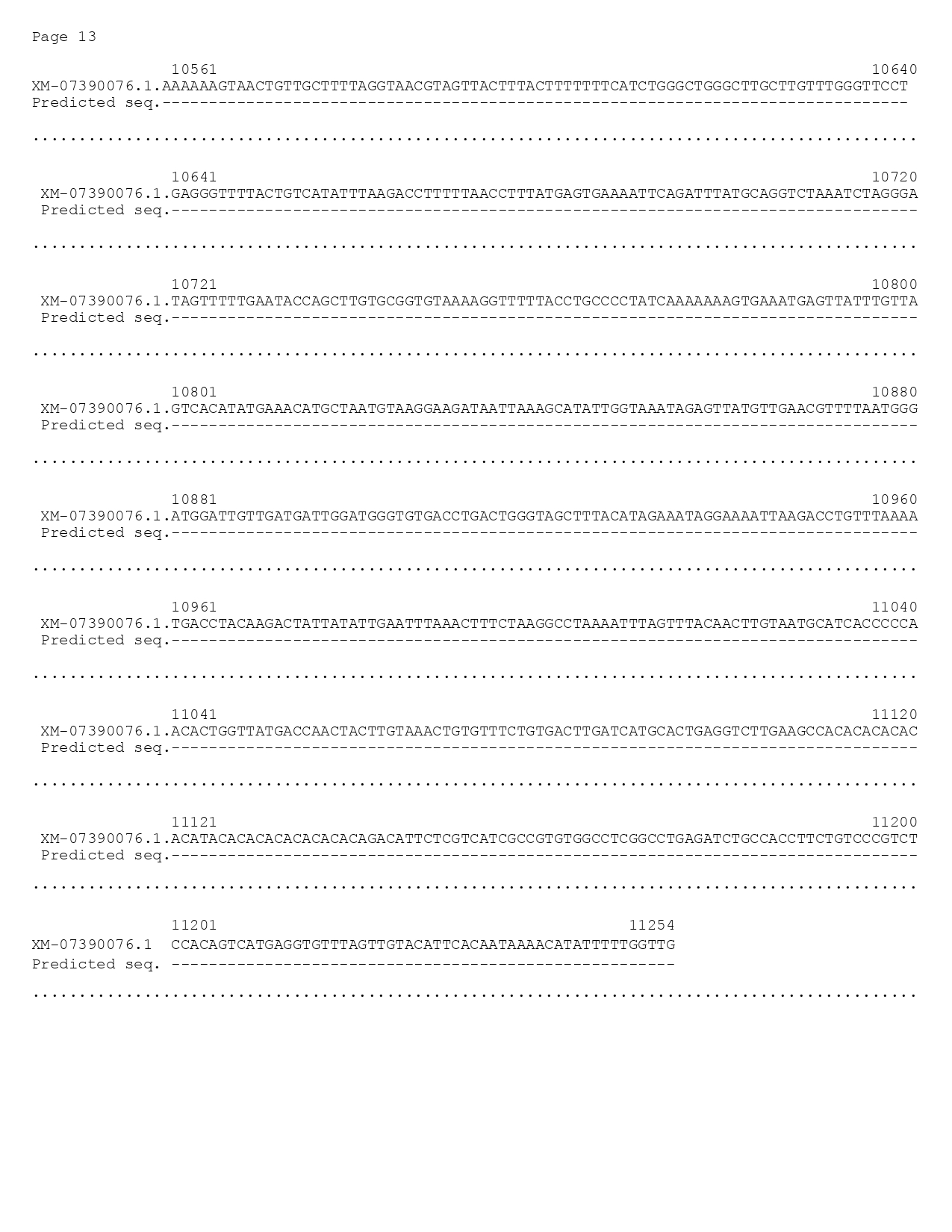
**

**Supplemental Figure 1.** Prediction of full-length *fbn1* mRNA sequence (XM_073930076.1).

Alignment of the *Danio rerio* *fbn1* mRNA sequence (XM_073930076.1) to our in-house predicted mRNA *fbn1* sequence through Sanger sequencing. Red indicates single-nucleotide polymorphisms (SNPs).

**
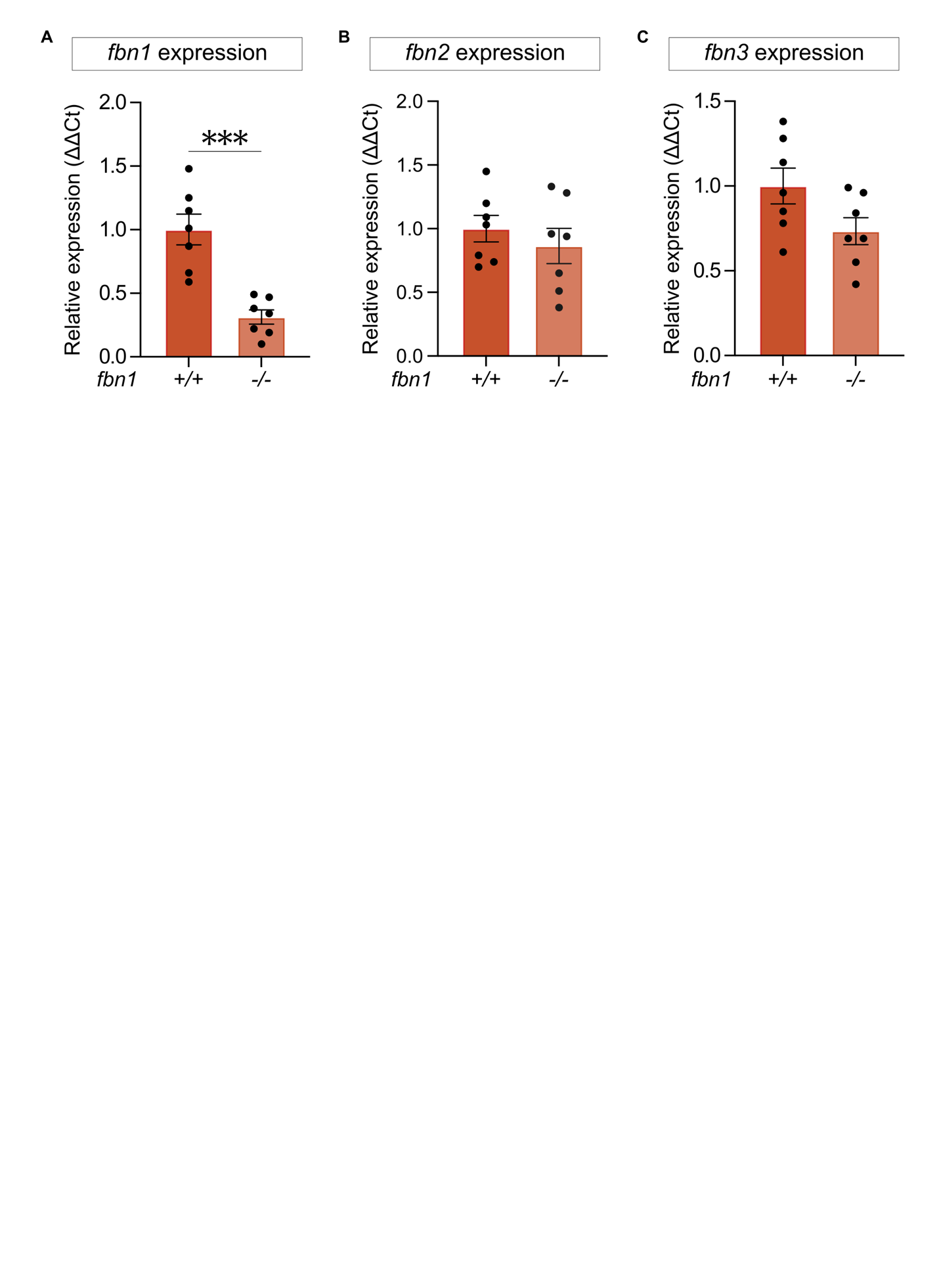
**

**Supplemental Figure 2.** *fbn1*, *fbn2* and *fbn3* expression in *fbn1^-/-^* zebrafish.

RT-qPCR analysis of *fbn1* **(A)**, *fbn2* **(B)** and *fbn3* **(C)** mRNA expression in 5 dpf WT and *fbn1^-/-^* (Cmg80) larvae (n = 7). Each data points represents the mean of 2 technical repeats. Data are expressed as mean ± SEM. Statistical analysis: unpaired t-test. ***p<0.001.


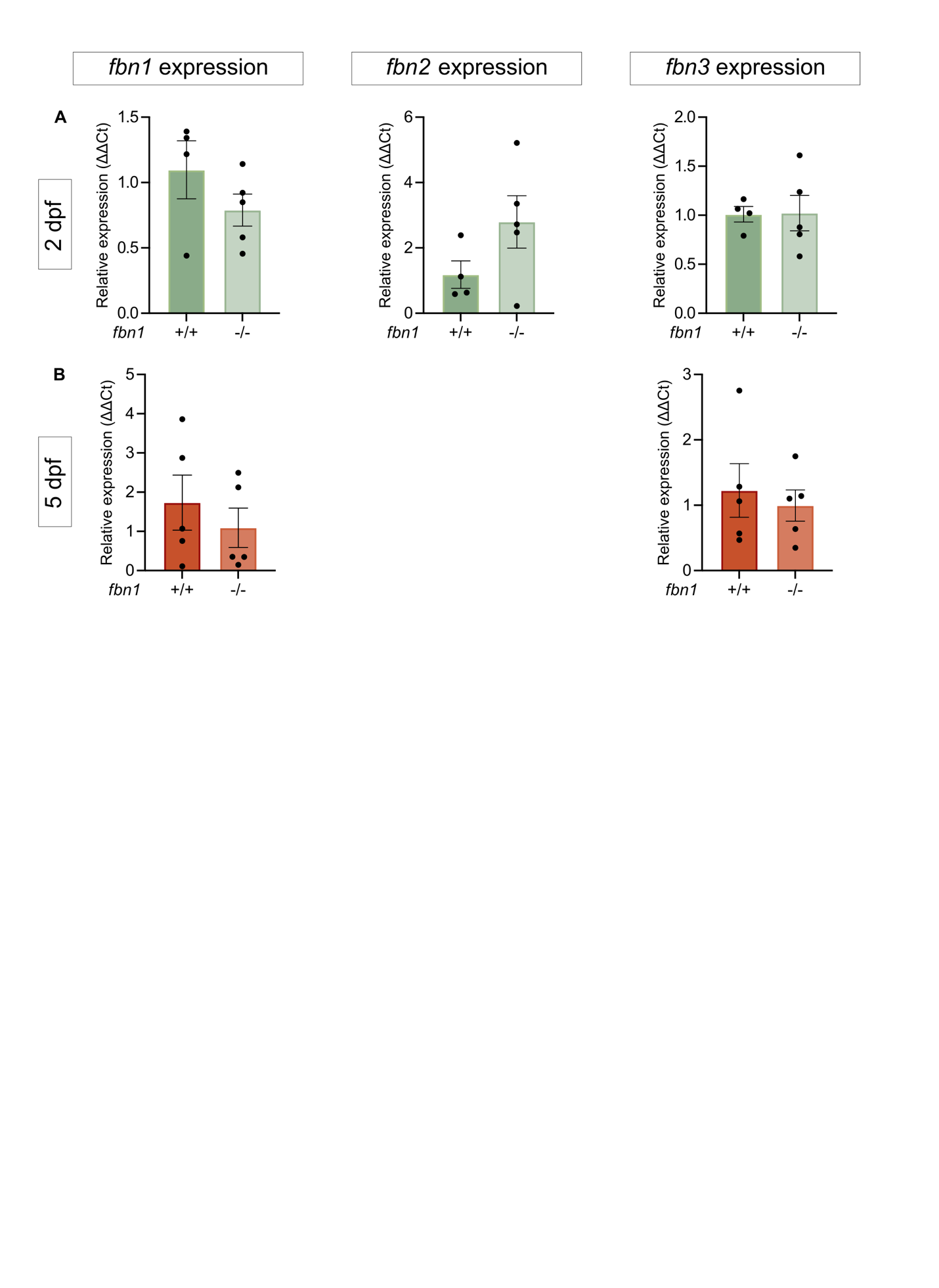


**Supplemental Figure 3.** *fbn1*, *fbn2* and *fbn3* expression in embryonic isolated hearts*.*

RT-qPCR analysis of *fbn1*, *fbn2* and *fbn3* mRNA expression in **(A)** 2 dpf and **(B)** 5 dpf isolated hearts of WT and *fbn1^-/-^* (Cmg80) larvae (n = 4-5). The expression level of *fbn2* was below the limit of detection in the 5 dpf samples. Each data point represents the mean of two technical repeats. Data are expressed as mean ± SEM. Statistical analysis: unpaired t-test.

**
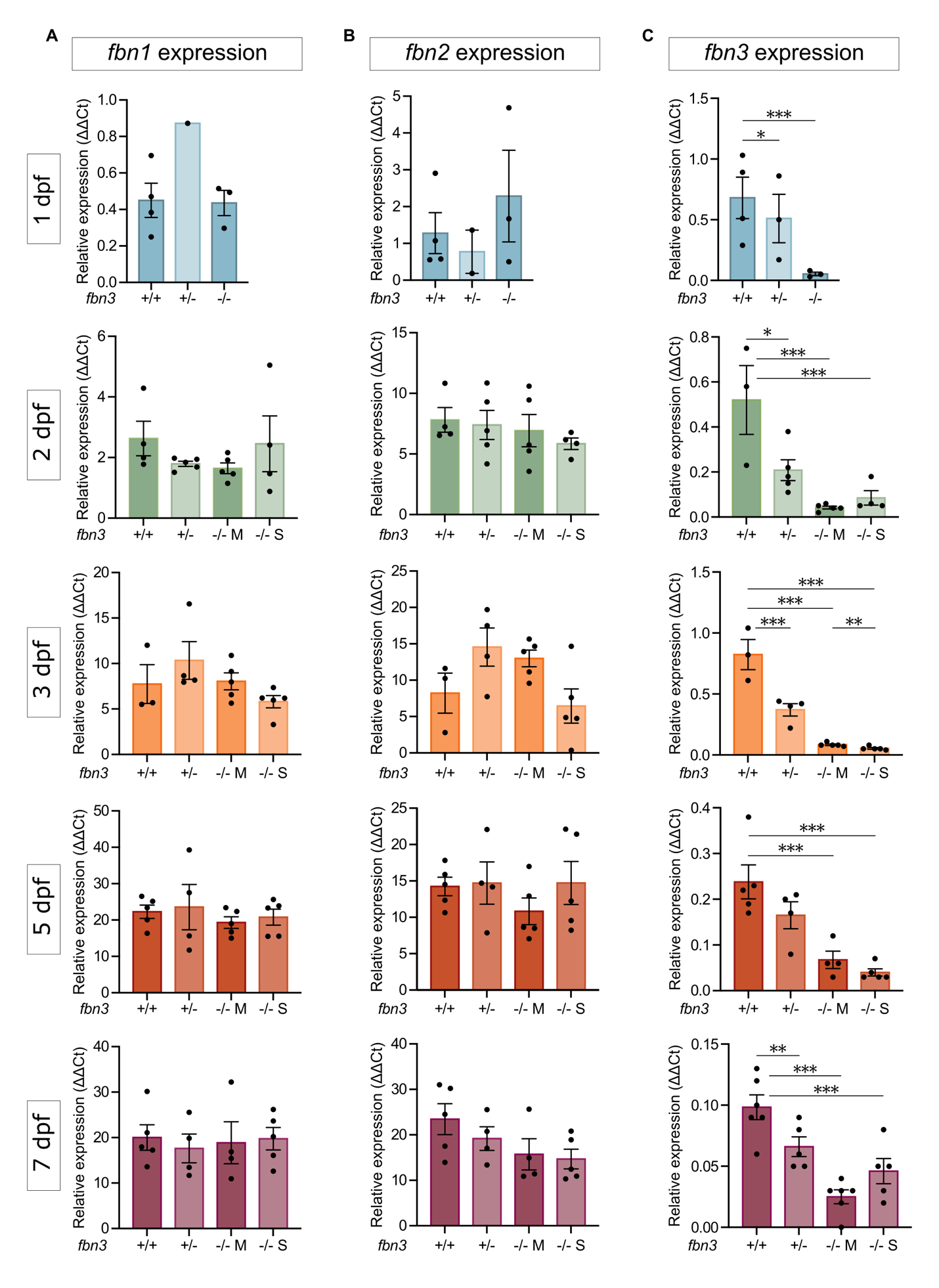
**

**Supplemental Figure 4.** *fbn1*, *fbn2* and *fbn3* expression patterns in *fbn3^-/-^* zebrafish.

RT-qPCR analysis of *fbn1* **(A)**, *fbn2* **(B)** and *fbn3* **(C)** mRNA expression in WT and *fbn3^-/-^* (mild and severe) on RNA extracted from whole embryos at 1, 2, 3, 5 and 7 dpf (n = 2-5 for each developmental stage). Each datapoint represents the mean of two technical replicates. Statistical analysis: One-way ANOVA followed by Tukey multiple comparison’s test on log-transformed data and tested for trend. Data are expressed as mean ± SEM. ***p<0.001, **p<0.01, *p<0.05. M = mild pericardial phenotype, S = severe pericardial phenotype.

**
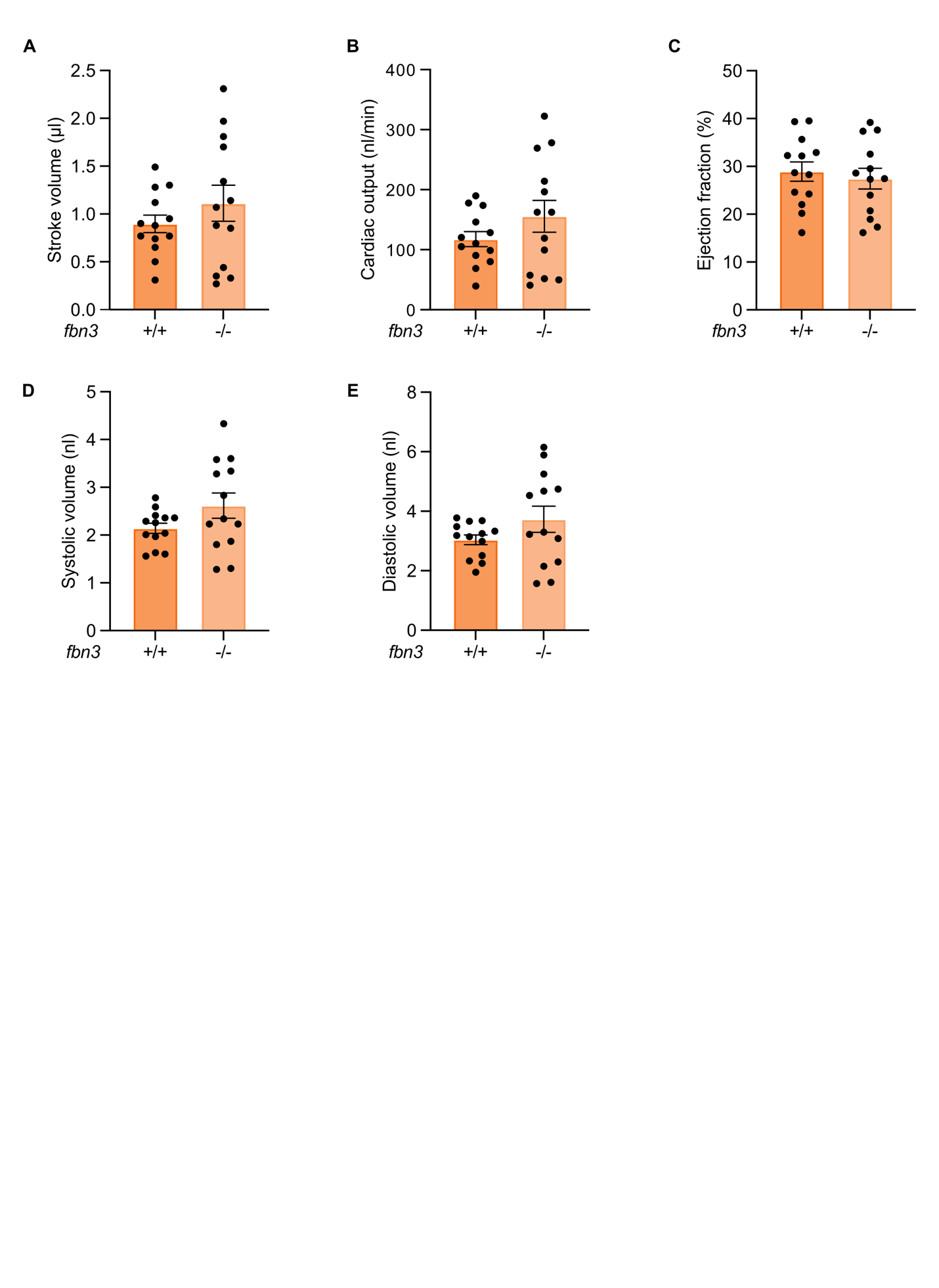
**

**Supplemental Figure 5.** Heart function analysis of 3 dpf *fbn3^-/-^* zebrafish.

Brightfield microscopy was used to quantify cardiac parameters in 3dpf *fbn3^-/-^* larvae and WT siblings: **(A)** stroke volume (mL), **(B)** cardiac output (nl/min), **(C)** ejection fraction (%), **(D)** systolic volume (Vsys, nl) and **(D)** diastolic volume (Vdias, nl) (n = 13). Data are expressed as mean ± SEM. Statistical test analysis: unpaired t-test. No statistical differences were reached.


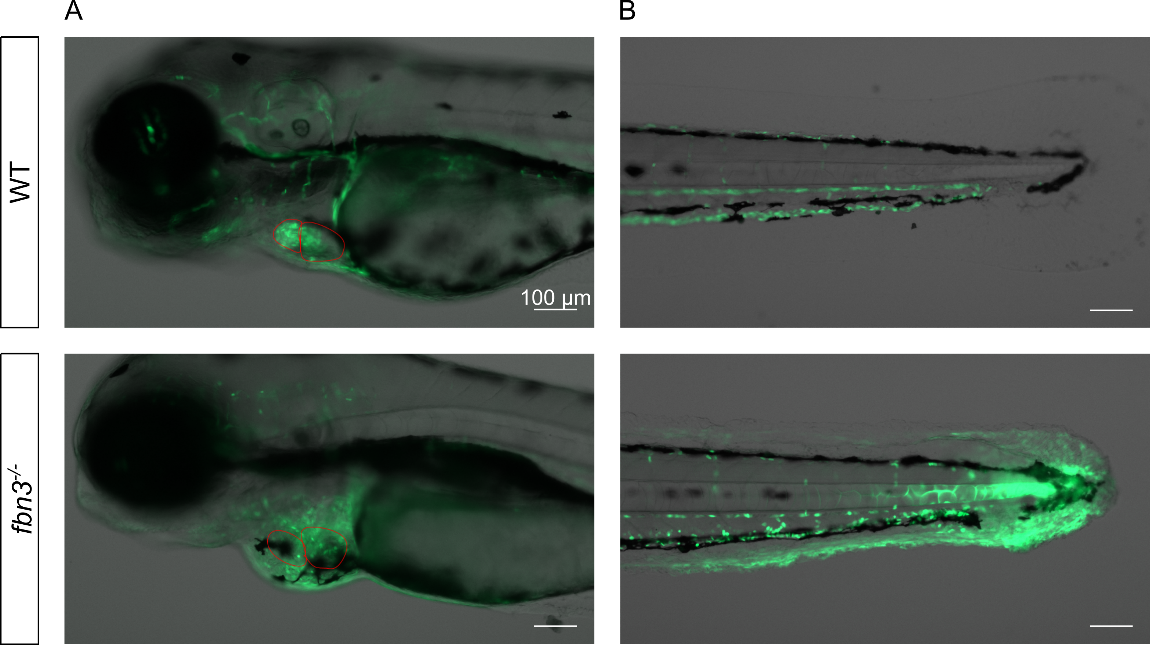


**Supplemental Figure 6.** Decreased endothelial integrity in *fbn3^-/-^* embryos.

Overlayed brightfield and fluorescent images *Tg(globin:GFP)* WT (top) and *fbn3^-/-^* embryos (bottom) at 72 hpf. Anterior views, including the heart (**A**), and posterior tail regions (**B**) are shown. The heart is outlined in red. *fbn3*^-/-^ embryos exhibit leakage of erythrocytes into the pericardial cavity and tail region, indicating compromised endothelial barrier integrity. Scale bar: 100 µm.


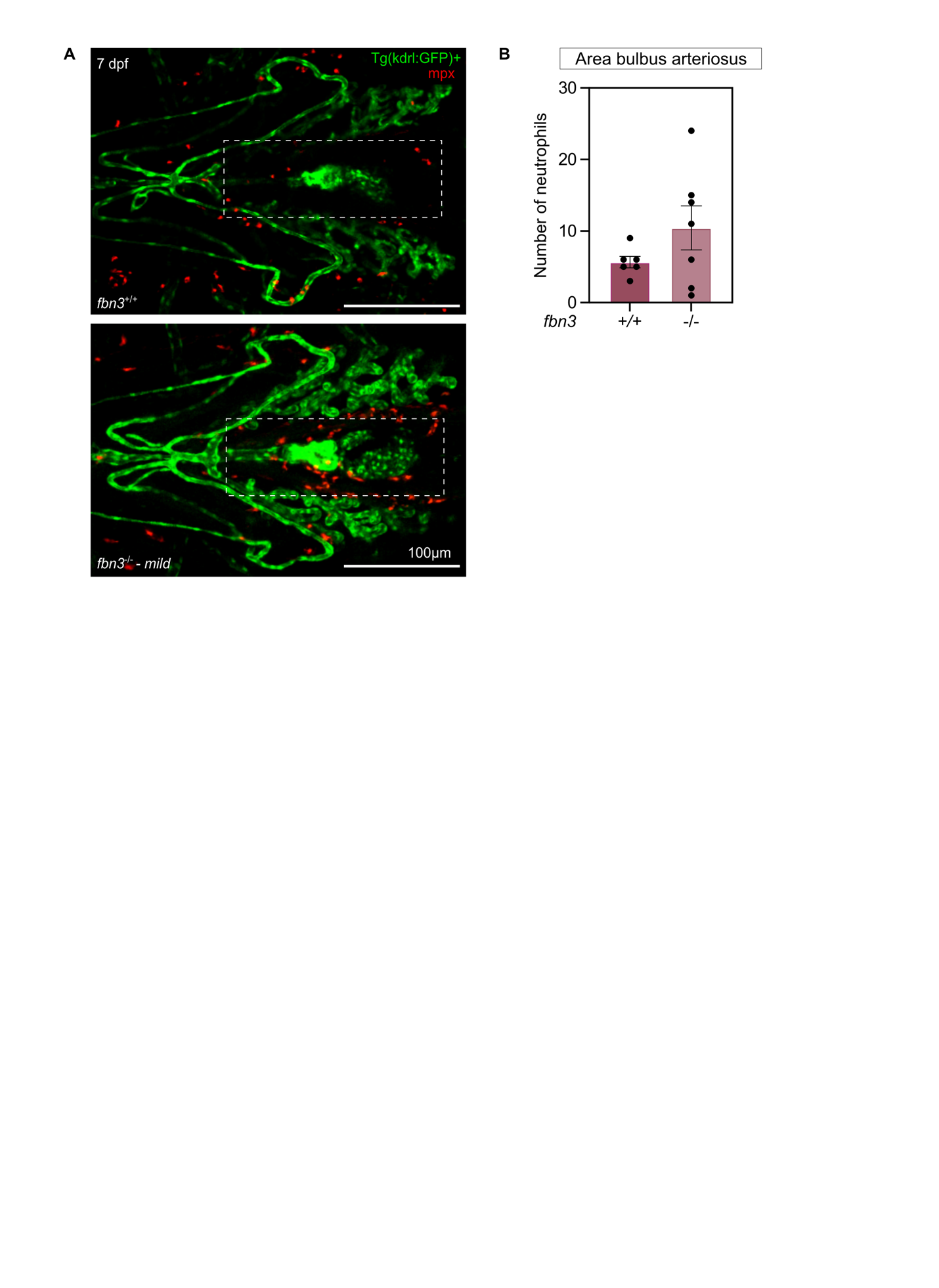


**Supplemental Figure 7.** Whole-mount neutrophil staining of 7 dpf *fbn3^-/-^* zebrafish.

**(A)** Confocal projection images of 7 dpf WT and *fbn3^-/-^* embryos whole-mount stained to visualize the immune response. Endothelial cells are labelled by the *Tg(kdrl:GFP)* reporter (green), and neutrophils are labelled by anti-mpx antibody staining (red). Scalebar: 100 µm. **(B)** Quantification of 7 dpf WT and *fbn3^-/-^* embryos (n = 6-7), showing the total number of neutrophils surrounding the bulbus arteriosus and heart. Statistical analysis: unpaired t-test. All data are expressed as a mean ± SEM.

**
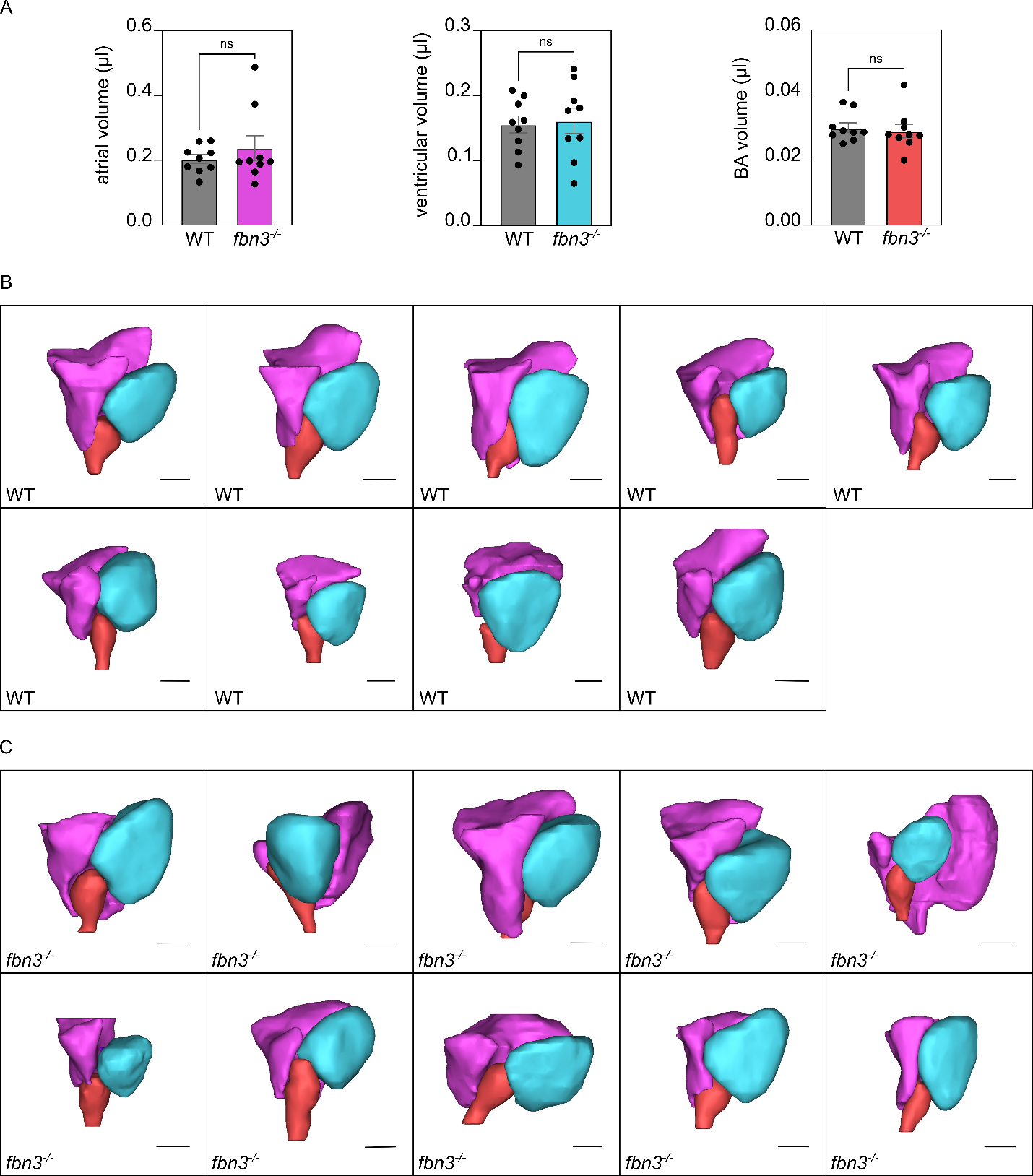
**

**Supplemental Figure 8.** 3D reconstructions of whole zebrafish hearts.

(**A**) Volumes of atrium, ventricle, and BA were obtained from 3D models based on synchrotron X-ray imaging. Reported volumes include combined lumen and wall. While no statistically significant differences were observed between WT and *fbn3^-/-^* zebrafish, a greater variability was evident in the mutant group. These differences are visually apparent in the 3D zebrafish heart models of WT (**B**) or *fbn3^-/-^* mutants (**C**). The atrium is displayed in pink, the ventricle in slightly translucent blue, and BA in red. Data are expressed as a mean ± SEM. ns = non-significant. Statistical test analysis: unpaired t-test. Scale bar: 500 µm. WT = wild type.

**
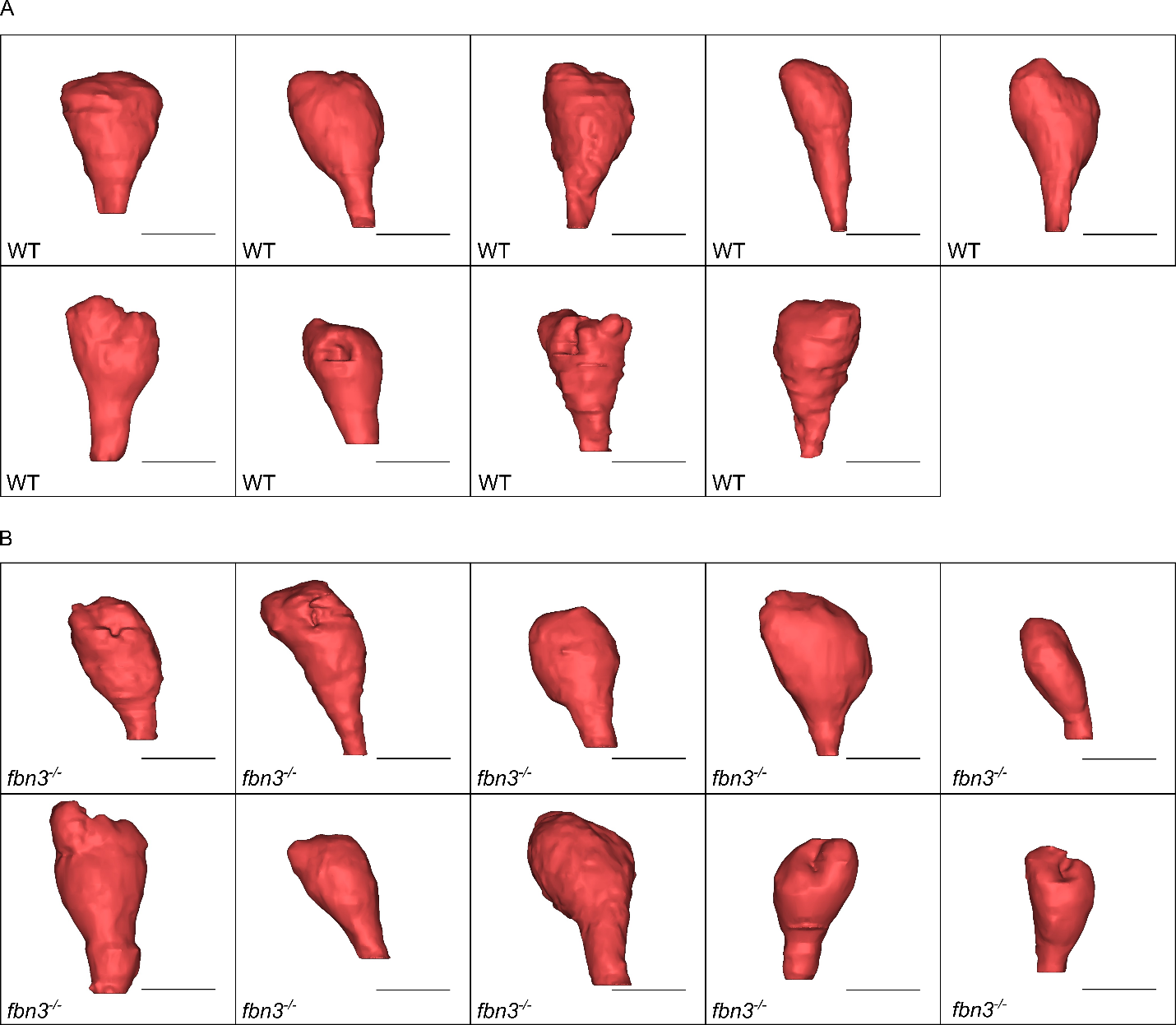
**

**Supplemental Figure 9.** 3D reconstructions of zebrafish BA.

Reconstructions based on synchrotron scans of BA from WT **(A)** and *fbn3^-/-^* mutants **(B)**. All images from the 3D models were captured from the same perspective. Scale bar: 100 µm. WT = wild type.

**
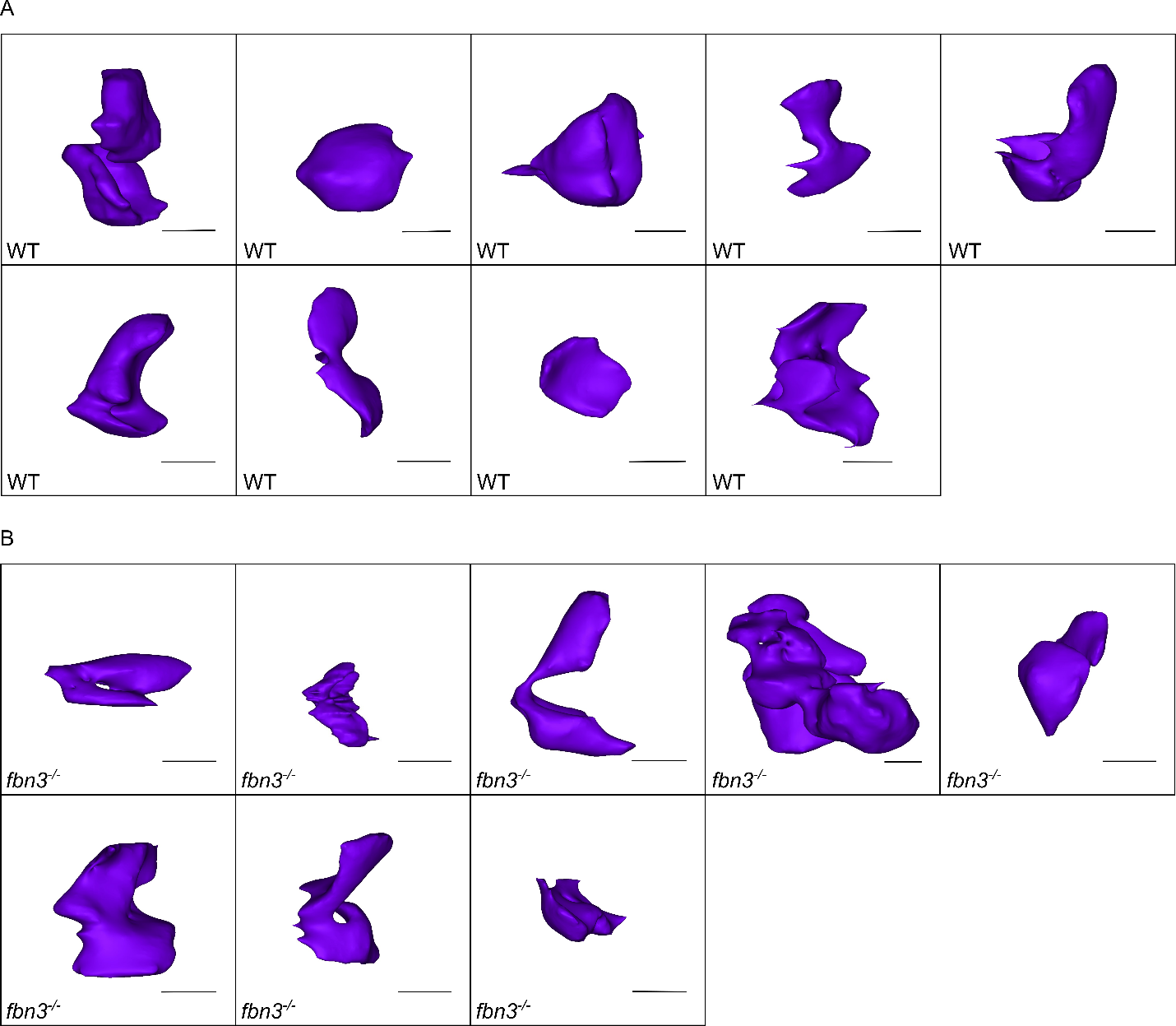
**

**Supplemental Figure 10.** 3D reconstructions of zebrafish AV valves.

Reconstructions based on synchrotron scans of AV valves from WT (**A**) and *fbn3^-/-^* mutants (**B**). The shape of the valve leaflets varied depending on their position during sample fixation, leading to inconsistencies. As a result, precise 2D dimensions (e.g. length and thickness) could not be accurately determined. All images from the 3D models were captured from the same perspective. Scale bar: 100 µm. WT = wild type.

**
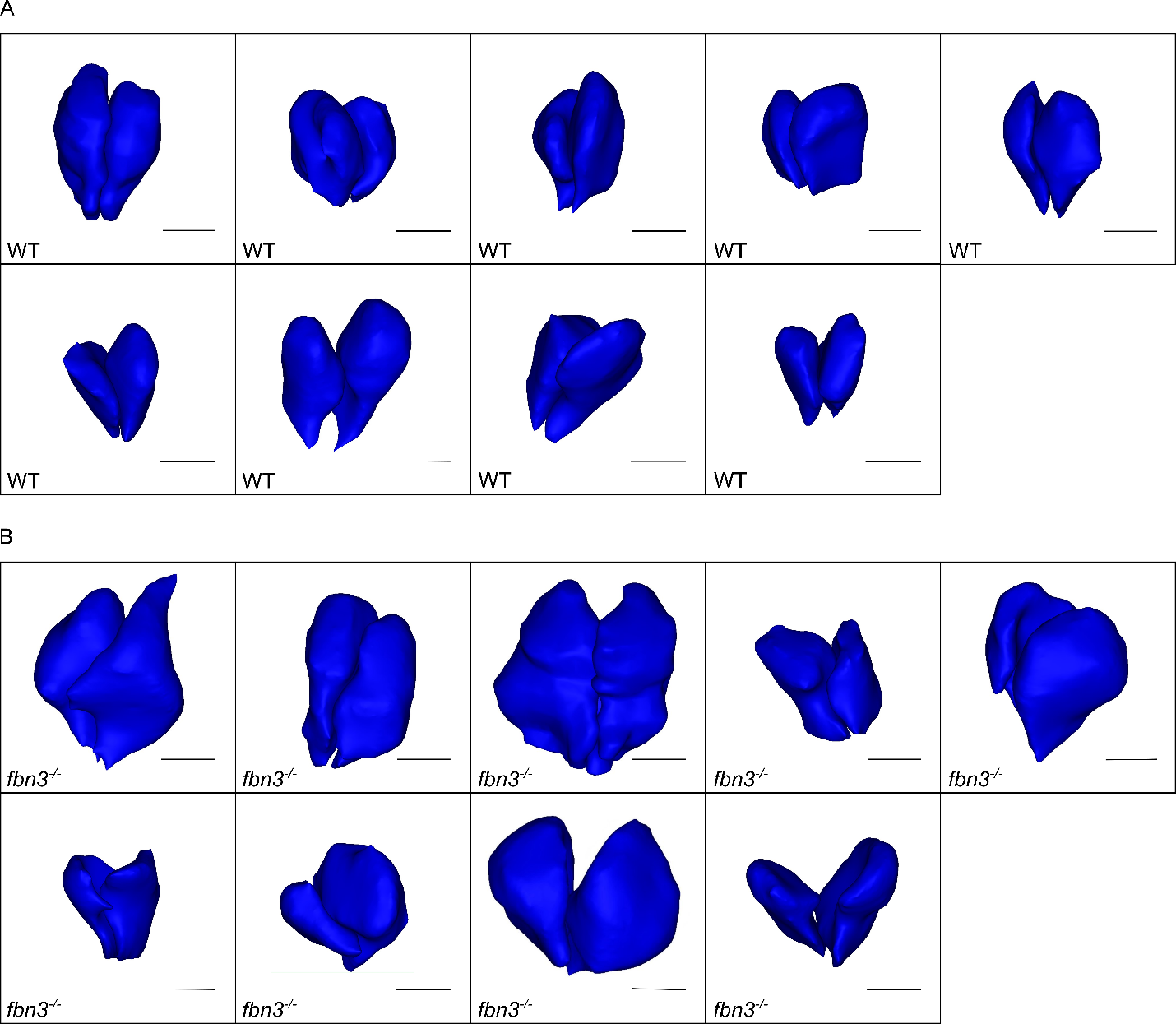
**

**Supplemental Figure 11.** 3D reconstructions of zebrafish BV valves.

Reconstructions based on synchrotron scans of BV valves from WT (**A**) and *fbn3^-/-^* mutants (**B**). The difference in leaflet sizes between WT and mutants can be visually observed. All images from the 3D models were captured from the same perspective. Scale bar: 100 µm. WT = wild type.


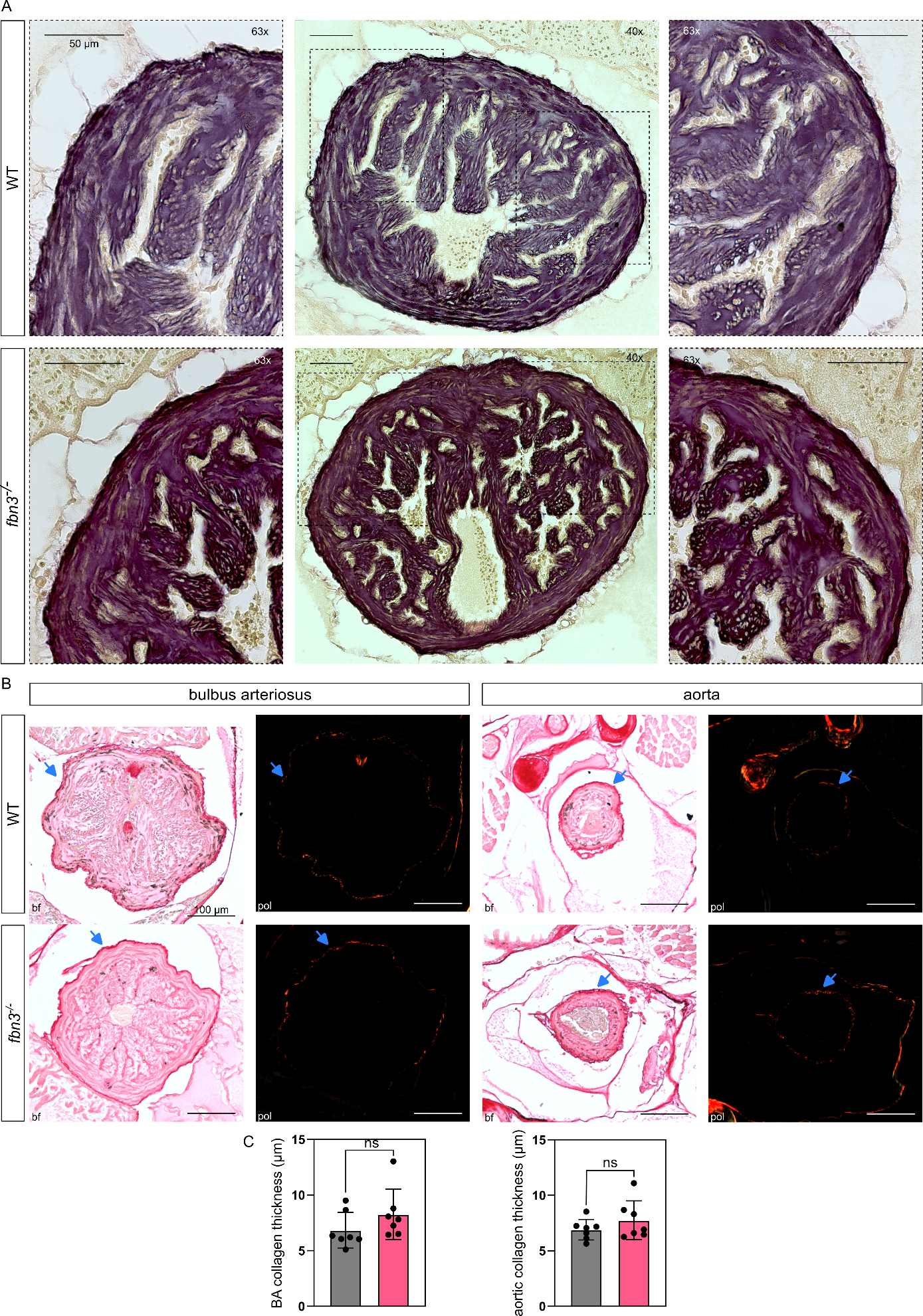


**Supplemental Figure 12.** Histological analysis of the BA in *fbn3^-/-^* zebrafish.

(**A**) High-magnification (40× and 60×) images of the BA from WT (top) and *fbn3^-/-^* zebrafish (bottom), stained for elastin (dark purple). (**B**) Sirius Red staining of collagen (dark red in brightfield image, bright red signal in polarized light image) in WT (top) and *fbn3^-/-^* zebrafish (bottom). Blue arrow: the collagen layer outlining the BA and aorta. (**C**) Quantification of collagen layer thickness in the BA and aorta (n = 7, average of three measurements on five slices per sample). Data are expressed as mean ± SEM. Statistical test analysis: unpaired t-test, ns = non-significant. bf = brightfield, pol = polarized light. Scale bars: 50 and 100 µm.

**
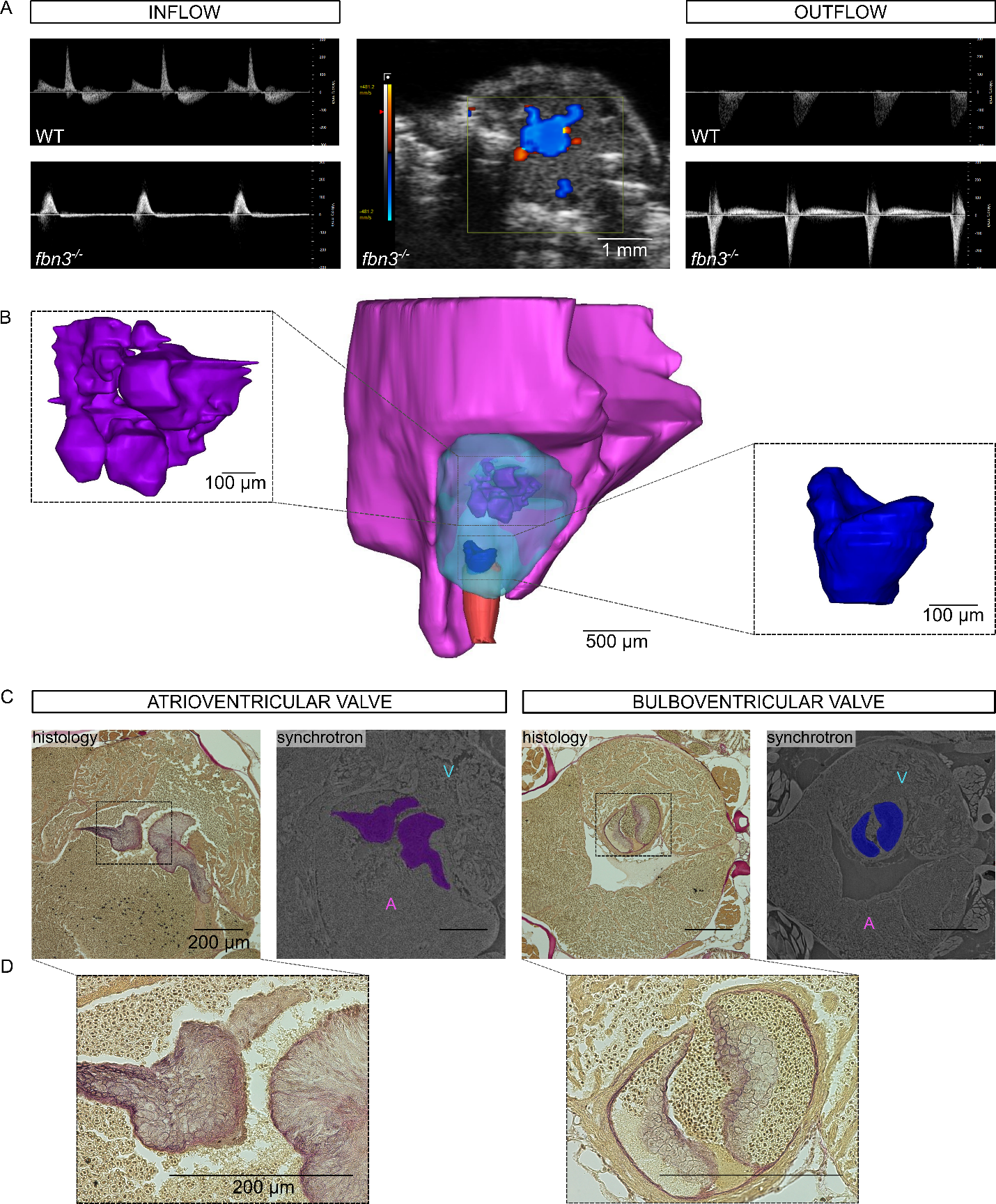
**

**Supplemental Figure 13.** Multimodal assessment of *fbn3^-/-^* zebrafish with an exceptionally severe cardiac phenotype.

(**A**) Pulse Wave Doppler images showing blood inflow into the ventricle (left) and outflow from the ventricle into BA (right), of WT zebrafish (top) in comparison to a highly abnormal *fbn3^-/-^* zebrafish (bottom). This mutant zebrafish exhibited pronounced regurgitation of the BV valve, which is evident in CFD imaging, showing simultaneous inflow (orange) and outflow (blue) at the same location (middle image). (**B**) 3D heart model of the same mutant zebrafish, created by synchrotron X-ray scanning. The atrium is displayed in pink, the ventricle in slightly translucent blue, BA in red, the AV valve in purple, and the BV valve in dark blue. The AV valve appears amorphic with various folded and fused segments, as well as being hypertrophic. The atrium is notably large, so much so that its uppermost segment was not captured in the synchrotron field of view. In contrast, the ventricle and BA are relatively small. (**C**) The same AV and BV valve sections were visualised using histological elastin staining (purple) and synchrotron imaging (purple and blue), demonstrating the similarities between the two techniques. (**D**) Higher magnification pictures of the cardiac valves. AV valve (left) shows excessive hypertrophy of the valve interstitial cells, which make up the interior of the leaflet. A = atrium, V = ventricle, WT = wild type.

# **Supplemental References**

1. Westerfield M. *The Zebrafish Book. A Guide for the Laboratory Use of Zebrafish (Danio Rerio)*. 4th ed. University of Oregon Press; 2000.

2. Andrews S. FastQC: a quality control tool for high throughput sequence data. 2010. Accessed June 9, 2025. https://www.bioinformatics.babraham.ac.uk/projects/fastqc/

3. Dobin A, Davis CA, Schlesinger F, et al. STAR: Ultrafast universal RNA-seq aligner. *Bioinformatics*. 2013;29(1):15-21. doi:10.1093/bioinformatics/bts635

4. Sayols S, Scherzinger D, Klein H. dupRadar: A Bioconductor package for the assessment of PCR artifacts in RNA-Seq data. *BMC Bioinformatics*. 2016;17(1). doi:10.1186/s12859-016-1276-2

5. Ewels P, Magnusson M, Lundin S, Käller M. MultiQC: Summarize analysis results for multiple tools and samples in a single report. *Bioinformatics*. 2016;32(19):3047-3048. doi:10.1093/bioinformatics/btw354

6. Liao Y, Smyth GK, Shi W. FeatureCounts: An efficient general purpose program for assigning sequence reads to genomic features. *Bioinformatics*. 2014;30(7):923-930. doi:10.1093/bioinformatics/btt656

7. Robinson MD, McCarthy DJ, Smyth GK. edgeR: A Bioconductor package for differential expression analysis of digital gene expression data. *Bioinformatics*. 2009;26(1):139-140. doi:10.1093/bioinformatics/btp616

8. Chen Y, Lun ATL, Smyth GK. From reads to genes to pathways: differential expression analysis of RNA-Seq experiments using Rsubread and the edgeR quasi-likelihood pipeline. *F1000Res*. 2016;5:1438. doi:10.12688/f1000research.8987.1

9. Yu G, Wang LG, Han Y, He QY. ClusterProfiler: An R package for comparing biological themes among gene clusters. *OMICS*. 2012;16(5):284-287. doi:10.1089/omi.2011.0118

10. Vanhauwaert S, Lefever S, Coucke P, et al. RT-qPCR gene expression analysis in zebrafish: Preanalytical precautions and use of expressed repetitive elements for normalization. *Methods Cell Biol*. 2016;135:329-342. doi:10.1016/bs.mcb.2016.02.002

11. Lombardo VA, Otten C, Abdelilah-Seyfried S. Large-scale zebrafish embryonic heart dissection for transcriptional analysis. *Journal of Visualized Experiments*. 2015;(95). doi:10.3791/52087

12. Werdich AA, Brzezinski A, Jeyaraj D, et al. The zebrafish as a novel animal model to study the molecular mechanisms of mechano-electrical feedback in the heart. *Prog Biophys Mol Biol*. 2012;110(2-3):154-165. doi:10.1016/j.pbiomolbio.2012.07.006

13. Forster B, Van De Ville D, Berent J, Sage D, Unser M. Complex wavelets for extended depth-of-field: A new method for the fusion of multichannel microscopy images. *Microsc Res Tech*. 2004;65(1-2):33-42. doi:10.1002/jemt.20092

14. Shin JT, Pomerantsev E V., Mably JD, MacRae CA. High-resolution cardiovascular function confirms functional orthology of myocardial contractility pathways in zebrafish. *Physiol Genomics*. 2010;42(2):300-309. doi:10.1152/physiolgenomics.00206.2009

15. Van Impe M, Caboor L, Deleeuw V, et al. Application of an automated analysis framework for pulsed-wave Doppler cardiac ultrasound measurements to generate reference data in adult zebrafish. *Am J Physiol Regul Integr Comp Physiol*. 2023;325(6):R782-R796. doi:10.1152/ajpregu.00103.2023

16. Niimi A. Relationship of body surface area to weight in fishes. *Can J Zool*. 1975;53(8):1192-1194. doi:0.1139/z75-141

17. Mallory FB. *Pathological Tecniques*. Hafner Publishing Co.; 1961.

18. Rittié L. *Fibrosis*. Vol 1627. (Rittié L, ed.). Springer New York; 2017. doi:10.1007/978-1-4939-7113-8

19. Paganin D, Mayo SC, Gureyev TE, Miller PR, Wilkins SW. Simultaneous phase and amplitude extraction from a single defocused image of a homogeneous object. *J Microsc*. 2002;206(1):33-40. doi:10.1046/j.1365-2818.2002.01010.x

20. Van Impe M, Caboor L, Deleeuw V, et al. Fluid-Structure Interaction Modeling of the Aortic Hemodynamics in Adult Zebrafish: A Pilot Study Based on Synchrotron X-Ray Tomography. *IEEE Trans Biomed Eng*. 2023;70(7):2101-2110. doi:10.1109/TBME.2023.3236488
